# Safety and immunogenicity of a BPL-inactivated, whole-virus, universal influenza vaccine: a randomized, double-blinded, double-dummy, placebo-controlled, phase 1 clinical trial

**DOI:** 10.1038/s41467-026-78583-w

**Published:** 2026-10-12

**Authors:** Luca T. Giurgea, Alison Han, Lindsay M. Czajkowski, Holly Ann Baus, Gregory Mak, Matthias Lingemann, Ian Bellayr, Adriana Cervantes-Medina, Monica Gouzoulis, Kimberly Adao, Jenna Sherry, Rani Athota, Jackqueline Ardo, Robert Daniels, Viviane Callier, Jaekeun Park, Sally Hunsberger, Mattia Bonsignori, John H. Powers, John C. Kash, Jeffery K. Taubenberger, Matthew J. Memoli

**Affiliations:** 1 Clinical Studies Unit, Laboratory of Infectious Diseases, Division of Intramural Research, National Institute of Allergy and Infectious Diseases, National Institutes of Health, Bethesda, MD USA; 2 Translational Immunobiology Unit, Laboratory of Infectious Diseases, Division of Intramural Research, National Institute of Allergy and Infectious Diseases, National Institutes of Health, Bethesda, MD USA; 3 Laboratory of Infectious Diseases, Division of Intramural Research, National Institute of Allergy and Infectious Diseases, National Institutes of Health, Bethesda, MD USA; 4 Viral Pathogenesis and Evolutions Section, Laboratory of Infectious Diseases, Division of Intramural Research, National Institute of Allergy and Infectious Diseases, National Institutes of Health, Bethesda, MD USA; 5 Division of Viral Products, Center for Biologics Evaluation and Research, Food and Drug Administration, Silver Spring, MD USA; 6 Clinical Monitoring Research Program Directorate, Frederick National Laboratory for Cancer Research, Frederick, MD USA; 7 Department of Veterinary Medicine, University of Maryland, College Park, MD USA; 8 Virginia-Maryland College of Veterinary Medicine, College Park, MD USA; 9 Biostatistics Research Branch, Office of Biostatistics Research, National Institute of Allergy and Infectious Diseases, National Institutes of Health, Bethesda, MD USA; 10 Laboratory of Translational Immunobiology, Department of Cellular, Computational and Integrative Biology, University of Trento, Trento, Italy; 11 Office of the Director, National Institute of Allergy and Infectious Diseases, National Institutes of Health, Bethesda, MD USA; 12 Office of the Director, National Institutes of Health, Bethesda, MD USA

**Keywords:** Inactivated vaccines, Influenza virus, Drug development

## Abstract

Influenza inflicts substantial burden of disease, but current vaccines have limited breadth of protection against strains and subtypes not in the vaccine. BPL-1357, a whole-virus, inactivated vaccine containing four wild-type, low-pathogenicity avian influenza viruses has demonstrated remarkable protection in animal models against a wide range of influenza viruses. In this phase I trial, forty-five healthy participants were randomized to receive BPL-1357 intramuscularly (IM), intranasally (IN), or placebo, in a prime and boost strategy, 28-days apart. The primary endpoints consisted of adverse events (AEs) and serious adverse events (SAEs) from enrollment through 28 days after the boost. The secondary endpoints were AEs and SAEs through 182 days after the boost and measurement of serum IgG and nasal IgA titers against hemagglutinin and neuraminidase vaccine antigens. Adverse effects were mostly mild and, other than mild injection-site pain more frequently observed in the IM group, occurred in a minority of patients. IM BPL-1357 induced higher serum IgG titers against H3, H5, and H7 relative to placebo, with 71.4%, 71.4%, and 85.7% of participants demonstrating a response. IM BPL-1357 vaccinated participants also experienced serum neutralizing and NA-specific responses, but no mucosal IgA responses against HA or NA. Subtle mucosal and serum responses were observed after IN vaccination, similar to preclinical experiments. These results suggest that BPL-1357 is well tolerated and immunogenic, particularly when given IM, but more work is needed to assess other dose levels, further characterize induced immunity, and evaluate efficacy against influenza infection and disease (NCT05027932).

## Abstract (web summary)

BPL-1357 has shown broad protection in animal models against influenza viruses. The authors demonstrate safety of the vaccine given intramuscularly or intranasally in healthy adults in a phase I trial and characterize the elicited immune responses.

## Introduction

Influenza A has been responsible for four pandemics over the last 108 years, with the 1918 pandemic causing approximately 50 million deaths worldwide^1^. After each pandemic, the virus responsible established endemicity, causing further morbidity and mortality^2^. Currently available influenza vaccines provide suboptimal effectiveness against seasonal influenza. Estimates of protection largely depend on the match between a vaccine developed ahead of time and an unpredictable, mutating virus. Even when the vaccine is well-matched, many patients are left unprotected. The highest estimates of vaccine effectiveness against laboratory-confirmed influenza reach 60%, a number that has not markedly changed since the development of the first influenza vaccines 8 decades ago^3,4^. Furthermore, current seasonal influenza vaccines can contain suboptimal neuraminidase (NA) and T-cell stimulating antigens, thereby offering limited breadth of protection against most influenza viruses^5–7^. With the expectation that future influenza pandemics are inevitable and seasonal influenza is responsible for a significant cumulative burden of disease, development of a universal influenza vaccine capable of broad protection against a plurality of group I and II influenza viruses has been a research priority^8^. Such a vaccine would minimize the impact of a future influenza pandemic and provide more durable protection against seasonal influenza.

BPL-1357 is a whole-virus, β-propiolactone (BPL)-inactivated vaccine containing four wild-type, low-pathogenicity avian influenza viruses: H1N9, H3N8, H5N1, and H7N3. The inclusion of the whole virus in a vaccine has numerous biological advantages. The presence of multiple surface antigens, including both haemagglutinin (HA) and NA in their naturally occurring structural arrangements, can elicit multifaceted responses resilient to viral escape mutations. The presence of highly conserved internal proteins can stimulate T-cells. Immunogenicity is improved due to the presence of viral nucleic acids and uptake of the particles by immune cells. In addition, BPL-inactivated and whole-virus influenza vaccines have a long history of safety and tolerability and can utilize existing manufacturing and distribution systems, including in low-resource settings, due to their temperature stability.

The selection of viruses in BPL-1357 includes four distinct HA subtypes, two from each of the major phylogenetic groups, representing both seasonal influenza subtypes (H1 and H3) and the two avian influenza subtypes (H5 and H7) responsible for the largest human outbreaks with historically high case fatality ratios^9,10^. The vaccine strain selection also covers four distinct NA subtypes, two from each of the major phylogenetic groups. In mice and ferrets, BPL-1357 given as a prime-boost regimen intramuscularly (IM) or intranasally (IN) demonstrated safety, immunogenicity, and protection against challenge with numerous matched and unmatched influenza A viruses^11^. Interestingly, IM vaccination elicited strong pulmonary cytotoxic CD8 T cell and serum IgG responses against HA, HA-stalk, and NA, while IN BPL-1357 elicited better pulmonary cytotoxic NK cell, pulmonary CD4 T cell, and mucosal IgA responses. Despite the differences in immunological responses between routes, animals in both IM and IN groups had similarly robust protection^11^.

To further evaluate BPL-1357 in humans, a randomized, double-blind, placebo-controlled phase 1 clinical trial was performed to determine the safety and immunogenicity of the vaccine delivered either IM or IN at an initial dose of approximately 100 μg of total protein per strain (corresponding to an estimated 15 μg of HA per strain).

## Results

### Participants

**Fig. 1:**
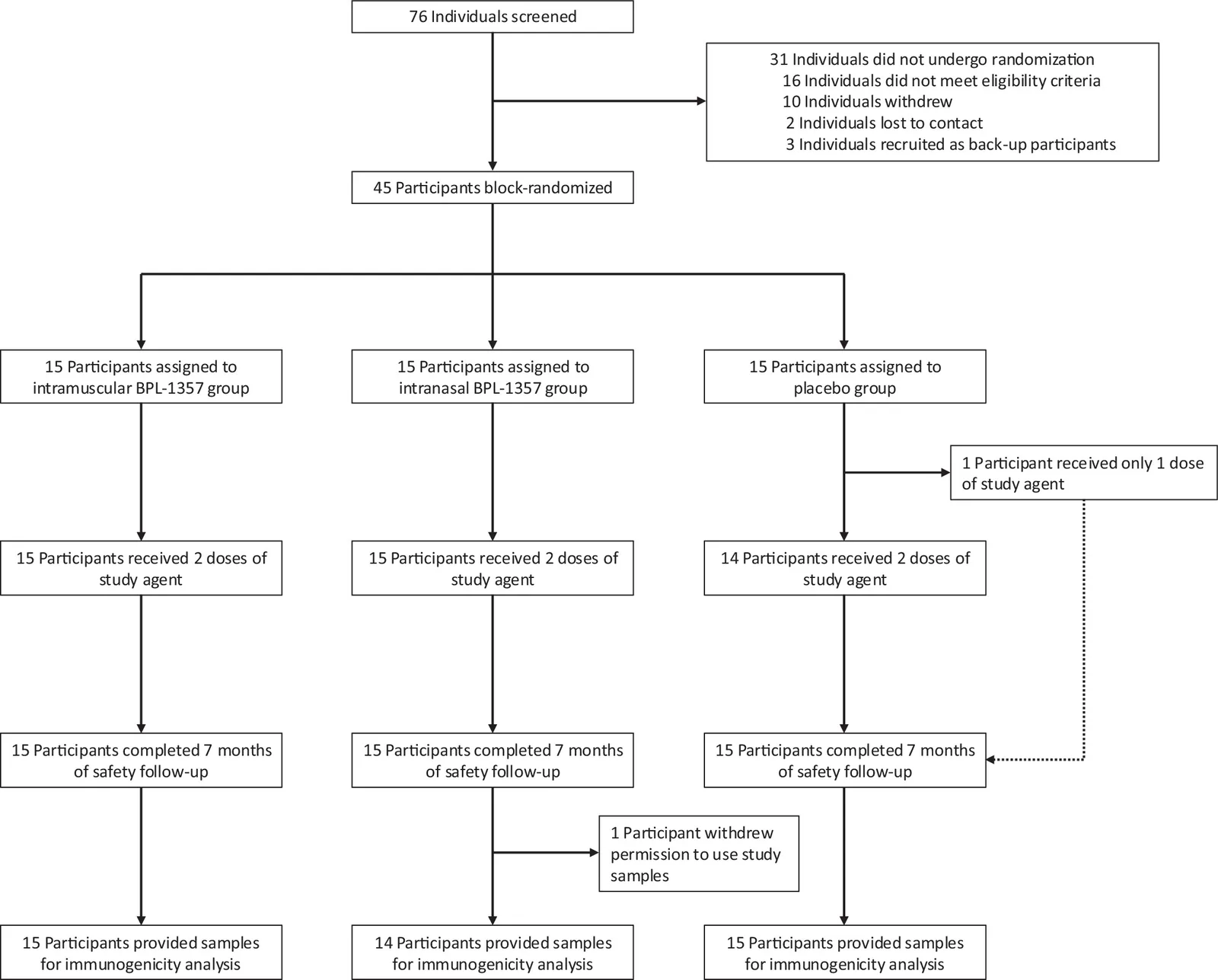
Trial profile. The safety analyses were performed on all participants who received at least one dose of the vaccine. The immunogenicity analyses were performed on all participants who received one dose of vaccine, except one participant in the intranasal group, who withdrew permission to use study samples. One participant in the placebo group received only one dose of the study agent because they missed the second vaccination visit and were unable to reschedule.

Seventy-eight participants were screened between May 2022 and October 2022 (Fig. 1). Of these, forty-five participants were enrolled independent of pre-existing influenza immunity and randomized to receive IM BPL-1357 and IN placebo (*n* = 15), IM placebo and IN BPL-1357 (*n* = 15), or IM placebo and IN placebo (*n* = 15). Both IM and IN vaccines were administered at a single dose level consisting of approximately 15 μg of HA from each subtype (Table S1), in a total volume of 1 ml. Participants were vaccinated twice, 28 days apart (prime and boost). The study was performed between June 2022 and June 2023. Unblinding of the research team and participants occurred after the last participant completed all study follow-up visits. Assays were completed by blinded technicians. One participant in the placebo group received their booster dose late due to infection with severe acute respiratory syndrome coronavirus 2 (SARS-CoV-2). Another participant in the placebo group did not receive the booster dose due to infection with SARS-CoV-2 and subsequent schedule conflicts. One participant in the IN group withdrew permission for sample use after study completion.

Demographic characteristics are detailed in Table 1 and were generally similar across the study groups except for a larger proportion of female participants in the IM group compared to the IN and placebo group.

**Table 1:** Baseline demographics

	Intramuscular Group (*n* = 15)	Intranasal Group (*n* = 15)	Placebo Group (*n* = 15)
Sex			
Female	12 (80)	7 (46.7)	8 (53.3)
Male	3 (20)	8 (53.3)	7 (46.7)
Age	34 ± 8.4	30.3 ± 10.2	27.5 ± 6.0
Race			
Asian	4 (26.7)	5 (33.3)	2 (13.3)
Black	0	2 (13.3)	3 (20)
Multiple	0	1 (6.7)	1 (6.7)
White	10 (66.7)	7 (46.7)	9 (60)
Unknown	1 (6.7)	0	0
Ethnicity			
Hispanic	2 (13.3)	1 (6.7)	0
Not Hispanic	13 (86.7)	14 (93.3)	15 (100)
BMI	24.1 ± 4	24.1 ± 2.9	24.9 ± 4.4

### Safety

**Fig. 2:**
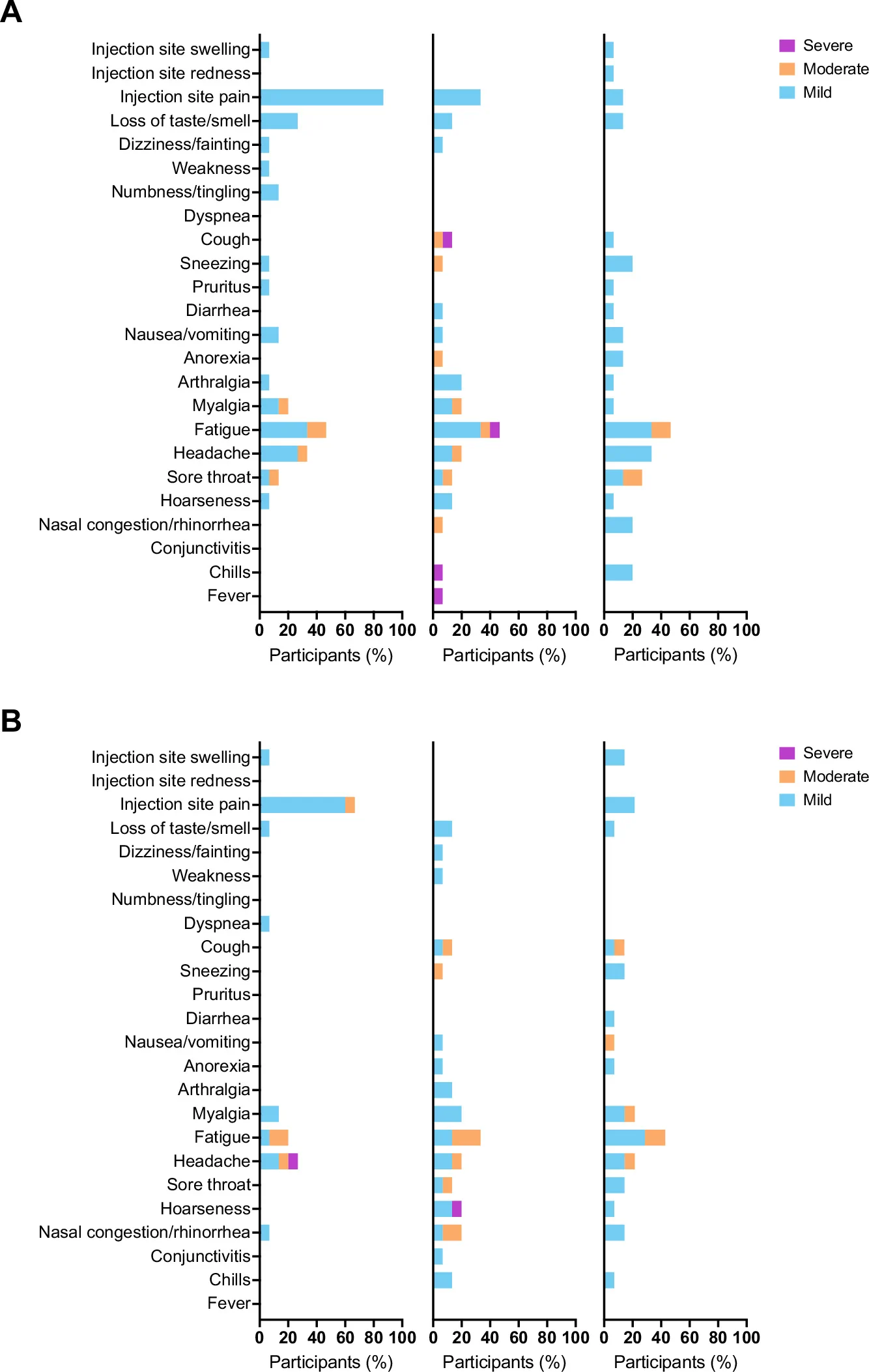
Local and systemic reactogenicity after prime and booster vaccinations. Self-report of local and systemic symptoms and their severity collected through daily survey for 7 days after prime (**A**) and booster (**B**) vaccination for all 45 participants.

No serious adverse events were observed in 6 months of follow-up after the booster vaccination. No prespecified trial pausing or halting rules were met. The frequency and severity of solicited adverse events were comparable across study groups, except for a higher proportion of injection site pain in the IM group (Fig. 2). Injection site pain occurred in 87%, 33%, and 13% of the IM, IN, and placebo groups after the prime vaccine dose, and in 67%, 0%, and 21% of the IM, IN, and placebo groups after the boost, respectively. Twenty-eight days after the booster vaccine dose, a significantly higher proportion of participants in the IM group had intervention-related injection site pain (Tables S2 and S3). The proportions of other adverse events across study groups were not different 28 and 182 days after the booster dose (Tables S2 and S4). Adverse events were generally mild or moderate, with observed severe adverse events during the 210-day study period occurring at frequencies of 3–10% in both vaccine and placebo groups. Severe adverse events were almost all considered unrelated to the vaccine, with the exception of a grade 3 fever in the IN group that was considered possibly related to the study agent. No cases of influenza were detected during the study. Additional information on adverse events (Tables S5 and S6) is available in the Supplementary Appendix.

### Serum hemagglutinin and neuraminidase antibody titers

**Fig. 3:**
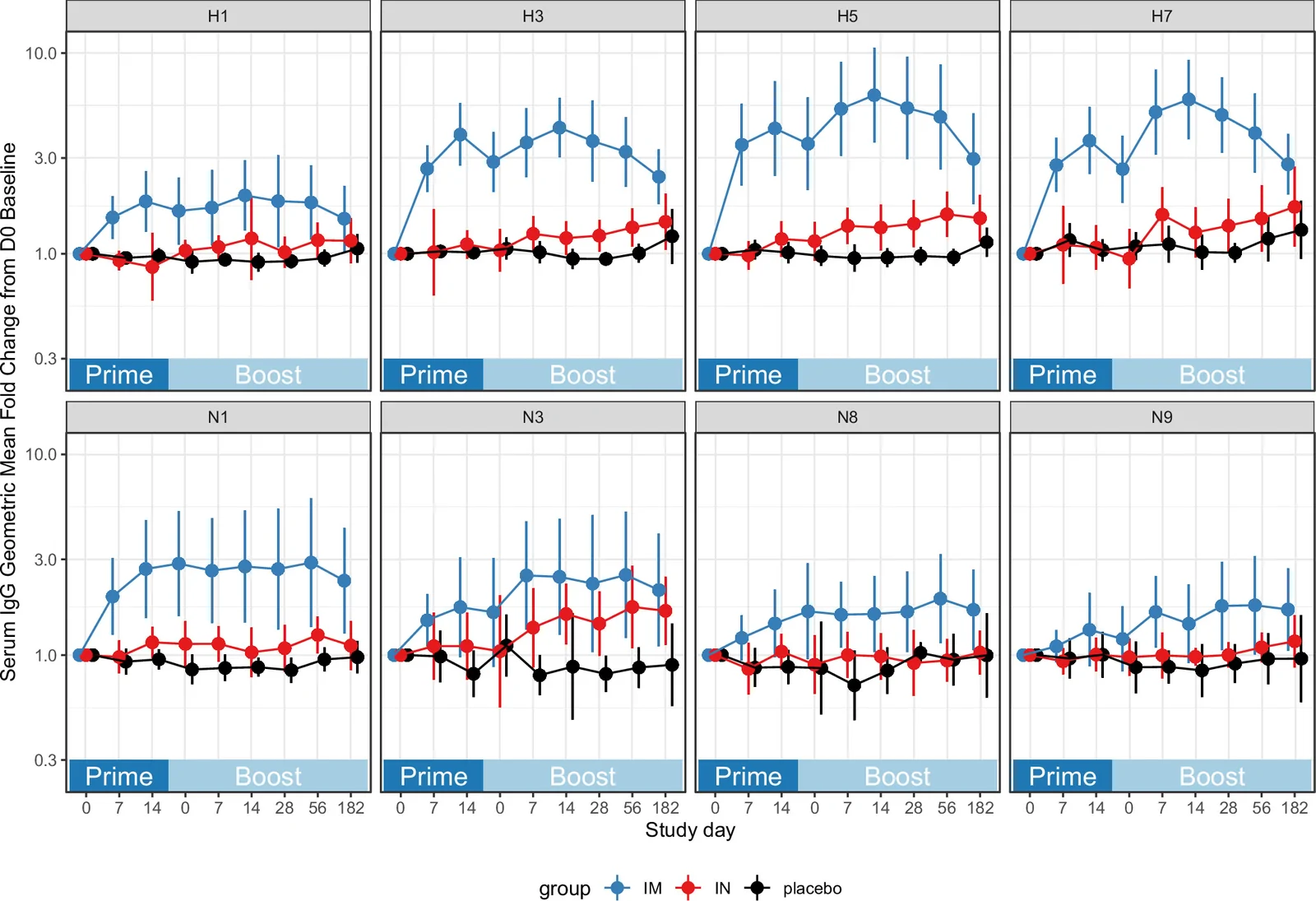
Serum IgG titers against hemagglutinin and neuraminidase antigens after IM and IN BPL-1357 standardized to individual baselines. Serum IgG geometric mean titers (with 95% confidence intervals) measured by enzyme-linked immunosorbent assay to vaccine HA (H1, H3, H5, H7) and NA (N1, N3, N8, N9) antigens are shown as fold change in half-maximal effective concentration EC_50_ values from baseline for participants who received intramuscular (IM) BPL-1357 (*n* = 15), intranasal (IN) BPL-1357 (*n* = 14), and placebo (*n* = 15). Titers are shown across all timepoints, including at baseline (prime study day 0), after the prime vaccination (prime study days 7 and 14), prior to the booster vaccination (boost study day 0, which occurs 28 days after the prime dose), and after the booster vaccination (boost study days 7, 14, 28, 56, and 182).

Antibody binding titers were measured by enzyme-linked immunosorbent assay (ELISA) in serum to all four HA vaccine antigens (H1, H3, H5, H7) and all four NA antigens (N1, N3, N8, N9) at baseline and all subsequent timepoints. Baseline serum IgG geometric mean titers (GMT) against most antigens were comparable across groups, with some exceptions such as N1 and N8 (Table 2; Figs. S1 and S2). Baseline HA serum GMTs were highest against H1 and lowest against H5 and H7. Serum IgG GMTs at 28 days after the booster vaccination were higher against H3, H5, H7, and N1 in the IM group relative to the placebo group (Table 2; Figs. 3, and S1; S2). IN BPL-1357 did not elicit significantly higher serum IgG GMTs relative to placebo. Additional information on the number of samples available at each timepoint is available in the Supplementary Appendix (Table S7).

**Table 2:** Systemic immunogenicity of BPL-1357 vaccine given intramuscularly or intranasally versus placebo

	Baseline Geometric Mean Titers* (95% confidence interval)			Geometric Mean Titers 28 Days After Booster Vaccine Dose* (95% confidence interval)			T-Test		
	IM	IN	Placebo	IM	IN	Placebo	IM vs Placebo	IN vs Placebo	IM vs IN
	*n* = 15	*n* = 14	*n* = 15	*n* = 14	*n* = 14	*n* = 15			
ELISA Anti-Hemagglutinin IgG									
H1	1642.0 (774.1, 3483.1)	2055.2 (1195.0, 3534.4)	1933.8 (1299.6, 2877.6)	2765.3 (1580.3, 4838.7)	2092.6 (1309.2, 3344.8)	1776.1 (1222.7, 2579.8)	0.169	0.561	0.417
H3	312.0 (195.9, 496.9)	307.7 (161.7, 585.6)	353.3 (241.5, 517.0)	1103.8 (737.3, 1652.6)	378.9 (202.4, 709.6)	332.8 (219.1, 505.4)	<0.001	0.714	0.005
H5	194.6 (105.3, 359.6)	258.4 (171.2, 390.2)	291.9 (203.1, 419.7)	1010.1 (631.8, 1615.0)	365.1 (234.4, 568.8)	284.5 (209.7, 386.0)	<0.001	0.328	0.002
H7	137.0 (81.5, 230.3)	122.7 (60.2, 250.1)	139.7 (85.5, 228.3)	638.3 (451.9, 901.6)	169.3 (92.0, 311.3)	141.2 (86.5, 230.8)	<0.001	0.623	0.001
ELISA Anti-Neuraminidase IgG									
N9	13.8 (9.0, 21.3)	12.4 (9.4, 16.3)	14.4 (10.1, 20.7)	24.8 (13.4, 46.0)	12.4 (9.6, 16.1)	13.1 (10.3, 16.7)	0.052	0.744	0.038
N8	14.9 (9.8, 22.7)	16.5 (11.7, 23.2)	22.7 (12.6, 40.8)	25.2 (14.6, 43.6)	15.1 (10.5, 21.7)	23.3 (13.4, 40.6)	0.828	0.171	0.104
N1	78.3 (37.3, 164.5)	245.7 (120.2, 502.3)	335.4 (212.0, 530.6)	222.3 (119.1, 415.0)	265.7 (137.7, 513.0)	281.7 (183.7, 432.1)	0.506	0.874	0.674
N3	18.3 (9.4, 36.0)	20.8 (12.6, 34.3)	24.6 (15.7, 38.6)	43.5 (18.8, 100.5)	29.8 (17.1, 52.1)	19.8 (13.4, 29.4)	0.083	0.212	0.426

*Titers reflect half-maximal effective concentration (EC_50_) values.

Two-tailed t-tests performed on log2-transformed titers (Bonferroni corrected significance threshold for 3 comparisons: *p* < 0.0167).

ELISA denotes enzyme-linked immunosorbent assay, *IM* intramuscular, *IN* intranasal, *H* hemagglutinin, *N* neuraminidase.

### Nasal hemagglutinin and neuraminidase antibody signal

**Fig. 4:**
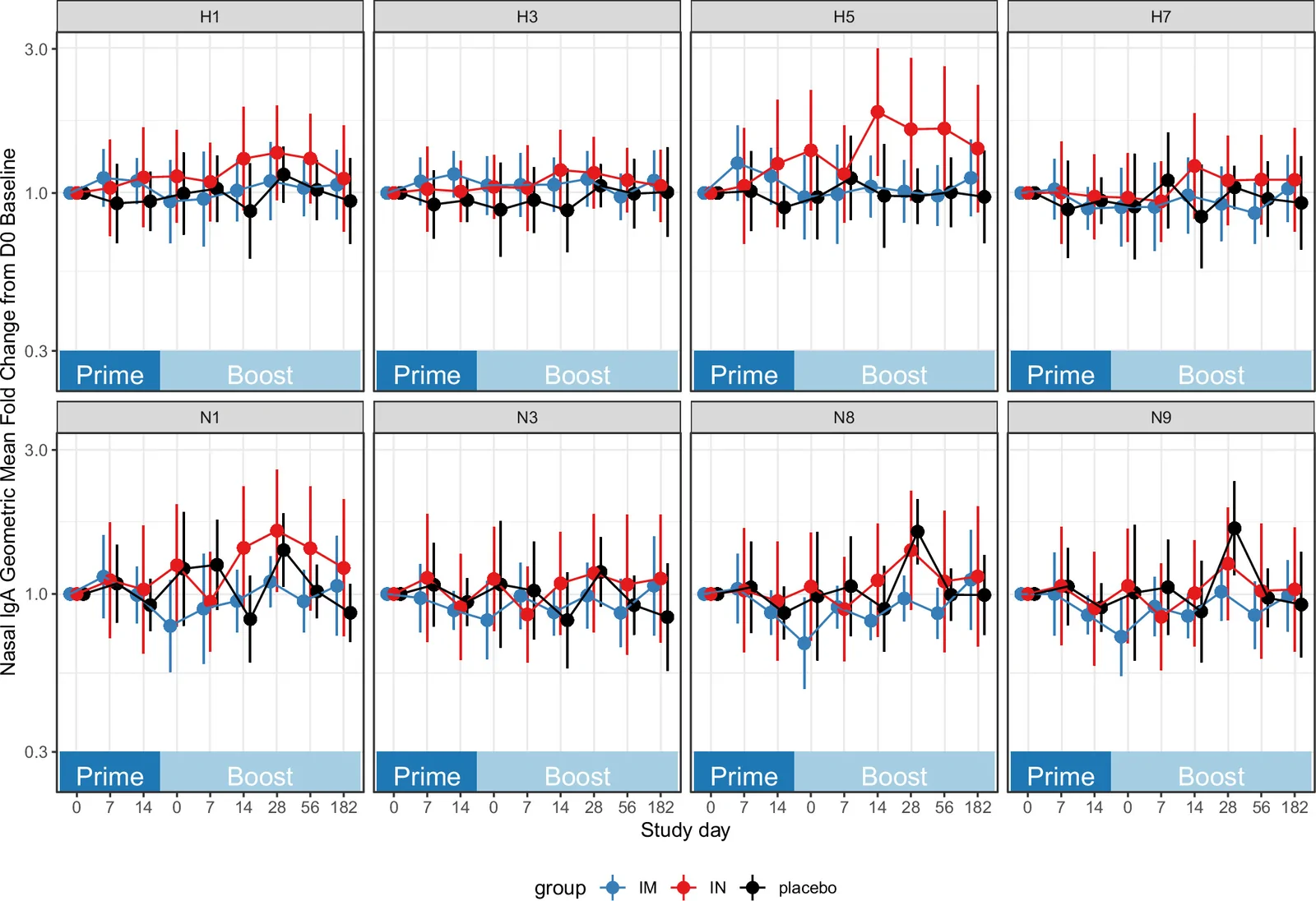
Nasal IgA signal against hemagglutinin and neuraminidase antigens after IM and IN BPL-1357 standardized to individual baselines. Nasal IgA geometric mean signal (with 95% confidence intervals measured by enzyme-linked immunosorbent assay to vaccine HA (H1, H3, H5, H7) and NA (N1, N3, N8, N9) antigens are shown as fold change in optical density values from baseline for participants who received intramuscular (IM) BPL-1357 (*n* = 15), intranasal (IN) BPL-1357 (*n* = 14), and placebo (*n* = 15). Fold change antibody levels are shown across all timepoints, including at baseline (prime study day 0), after the prime vaccination (prime study days 7 and 14), prior to the booster vaccination (boost study day 0, which occurs 28 days after the prime dose), and after the booster vaccination (boost study days 7, 14, 28, 56, and 182).

Antibody binding levels were also measured by ELISA in nasal secretions collected using synthetic absorptive matrix strips against all vaccine HA and NA antigens. Nasal IgA signal against all HA and NA antigens was comparable at baseline across groups. Twenty-eight days after the boost, IgA signal was not significantly different between groups (Table 3 and Figs. 4; S3, S4).

**Table 3:** Mucosal immunogenicity of BPL-1357 vaccine given intramuscularly or intranasally versus placebo

	Baseline Geometric Mean Signal* (95% confidence interval)			Geometric Mean Signal 28 Days After Booster Vaccine Dose* (95% confidence interval)			T-Test		
	IM	IN	Placebo	IM	IN	Placebo	IM vs Placebo	IN vs Placebo	IM vs IN
	*n* = 15	*n* = 14	*n* = 15	*n *= 14	*n* = 14	*n* = 15			
ELISA Anti-Hemagglutinin IgA									
H1	0.386 (0.283, 0.527)	0.409 (0.304, 0.550)	0.438 (0.326, 0.588)	0.446 (0.288, 0.690)	0.556 (0.411, 0.754)	0.504 (0.375, 0.676)	0.621	0.618	0.377
H3	0.454 (0.379, 0.544)	0.483 (0.392, 0.595)	0.514 (0.379, 0.697)	0.525 (0.420, 0.655)	0.564 (0.438, 0.726)	0.543 (0.418, 0.706)	0.832	0.825	0.647
H5	0.203 (0.143, 0.286)	0.232 (0.168, 0.321)	0.271 (0.170, 0.432)	0.213 (0.132, 0.343)	0.377 (0.242, 0.589)	0.264 (0.168, 0.415)	0.487	0.237	0.069
H7	0.290 (0.219, 0.384)	0.324 (0.260, 0.404)	0.315 (0.228, 0.437)	0.279 (0.185, 0.421)	0.357 (0.285, 0.446)	0.329 (0.236, 0.460)	0.505	0.676	0.271
ELISA Anti-Neuraminidase IgA									
N9	0.119 (0.089, 0.158)	0.130 (0.092, 0.185)	0.145 (0.095, 0.223)	0.124 (0.095, 0.162)	0.164 (0.107, 0.252)	0.240 (0.152, 0.379)	0.013	0.201	0.240
N8	0.138 (0.102, 0.186)	0.170 (0.122, 0.238)	0.179 (0.119, 0.269)	0.139 (0.102, 0.190)	0.238 (0.165, 0.343)	0.288 (0.178, 0.464)	0.012	0.504	0.023
N1	0.164 (0.117, 0.229)	0.250 (0.172, 0.363)	0.220 (0.123, 0.393)	0.171 (0.111, 0.264)	0.405 (0.264, 0.622)	0.308 (0.169, 0.561)	0.101	0.433	0.005
N3	0.092 (0.069, 0.123)	0.112 (0.083, 0.152)	0.120 (0.074, 0.194)	0.091 (0.076, 0.110)	0.132 (0.091, 0.190)	0.142 (0.089, 0.226)	0.079	0.792	0.072

*Signal reflects absorbance (optical density at 450 nm) values.

Two-tailed t-tests performed on log2-transformed titers (Bonferroni corrected significance threshold for 3 comparisons: *p* < 0.0167).

ELISA denotes enzyme-linked immunosorbent assay, *IM* intramuscular, *IN* intranasal, *H* hemagglutinin, *N* neuraminidase.

### Serologic and mucosal response rates to hemagglutinin and neuraminidase antigens

A majority of IM BPL-1357 vaccinated participants had a serum IgG response (defined as a 2.5-fold or higher increase in half-maximal effective concentration (EC_50_) titers (serum) or single-point OD_450_ value (nasal) from baseline to 28 days after the booster vaccine) to H3 (71%), H5 (71%), and H7 (86%) but not H1 (29%) (Table 4). Serum IgG responses to N9 (29%), N8 (50%), N1 (21%), and N3 (43%) in the IM group were modest. A minority of participants in the IN group had serologic responses to HA and NA antigens. In the placebo group, no participants had serum IgG responses to any vaccine antigens. No IM BPL-1357 vaccinated participants had a nasal IgA response to any HA or NA antigens. A small number of participants in the IN group had a nasal response to H1 (21%) and H5 (36%) as well as N1 (29%), N3 (14%), N8 (21%), and N9 (14%), though some nasal IgA responses were also seen in the placebo group.

**Table 4:** Serum and nasal response rates to BPL-1357 vaccine given intramuscularly or intranasally versus placebo

	Serum Anti-HA IgG Response percent (95% confidence interval)			Nasal Anti-HA IgA Response percent (95% confidence interval)				Serum Anti-NA IgG Response percent (95% confidence interval)			Nasal Anti-NA IgA Response percent (95% confidence interval)		
	IM	IN	Placebo	IM	IN	Placebo		IM	IN	Placebo	IM	IN	Placebo
	*n* = 14	*n* = 14	*n* = 15	*n* = 14	*n* = 14	*n* = 15		*n* = 14	*n* = 14	*n* = 15	*n* = 14	*n* = 14	n = 15
H1	28.6 (8.4, 58.1)	7.1 (0.2, 33.9)	0.0 (0.0, 21.8)	0.0 (0.0, 23.2)	21.4 (4.7, 50.8)	6.7 (0.2, 31.9)	N9	21.4 (4.7, 50.8)	0.0 (0.0, 23.2)	0.0 (0.0, 21.8)	0.0 (0.0, 23.2)	14.3 (1.8, 42.8)	33.3 (11.8, 61.6)
H3	71.4 (41.9, 91.6)	0.0 (0.0, 23.2)	0.0 (0.0, 21.8)	0.0 (0.0, 23.2)	0.0 (0.0, 23.2)	0.0 (0.0, 21.8)	N8	28.6 (8.4, 58.1)	7.1 (0.2, 33.9)	0.0 (0.0, 21.8)	0.0 (0.0, 23.2)	21.4 (4.7, 50.8)	13.3 (1.7, 40.5)
H5	71.4 (41.9, 91.6)	14.3 (1.8, 42.8)	0.0 (0.0, 21.8)	0.0 (0.0, 23.2)	35.7 (12.8, 64.9)	0.0 (0.0, 21.8)	N1	50.0 (23.0, 77.0)	0.0 (0.0, 23.2)	0.0 (0.0, 21.8)	0.0 (0.0, 23.2)	28.6 (8.4, 58.1)	13.3 (1.7, 40.5)
H7	85.7 (57.2, 98.2)	21.4 (4.7, 50.8)	0.0 (0.0, 21.8)	0.0 (0.0, 23.2)	0.0 (0.0, 23.2)	0.0 (0.0, 21.8)	N3	42.9 (17.7, 71.1)	21.4 (4.7, 50.8)	0.0 (0.0, 21.8)	0.0 (0.0, 23.2)	14.3 (1.8, 42.8)	6.7 (0.2, 31.9)
Any^a^	100.0 (76.8, 100.0)	28.6 (8.4, 58.1)	0.0 (0.0, 21.8)	0.0 (0.0, 23.2)	35.7 (12.8, 64.9)	6.7 (0.2, 31.9)	Any^b^	78.6 (49.2, 95.3)	21.4 (4.7, 50.8)	0.0 (0.0, 21.8)	0.0 (0.0, 23.2)	35.7 (12.8, 64.9)	46.7 (21.3, 73.4)

*IM* denotes intramuscular, *IN* intranasal, *HA* hemagglutinin, *NA* neuraminidase.

A 2.5-fold or greater increase in EC_50_ titer (serum) or single-point OD_450_ value (nasal) 28 days after the booster vaccine dose relative to baseline is considered a response.

^a^Composite of response to any of four HA antigens.

^b^Composite of response to any of four NA antigens.

### Functional and neutralizing antibody responses

HA inhibition (HAI) titers were low at baseline against H3, H5, and H7 and responded minimally across the three groups to all antigens (Table 5). NA inhibition (NAI) titers were higher at baseline and had higher rates of response post-vaccination, with 21%, 14%, 50%, and 64% responding to N9, N8, N1, and N3, respectively. In the IN group, 0%, 8%, 29%, and 50% responded to N9, N8, N1, and N3, respectively, though some responses were also seen in the placebo group. NAI titers were significantly higher in the IM group relative to the IN group and placebo only against the N3 component.

**Table 5:** Serum hemagglutinin and neuraminidase inhibition titers and response to BPL-1357 vaccine given intramuscularly or intranasally versus placebo

	Baseline Titers (95% confidence interval)			Titers 28 Days After Booster Vaccine Dose (95% confidence interval)			T-Test			Responders^a^ percent (95% confidence interval)		
	IM	IN	Placebo	IM	IN	Placebo	IM vs Placebo	IN vs Placebo	IM vs IN	IM	IN	Placebo
	*n* = 15	*n* = 14	*n* = 15	*n* = 14	*n* = 14	*n* = 15				*n* = 14	*n* = 14	*n* = 15
HAI												
H1N9	10.0 (2.8, 35.9)	29.8 (8.8, 100.6)	11.0 (3.3, 37.0)	33.9 (13.3, 86.8)	35.1 (11.8, 104.2)	20.3 (8.1, 51.0)	0.409	0.417	0.960	42.9 (17.7, 71.1)	7.1 (0.2, 33.9)	26.7 (7.8, 55.1)
H3N8	2.2 (1.2, 4.0)	1.8 (0.9, 3.7)	1.0 (NaN, NaN)	3.1 (1.4, 7.0)	1.5 (0.8, 2.9)	1.4 (0.9, 2.1)	0.068	0.744	0.154	21.4 (4.7, 50.8)	0.0 (0.0, 23.2)	13.3 (1.7, 40.5)
H5N1	1.6 (0.9, 2.7)	1.2 (0.8, 1.7)	1.2 (0.8, 1.6)	6.0 (2.8, 12.7)	4.1 (2.0, 8.7)	2.8 (1.3, 5.7)	0.118	0.413	0.447	57.1 (28.9, 82.3)	50.0 (23.0, 77.0)	33.3 (11.8, 61.6)
H7N3	1.6 (0.9, 2.7)	1.0 (NaN, NaN)	1.0 (NaN, NaN)	1.4 (0.9, 2.3)	1.4 (0.9, 2.3)	1.2 (0.8, 1.6)	0.524	0.524	1.000	14.3 (1.8, 42.8)	14.3 (1.8, 42.8)	6.7 (0.2, 31.9)
NAI												
H6N9	42.0 (20.7, 85.5)	40.0 (22.2, 72.0)	43.9 (23.4, 82.4)	84.1 (42.8, 165.0)	44.2 (27.0, 72.3)	48.1 (24.2, 95.7)	0.223	0.829	0.109	7.1 (0.2, 33.9)	0.0 (0.0, 23.2)	0.0 (0.0, 21.8)
H6N8	76.1 (40.3, 143.9)	44.2 (25.2, 77.5)	66.5 (32.0, 138.3)	137.9 (79.7, 238.5)	59.4 (36.4, 97.0)	83.8 (38.1, 184.2)	0.275	0.434	0.020	0.0 (0.0, 23.2)	0.0 (0.0, 23.2)	0.0 (0.0, 21.8)
H6N1	401.5 (170.8, 944.2)	672.5 (309.4, 1461.8)	735.2 (389.3, 1388.3)	1159.3 (620.5, 2166.1)	1103.3 (624.0, 1950.7)	1015.9 (594.2, 1737.0)	0.733	0.822	0.900	14.3 (1.8, 42.8)	7.1 (0.2, 33.9)	13.3 (1.7, 40.5)
H6N3	76.1 (36.8, 157.6)	72.5 (41.3, 127.2)	87.7 (46.7, 164.8)	275.8 (129.5, 587.3)	160.0 (87.1, 293.9)	80.0 (42.5, 150.6)	0.012	0.101	0.237	35.7 (12.8, 64.9)	14.3 (1.8, 42.8)	6.7 (0.2, 31.9)

*HAI* denotes hemagglutination inhibition, *NAI* neuraminidase inhibition.

Two-tailed t-tests performed on log2-transformed titers (Bonferroni corrected significance threshold for 3 comparisons: *p* < 0.0167).

^a^Proportion of participants who experienced a 4-fold or greater increase in titer 28 days after the booster vaccine dose relative to baseline.

Serum microneutralization titers at baseline were low across participants against H3N8, H5N1, and H7N3 but not H1N9 (Table 6). Geometric mean microneutralization titers were higher in the IM group 28 days after the booster vaccination against H3N8, H5N1, and H7N3 (but not H1N9) compared to the placebo and IN groups. Neutralization titers increased against H3N8, H5N1, and H7N3 in the IM vaccinated group, with 71%, 79%, and 29% of participants experiencing a four-fold or greater increase in titers, respectively. A few participants in the IN group and a single participant in the placebo group responded, mostly to H1N9.

**Table 6:** Serum geometric mean microneutralization titers and response to BPL-1357 vaccine given intramuscularly or intranasally versus placebo

	Baseline Geometric Mean Microneutralization Titers^a^ (95% confidence interval)			Geometric Mean Microneutralization Titers 28 Days After Booster Vaccine Dose^a^ (95% confidence interval)			T-Test			Responders^b^ percent (95% confidence interval)		
	IM	IN	Placebo	IM	IN	Placebo	IM vs Placebo	IN vs Placebo	IM vs IN	IM	IN	Placebo
	*n* = 15	*n* = 14	*n* = 15	*n* = 14	*n* = 14	*n* = 15				*n* = 14	*n* = 14	*n* = 15
H1N9	26.4 (7.1, 97.8)	52.6 (16.7, 165.3)	57.0 (20.9, 155.3)	64.6 (21.7, 192.3)	76.1 (36.8, 157.6)	48.9 (16.6, 143.7)	0.700	0.472	0.789	21.4 (4.7, 50.8)	14.3 (1.8, 42.8)	0.0 (0.0, 21.8)
H3N8	1.0 (NaN, NaN)	1. 5 (0.6, 3.7)	1.2 (0.8, 1.9)	9.2 (3.8, 22.2)	1.4 (0.7, 2.7)	1.2 (0.8, 1.6)	<0.001	0.652	0.001	71.4 (41.9, 91.6)	0.0 (0.0, 23.2)	0.0 (0.0, 21.8)
H5N1	1.0 (NaN, NaN)	1.2 (0.8, 1.8)	1.2 (0.8, 1.6)	10.5 (4.4, 25.2)	1.5 (0.8, 2.7)	1.2 (0.8, 1.6)	<0.001	0.432	0.001	78.6 (49.2, 95.3)	7.1 (0.2, 33.9)	0.0 (0.0, 21.8)
H7N3	1.0 (NaN, NaN)	1.0 (NaN, NaN)	1.0 (NaN, NaN)	2.4 (1.0, 5.4)	1.0 (NaN, NaN)	1.0 (NaN, NaN)	0.043	<0.001	0.043	28.6 (8.4, 58.1)	0.0 (0.0, 23.2)	0.0 (0.0, 21.8)

*IM* denotes intramuscular, *IN* intranasal, *NaN* not a number.

Two-tailed t-tests performed on log2-transformed titers (Bonferroni corrected significance threshold for 3 comparisons: *p* < 0.0167).

^a^Samples that did not neutralize were expressed as a level of 1.

^b^Percent of participants who experienced a 4-fold or greater increase in titer 28 days after the booster vaccine dose relative to baseline.

## Discussion

Recent advances in biomedical research have led to the emergence of novel platform technologies and the use of structure-based design, including prefusion-stabilized glycoproteins, to develop vaccines against SARS-CoV-2, respiratory syncytial virus, and influenza^12–17^. Many of these novel strategies can elicit high levels of systemic antibodies against specific, often single antigenic targets. We opted for an approach that would broaden the immune responses as occurs after natural infection. The inclusion of a BPL-inactivated whole-virion affords the immune system an opportunity to develop humoral responses against the entire range of surface glycoproteins as well as cellular responses against internal proteins, which are more highly conserved^18^. Influenza subtypes in this vaccine were selected to include a diverse set of antigens across the phylogenetic trees of both HA and NA to induce a response that in animal models protected against completely heterosubtypic (containing completely different HA and NA from those used in the vaccine) viruses, as well as those of marked public health importance, such as the 1918 pandemic influenza and H5 highly pathogenic avian viruses^11^. We also administered the vaccine intranasally to target the mucosal immune compartment, which may be important in preventing initial disease at the individual level and may have the potential to reduce transmission^19^.

The vaccine, given IM or IN to 30 individuals, was safe and not associated with serious adverse effects in 6 months of follow-up. Most adverse effects were mild or moderate, and the safety profile was relatively similar between vaccine groups and the placebo group, except for a high rate of mild injection site pain in the IM group. These safety findings are consistent with the decades of experience with inactivated influenza vaccines and their record of low reactogenicity^20^. In a previously reported randomized, placebo-controlled trial assessing seasonal trivalent subvirion influenza vaccine in 849 subjects, injection site pain was the only side effect found at a higher frequency in the vaccine group^21^. Similarly, in a study of adjuvanted and unadjuvanted inactivated whole-virus monovalent H5N1 avian influenza vaccine, injection site pain was one of the most common side effects reported^22^. Currently licensed inactivated seasonal influenza vaccines use either split-virion or subunit-virion technology, a strategy that supplanted whole-virion platforms decades ago in an effort to minimize side effect profiles in children, though with potentially detrimental effects to immunogenicity, particularly for non-HA responses^23–26^. Importantly, egg-related impurities in vaccines from this era may have contributed to reactogenicity while purification techniques and quality control testing have since matured. Additionally, avian influenza antigens are known to be poorly immunogenic, and previous vaccine candidates in the last few decades have had success in boosting immunogenicity with the use of adjuvants, higher doses, and/or whole-virus platforms, while maintaining acceptable reactogenicity^22,27–31^.

BPL-1357, given IM without adjuvant, was successful in inducing serum IgG responses against multiple vaccine antigens, but not in inducing nasal IgA responses, similar to the experience with seasonal IM influenza vaccine^32^. It boosted serum binding IgG antibodies against H3, H5, and H7 in most participants, though not against H1. Titers against H1 were already high at baseline in this participant population, likely a manifestation of cross-reactive antibodies from seasonal exposure to influenza A/(H1N1)pdm09 and pre-2009 H1N1 viruses. Consequently, higher baseline antibody titers attenuated the capacity for additional boosting, a phenomenon observed previously with influenza vaccines^33^. Hence, the benefit of this vaccine may be highest in providing protection against novel avian viruses or even drifted H3 strains. Similarly, the benefit of the IM booster dose on serum IgG titers was most evident against H5 and H7. Responses against NA were more modest than HA, a pattern also observed with seasonal influenza vaccines and attributed to the lower abundance of NA on the viral surface, resulting in lower overall NA content in vaccines^5^. Despite the increase in serum binding antibody titers against HA after IM vaccination, serum HAI titers did not increase significantly against most antigens. This may reflect the poor sensitivity of the HAI assay with avian influenza, especially using turkey blood^34^, but could also be caused by antibodies binding away from the receptor binding site. Similarly, differences in responses to NA as measured by ELISA versus NAI assay suggest the possibility of variable responses to vaccine components across participants, with some, like those against N3, more likely to involve the active site than others.

IM BPL-1357 was successful in inducing neutralizing antibody responses in a majority of participants against H3N8 and H5N1. Although the magnitude of post-vaccine neutralization titers was not unexpectedly modest overall, the BPL-1357 vaccine relies on multiple potential immune correlates to provide protection. This phenomenon is evident from the preclinical experience with BPL-1357. In animals, IN BPL-1357 was associated with non-significant increases in serum IgG titers and variable increases in mucosal IgA, but animals were still protected against a wide array of lethal challenges^11^. However, passive serum transfer from IN vaccinated animals to naïve animals was not protective and passive serum transfer from IM vaccinated animals was protective against homologous and partially heterosubtypic challenge (challenge strain contains either an HA or NA of the same subtype as vaccine strains), but not from completely heterosubtypic challenge (challenge strain HA and NA subtypes not found in any vaccine strains)^11^. Hence, the correlates of protection associated with this vaccine are likely to be complex and may include more difficult-to-measure markers stemming from mucosal or cellular immunity. These findings also emphasize that the correlates associated with IM and IN vaccination strategies are likely to be distinct^35^.

Influenza infection occurs predominantly at the level of the upper respiratory mucosa and induces diverse, multifaceted mucosal and systemic responses^32^. Systemic antibodies play a limited role in protecting the upper respiratory tract and may be indirectly correlating with other aspects of immunity^32,36^. Many past and current vaccine strategies have focused on inducing high levels of peripheral blood and serum immune correlates, but this strategy may be neglecting other important effectors of protection. This also may explain why some individuals with high serum titers after vaccination can still be infected with influenza while some unvaccinated individuals with low titers are protected^32,37^. BPL-1357 vaccine contains a wide spectrum of viral antigens for which the humoral and cellular arms of the immune system may respond. Regardless of whether given intranasally or intramuscularly, the BPL-1357 vaccine does not rely on identifying a singular important antigen or particular aspect of the immune system to stimulate. Instead, it affords the immune system and its sophisticated mechanisms access to the entire viral antigenic landscape to determine the most effective response at the individual level.

This study represents the first step in evaluating this novel vaccine in humans. It has several limitations. Due to the small sample size, the power to detect all but the most common adverse effects is low. This study also evaluated a single dose level. Higher dose levels will need to be tested with the goal of maximizing responses across a multitude of immune system aspects while maintaining safety and tolerability. Cellular immune responses, an important component of broad protection in preclinical studies^11^, and Fc-mediated effector functions like antibody-dependent cellular cytotoxicity and antibody-dependent cellular phagocytosis were not measured and will be critical to assess in the future. The thresholds for serological and mucosal response for ELISA titers (≥2.5-fold) and microneutralization titers (≥4-fold) used in this study are arbitrary but are similar to benchmarks used in other studies^38–41^. However, these measures likely have less utility for immunobridging for vaccines that induce multi-target responses and engage multiple aspects of the immune system. Upcoming efforts will also need to focus on demonstrating the benefits of BPL-1357 in preventing disease and then deciphering the comprehensive correlates of protection associated with both routes of the vaccine in humans. Systems biology approaches to mucosal and systemic samples from this and future studies may also shed light on the mechanisms of protection afforded by BPL-1357 and how the responses may differ in humans and animals, with the ultimate goal being to translate the universal protection demonstrated in animal models to broadly protect humans from influenza disease.

## Methods

### Study design and participants

A randomized, placebo-controlled, double-blinded, double-dummy, parallel group study was performed in volunteers 18 to 55 years of age at the National Institutes of Health (NIH) Clinical Center (Bethesda, MD), using a single dose level of vaccine given twice, 28 days apart, either IM or IN. Volunteers were eligible if they were healthy, had no history of severe allergic reactions or allergy to influenza vaccine, and agreed not to receive seasonal influenza vaccination within 8 weeks prior to and 12 weeks after study enrollment. Study participants were enrolled independent of pre-existing influenza immunity. Demographic data, including sex, was self-reported by participants. Participants were reimbursed for their time, up to 1700$. For more details regarding complete enrollment criteria and other aspects of study design, please see the supplement for the full protocol.

The study was approved by the NIH institutional review board and was overseen by an independent data and safety monitoring board. All participants provided written informed consent before enrollment. The study (NCT05027932) was registered (August 28th, 2021) at clinicaltrials.gov and designed and conducted by investigators at the National Institute of Allergy and Infectious Diseases (NIAID) under an Investigational New Drug application (027691) submitted to the Food and Drug Administration (FDA).

### Randomization and blinding

Forty-five participants were block randomized (1:1:1) in groups of 3 to receive IM BPL-1357 and IN placebo (group A), IM placebo and IN BPL-1357 (group B), or IM placebo and IN placebo (group C). The randomization scheme was created by the study statistician using R statistical programing language (version 4.5.1) and conveyed to an unblinded study pharmacist. Blocks of size 9 were used to ensure that within each block, 3 participants were assigned to each of the three groups. Study nurses who administered study agents and obtained vitals, clinicians who obtained assessments and performed physical exams, technicians who performed research assays, and participants were all blinded to treatment group assignment (Fig. 1). Intervention and placebo were similar in appearance.

### Interventions

The BPL-1357 vaccine was developed and characterized in the NIAID intramural research program. It was manufactured by Charles River Laboratories (Wilmington, DE) according to Good Manufacturing Practices. The vaccine contains four low-pathogenicity avian influenza viruses (A/Mallard/Ohio/265/1987 H1N9, A/Pintail/Ohio/339/1987 H3N8, A/Mallard/Maryland/802/2007 H5N1, and A/Environment/Maryland/261/2006 H7N3) grown individually in certified Vero cells and inactivated using β-propiolactone. Inactivated viruses were purified via sucrose gradient and resuspended in phosphate-buffered saline for individual storage in vials. On vaccination day, 0.25 ml of each virus-containing solution was mixed and drawn up, either as 1.0 mL into a single syringe for IM administration or as 1.0 mL divided evenly into two syringes for IN administration. Similarly, placebo was normal saline drawn up either as 1.0 mL into a single syringe for IM administration or as 1.0 mL divided evenly into two syringes for IN administration. Each 1.0 mL contained approximately 100 μg of total protein per strain (range: 90.9 to 133.6 μg), corresponding to approximately 15 μg of HA from each subtype (range: 13.6 to 20 μg). Additional details regarding component concentrations are available in the Supplemental (Table S1).

### Procedures

Participants were vaccinated on day 0 (prime) and day 28 (boost). Follow-up visits occurred 7, 14, and 28 days after each dose, as well as 56 and 182 days after the booster vaccine dose. Participants completed electronic questionnaires for 7 days after each vaccine dose, to document local and systemic reactions as well as medication use to assess adverse events. Clinicians performed assessments and physical examinations, and blood samples were obtained for safety analysis at each visit. Adverse events were graded according to the FDA’s guidance on healthy adult volunteers enrolled in preventive vaccine clinical trials^42^.

Serum and whole blood samples were obtained at each visit to assess immune responses to the vaccine. Nasal samples from both nares were also obtained using nasal synthetic absorptive matrix (SAM) strips (Mucosal Diagnostics, Midhurst, England) at each visit to assess mucosal immune responses.

### Assessment of binding and functional antibody responses

Nasal SAM strips were submerged in elution buffer (phosphate-buffered saline (Quality Biological Solutions, Gaithersburg, MD) with 1% bovine serum albumin (MilliporeSigma, Burlington, MA)) and centrifuged in a spin-filter mini-column, with eluate aliquoted for assessment of antibody responses. Binding antibody responses against H1, H3, H5, H7 and N1, N3, N8, and N9 were assessed via enzyme-linked immunosorbent assay (ELISA) on serum (IgG) and nasal samples (IgA) at each study timepoint. Microplates were coated with Strep-Tactin (Lifesiences GmbH, Göttingen, Germany) in sodium bicarbonate buffer, overnight at 4 °C. Plates were blocked in blocking buffer containing phosphate-buffered saline (PBS), 15% normal goat serum (ThermoFisher, Waltham, MA), 2% whey protein (MilliporeSigma, Burlington, MA), 0.5% Tween-20, and 0.05% sodium azide, for 1 h at room temperature (RT), followed by a 1 h incubation at RT with individually plated C-terminal Strep-tag II-tagged HA antigens and N-terminal Strep-tag II-tagged NA antigens diluted in blocking buffer. Coating concentrations were previously optimized for each antigen. In addition, wells coated with only blocking buffer but no antigen (“no antigen condition”) were processed in parallel to perform sample and dilution-specific background subtraction. Serum was diluted 1:10 in blocking buffer, followed by an 8-step 4-fold serial dilution. SAM specimens were tested at a fixed 1:3 dilution. Diluted specimens were added to each plate and incubated for 1 h at RT. Horseradish peroxidase (HRP)-conjugated goat anti-human IgG secondary antibody (Jackson ImmunoResearch Laboratories, West Grove, PA) and HRP-conjugated goat anti-human IgA (Jackson ImmunoResearch Laboratories, West Grove, PA) were used in blocking buffer without sodium azide and incubated for 1 h at RT, followed by the addition of SureBlue Reserve TMB substrate (LGC Clinical Diagnostics, Milford, MA). Each secondary antibody was used at a previously determined lot-specific optimal concentration. Reactions were stopped using hydrochloric acid. Optical density (OD) at the wavelengths of 450 nm and 650 nm, for antibody quantification and detection of nonspecific OD spikes, respectively, was measured. Antibody titers from serum were reported as half-maximal effective concentrations (EC_50_). For nasal samples, due to low concentrations of antibodies, only a single 1:3 dilution was assessed for each sample, and the signal reflects average absorption readings (optical density at 450 nm). Recombinant NA and HA proteins for ELISA assays were designed using the same sequences as vaccine strain proteins with the addition of Strep-Tag II affinity tag^11,43,44^. They were expressed in insect cells and purified using Strep-Tactin Sepharose or immobilized metal affinity chromatography. Proteins were synthesized at the NIH (Bethesda, MD) and Kemp Proteins (Frederick, MD).

For HAI assays, sera were treated with receptor-destroying enzyme (Denka Seiken, Tokyo, Japan) and incubated overnight in a 37 °C water bath. Sera were serially diluted 2-fold with PBS. Standardized virus was added to the sera and incubated for 15 min at room temperature. The assay virus was identical to the four strains used in the vaccine, except it was not inactivated. Turkey red blood cells (Lampire, Pipersville, Pennsylvania) were added and incubated for 45 min at RT^45^. NAI assays were performed using reassortant viruses with a genetically identical NA to each vaccine virus but a distinct HA subtype (H6)^46^. Reassortant viruses were mixed with serial 2-fold dilutions of heat-inactivated participant sera and incubated overnight at 37 °C in fetuin-coated plates. Plates were washed, and then peanut agglutinin conjugated to horseradish peroxidase was added. After a 2-h incubation in the dark at room temperature, plates were washed, and o-phenylenediamine dihydrochloride was added. After 10 min in the dark at room temperature, the reaction was quenched with sulfuric acid, and the plates were read at 490 nm. To assess microneutralization, serum was serially diluted 2-fold with Dulbecco’s Modified Eagle Medium (Gibco, Waltham, MA) in 96-well plates and incubated with live versions of each of the four vaccine viruses, followed by incubation at 37 °C with Madin-Darby canine kidney (MDCK) cells (American Type Culture Collection, Manassas, VA) for 2-4 days. Neutralization readout was assessed by hemagglutination of 0.5% turkey red blood cells (Lampire, Pipersville, Pennsylvania) in phosphate-buffered saline. HAI assays, NAI assays, and microneutralization titers were assessed at baseline (prime dose day 0, prior to administration of the vaccine) and 28 days after the booster vaccination.

### Statistical analysis

The sample size was calculated based on the probability of detecting safety signals, and the target of 15 participants per treatment group was deemed to give 80% power to detect a 12–14% increase over control rates between 5 and 10%. For the primary safety analysis, a test of proportions was used to compare the combined vaccine group (IM + IN) versus placebo for the proportion of participants experiencing any related adverse events (AEs) of all combined grades through 28 days after the booster vaccination. For the secondary safety analysis, the same test was applied comparing the combined vaccine group versus placebo for related AEs through the final follow-up visit.

For IgG serum titers and IgA mucosal signal, the geometric mean fold-change from baseline was estimated by calculating the mean difference in log_2_-transformed titers between each follow-up timepoint and baseline, then back-transforming to the original scale. To test for pairwise differences in log-transformed titers (28 days after the booster vaccination, or day 56) between IM, IN, and placebo groups, two-tailed t-tests were performed with Bonferroni correction for three comparisons (significance threshold: *p* < 0.0167). Post-hoc response in serum IgG and mucosal IgA signal measured by ELISA was defined as a fold-change ≥2.5 from baseline to day 56, and microneutralization response was defined as a ≥ 4-fold increase from baseline to day 56. 95% confidence intervals on the proportions were estimated using the Clopper-Pearson method.

The safety analyses were performed on all participants who received at least one dose of vaccine, and the immunogenicity analyses were performed on all participants who received one dose of vaccine, except one participant in the intranasal group who withdrew permission to use study samples. One participant from the IM group missed their boost study day 28 visit and was not included in the analyses for that timepoint. Missing data were assumed to be missing completely at random, and no imputation methods were applied. If missing data occurred, all tables and figures indicate the number of participants included in the analysis. No sex or gender-based analyses were performed, considering the small sample size of the study. Data analyses were performed using R statistical programing language (version 4.5.1). Figures were generated using R statistical programming language (version 4.5.1) or GraphPad Prism (version 11.0). The statistical analysis plan is available in the full protocol in the supplement.

### Reporting summary

Further information on research design is available in the Nature Portfolio Reporting Summary linked to this article.

## Supplementary information

Supplementary Information

Reporting Summary

Transparent Peer Review file

## Data Availability

The data generated in this study are provided in the Supplementary Information file.
