# Supplementary material for: Safety and immunogenicity of a BPL-inactivated, whole-virus, universal influenza vaccine: a randomized, double-blinded, double-dummy, placebo-controlled, phase 1 clinical trial
Source: Nat Commun. 2026 Oct 12;17:11539. doi: 10.1038/s41467-026-78583-w (PMC13748664; doi:10.1038/s41467-026-78583-w)
Supplement: Supplementary file 1 [file 41467_2026_78583_MOESM1_ESM.pdf]

Table of Contents

Table S1: Vaccine Component Total Protein Concentrations, Volumes, and Amounts.......... 2

Table S2: Test of proportions on combined vaccine group (IM + IN) vs placebo for proportion of participants with all related AEs of combined grades up to 28 days after the booster vaccine dose 3

Table S3: IM, IN, and placebo groups: proportion of participants with all related AEs of combined grades up to 28 days after the booster vaccine dose 7

Table S4: Test of proportions on combined vaccine group (IM + IN) vs placebo for proportion of participants with all related AEs of combined grades up to the final study visit 11

Table S5: Compare IM, IN and placebo for proportion of participants with all related AEs of combined and individual grades up to the final study visit 15

Table S6: Compare combined vaccine group (IM + IN) vs placebo for proportion of participants with all AEs (including unrelated) of combined and individual grades up to the final study visit 19

Table S7: Number of mucosal and serum samples for each timepoint 28

Figure S1: Serum IgG titers against H1, H3, H5, and H7 after IM and IN BPL-1357 31

Figure S2: Serum IgG titers against N1, N3, N8, and N9 after IM and IN BPL-1357 33

Figure S3: Nasal IgA signal against H1, H3, H5, and H7 after IM and IN BPL-1357.................35

Figure S4: Nasal IgA signal against N1, N3, N8, and N9 after IM and IN BPL-1357.................37

CONSORT 2025 checklist of information to include when reporting a randomised trial.......38

Table S1: Vaccine Component Total Protein Concentrations, Volumes, and Amounts

**Vaccine Component**	**Total Protein Concentration** (μg /mL)	**Volume of Each Component** (mL)	**Total Protein Amount** (μg)	**Estimated Hemagglutinin Amount** (μg)
H1N9	373.1	0.25	93.3	14.0
H3N8	534.4	0.25	133.6	20.0
H5N1	363.6	0.25	90.9	13.6
H7N3	390.6	0.25	97.7	14.6

Table S2: Test of proportions on combined vaccine group (IM + IN) vs placebo for proportion of participants with all related AEs of combined grades up to 28 days after the booster vaccine dose

Severity	Total # AEs	IM + IN (n = 30)	Placebo (n = 15)	p value
Fatigue				
Total	27 (31) 60%	18 (20) 60%	9 (11) 60%	1.000
Mild	20 (22) 44.44%	13 (14) 43.33%	7 (8) 46.67%	NA
Moderate	9 (9) 20%	6 (6) 20%	3 (3) 20%	NA
Injection site pain				
Total	20 (30) 44.44%	20 (30) 66.67%	0 (0) 0%	0.000
Mild	20 (29) 44.44%	20 (29) 66.67%	0 (0) 0%	NA
Moderate	1 (1) 2.22%	1 (1) 3.33%	0 (0) 0%	NA
Headache				
Total	17 (20) 37.78%	11 (13) 36.67%	6 (7) 40%	1.000
Mild	13 (16) 28.89%	8 (10) 26.67%	5 (6) 33.33%	NA
Moderate	4 (4) 8.89%	3 (3) 10%	1 (1) 6.67%	NA
Myalgia				
Total	9 (12) 20%	7 (10) 23.33%	2 (2) 13.33%	0.693
Mild	8 (10) 17.78%	6 (8) 20%	2 (2) 13.33%	NA
Moderate	2 (2) 4.44%	2 (2) 6.67%	0 (0) 0%	NA
Sore throat				
Total	9 (11) 20%	5 (7) 16.67%	4 (4) 26.67%	0.693
Mild	8 (9) 17.78%	4 (5) 13.33%	4 (4) 26.67%	NA
Moderate	2 (2) 4.44%	2 (2) 6.67%	0 (0) 0%	NA
Sneezing				
Total	6 (6) 13.33%	2 (2) 6.67%	4 (4) 26.67%	0.163
Mild	5 (5) 11.11%	1 (1) 3.33%	4 (4) 26.67%	NA
Moderate	1 (1) 2.22%	1 (1) 3.33%	0 (0) 0%	NA
Arthralgia				
Total	5 (6) 11.11%	4 (5) 13.33%	1 (1) 6.67%	0.867
Mild	5 (6) 11.11%	4 (5) 13.33%	1 (1) 6.67%	NA
Hoarseness				
Total	5 (6) 11.11%	4 (5) 13.33%	1 (1) 6.67%	0.867
Mild	5 (6) 11.11%	4 (5) 13.33%	1 (1) 6.67%	NA
Rhinorrhea				
Total	5 (5) 11.11%	3 (3) 10%	2 (2) 13.33%	1.000
Mild	4 (4) 8.89%	2 (2) 6.67%	2 (2) 13.33%	NA
Moderate	1 (1) 2.22%	1 (1) 3.33%	0 (0) 0%	NA
Nasal congestion				
Total	4 (4) 8.89%	1 (1) 3.33%	3 (3) 20%	0.195
Mild	3 (3) 6.67%	0 (0) 0%	3 (3) 20%	NA

Severity	Total # AEs	IM + IN (n = 30)	Placebo (n = 15)	p value
Moderate	1 (1) 2.22%	1 (1) 3.33%	0 (0) 0%	NA
Neutrophil count decreased				
Total	4 (6) 8.89%	2 (2) 6.67%	2 (4) 13.33%	0.853
Mild	2 (2) 4.44%	1 (1) 3.33%	1 (1) 6.67%	NA
Moderate	3 (4) 6.67%	1 (1) 3.33%	2 (3) 13.33%	NA
Chills				
Total	3 (4) 6.67%	0 (0) 0%	3 (4) 20%	0.057
Mild	3 (4) 6.67%	0 (0) 0%	3 (4) 20%	NA
Cough				
Total	3 (3) 6.67%	1 (1) 3.33%	2 (2) 13.33%	0.526
Mild	1 (1) 2.22%	0 (0) 0%	1 (1) 6.67%	NA
Moderate	2 (2) 4.44%	1 (1) 3.33%	1 (1) 6.67%	NA
Hypokalemia				
Total	3 (4) 6.67%	2 (3) 6.67%	1 (1) 6.67%	1.000
Mild	1 (1) 2.22%	1 (1) 3.33%	0 (0) 0%	NA
Moderate	3 (3) 6.67%	2 (2) 6.67%	1 (1) 6.67%	NA
Nausea				
Total	3 (4) 6.67%	2 (2) 6.67%	1 (2) 6.67%	1.000
Mild	3 (4) 6.67%	2 (2) 6.67%	1 (2) 6.67%	NA
Decreased appetite				
Total	2 (2) 4.44%	0 (0) 0%	2 (2) 13.33%	0.201
Mild	2 (2) 4.44%	0 (0) 0%	2 (2) 13.33%	NA
Hemoglobin decreased				
Total	2 (2) 4.44%	1 (1) 3.33%	1 (1) 6.67%	1.000
Mild	2 (2) 4.44%	1 (1) 3.33%	1 (1) 6.67%	NA
Alanine aminotransferase increased				
Total	1 (1) 2.22%	0 (0) 0%	1 (1) 6.67%	0.721
Mild	1 (1) 2.22%	0 (0) 0%	1 (1) 6.67%	NA
Aspartate aminotransferase increased				
Total	1 (1) 2.22%	0 (0) 0%	1 (1) 6.67%	0.721
Mild	1 (1) 2.22%	0 (0) 0%	1 (1) 6.67%	NA
Blood bilirubin increased				
Total	1 (1) 2.22%	0 (0) 0%	1 (1) 6.67%	0.721
Mild	1 (1) 2.22%	0 (0) 0%	1 (1) 6.67%	NA
Diarrhea				
Total	1 (1) 2.22%	1 (1) 3.33%	0 (0) 0%	1.000
Mild	1 (1) 2.22%	1 (1) 3.33%	0 (0) 0%	NA
Dizziness				
Total	1 (1) 2.22%	1 (1) 3.33%	0 (0) 0%	1.000
Mild	1 (1) 2.22%	1 (1) 3.33%	0 (0) 0%	NA

Severity	Total # AEs	IM + IN (n = 30)	Placebo (n = 15)	p value
Erythema				
Total	1 (1) 2.22%	1 (1) 3.33%	0 (0) 0%	1.000
Mild	1 (1) 2.22%	1 (1) 3.33%	0 (0) 0%	NA
Eye discomfort				
Total	1 (1) 2.22%	1 (1) 3.33%	0 (0) 0%	1.000
Mild	1 (1) 2.22%	1 (1) 3.33%	0 (0) 0%	NA
Fever				
Total	1 (1) 2.22%	1 (1) 3.33%	0 (0) 0%	1.000
Severe	1 (1) 2.22%	1 (1) 3.33%	0 (0) 0%	NA
Hyperglycemia				
Total	1 (1) 2.22%	1 (1) 3.33%	0 (0) 0%	1.000
Moderate	1 (1) 2.22%	1 (1) 3.33%	0 (0) 0%	NA
Hypophosphatemia				
Total	1 (1) 2.22%	1 (1) 3.33%	0 (0) 0%	1.000
Mild	1 (1) 2.22%	1 (1) 3.33%	0 (0) 0%	NA
Injection site ecchymosis				
Total	1 (1) 2.22%	1 (1) 3.33%	0 (0) 0%	1.000
Mild	1 (1) 2.22%	1 (1) 3.33%	0 (0) 0%	NA
Migraine				
Total	1 (1) 2.22%	1 (1) 3.33%	0 (0) 0%	1.000
Moderate	1 (1) 2.22%	1 (1) 3.33%	0 (0) 0%	NA
Muscle weakness lower limb				
Total	1 (1) 2.22%	1 (1) 3.33%	0 (0) 0%	1.000
Mild	1 (1) 2.22%	1 (1) 3.33%	0 (0) 0%	NA
Numbness				
Total	1 (1) 2.22%	1 (1) 3.33%	0 (0) 0%	1.000
Mild	1 (1) 2.22%	1 (1) 3.33%	0 (0) 0%	NA
Pain				
Total	1 (1) 2.22%	1 (1) 3.33%	0 (0) 0%	1.000
Mild	1 (1) 2.22%	1 (1) 3.33%	0 (0) 0%	NA
Paresthesia				
Total	1 (1) 2.22%	1 (1) 3.33%	0 (0) 0%	1.000
Mild	1 (1) 2.22%	1 (1) 3.33%	0 (0) 0%	NA
Pruritus				
Total	1 (1) 2.22%	1 (1) 3.33%	0 (0) 0%	1.000
Mild	1 (1) 2.22%	1 (1) 3.33%	0 (0) 0%	NA
Vertigo				
Total	1 (1) 2.22%	1 (1) 3.33%	0 (0) 0%	1.000
Mild	1 (1) 2.22%	1 (1) 3.33%	0 (0) 0%	NA

Severity	Total # AEs	IM + IN (n = 30)	Placebo (n = 15)	p value
WBC decreased				
Total	1 (1) 2.22%	1 (1) 3.33%	0 (0) 0%	1.000
Mild	1 (1) 2.22%	1 (1) 3.33%	0 (0) 0%	NA
WBC increased				
Total	1 (1) 2.22%	0 (0) 0%	1 (1) 6.67%	0.721
Mild	1 (1) 2.22%	0 (0) 0%	1 (1) 6.67%	NA

A two-sided test of proportions was performed on AE proportions (significance threshold: $p < 0.05$).

Table S3: IM, IN, and placebo groups: proportion of participants with all related AEs of combined grades up to 28 days after the booster vaccine dose

Severity	Total # AEs	IM (n = 15)	IN (n = 15)	Placebo (n = 15)
Fatigue				
Total	27 (31) 60%	10 (11) 66.67%	8 (9) 53.33%	9 (11) 60%
Mild	20 (22) 44.44%	7 (7) 46.67%	6 (7) 40%	7 (8) 46.67%
Moderate	9 (9) 20%	4 (4) 26.67%	2 (2) 13.33%	3 (3) 20%
Injection site pain				
Total	20 (30) 44.44%	14 (24) 93.33%	6 (6) 40%	0 (0) 0%
Mild	20 (29) 44.44%	14 (23) 93.33%	6 (6) 40%	0 (0) 0%
Moderate	1 (1) 2.22%	1 (1) 6.67%	0 (0) 0%	0 (0) 0%
Headache				
Total	17 (20) 37.78%	8 (10) 53.33%	3 (3) 20%	6 (7) 40%
Mild	13 (16) 28.89%	5 (7) 33.33%	3 (3) 20%	5 (6) 33.33%
Moderate	4 (4) 8.89%	3 (3) 20%	0 (0) 0%	1 (1) 6.67%
Myalgia				
Total	9 (12) 20%	4 (6) 26.67%	3 (4) 20%	2 (2) 13.33%
Mild	8 (10) 17.78%	4 (5) 26.67%	2 (3) 13.33%	2 (2) 13.33%
Moderate	2 (2) 4.44%	1 (1) 6.67%	1 (1) 6.67%	0 (0) 0%
Sore throat				
Total	9 (11) 20%	3 (3) 20%	2 (4) 13.33%	4 (4) 26.67%
Mild	8 (9) 17.78%	2 (2) 13.33%	2 (3) 13.33%	4 (4) 26.67%
Moderate	2 (2) 4.44%	1 (1) 6.67%	1 (1) 6.67%	0 (0) 0%
Sneezing				
Total	6 (6) 13.33%	1 (1) 6.67%	1 (1) 6.67%	4 (4) 26.67%
Mild	5 (5) 11.11%	1 (1) 6.67%	0 (0) 0%	4 (4) 26.67%
Moderate	1 (1) 2.22%	0 (0) 0%	1 (1) 6.67%	0 (0) 0%
Arthralgia				
Total	5 (6) 11.11%	1 (1) 6.67%	3 (4) 20%	1 (1) 6.67%
Mild	5 (6) 11.11%	1 (1) 6.67%	3 (4) 20%	1 (1) 6.67%
Hoarseness				
Total	5 (6) 11.11%	1 (1) 6.67%	3 (4) 20%	1 (1) 6.67%
Mild	5 (6) 11.11%	1 (1) 6.67%	3 (4) 20%	1 (1) 6.67%
Rhinorrhea				
Total	5 (5) 11.11%	1 (1) 6.67%	2 (2) 13.33%	2 (2) 13.33%
Mild	4 (4) 8.89%	1 (1) 6.67%	1 (1) 6.67%	2 (2) 13.33%
Moderate	1 (1) 2.22%	0 (0) 0%	1 (1) 6.67%	0 (0) 0%
Nasal congestion				
Total	4 (4) 8.89%	0 (0) 0%	1 (1) 6.67%	3 (3) 20%
Mild	3 (3) 6.67%	0 (0) 0%	0 (0) 0%	3 (3) 20%
Moderate	1 (1) 2.22%	0 (0) 0%	1 (1) 6.67%	0 (0) 0%

Severity	Total # AEs	IM (n = 15)	IN (n = 15)	Placebo (n = 15)
Neutrophil count decreased				
Total	4 (6) 8.89%	2 (2) 13.33%	0 (0) 0%	2 (4) 13.33%
Mild	2 (2) 4.44%	1 (1) 6.67%	0 (0) 0%	1 (1) 6.67%
Moderate	3 (4) 6.67%	1 (1) 6.67%	0 (0) 0%	2 (3) 13.33%
Chills				
Total	3 (4) 6.67%	0 (0) 0%	0 (0) 0%	3 (4) 20%
Mild	3 (4) 6.67%	0 (0) 0%	0 (0) 0%	3 (4) 20%
Cough				
Total	3 (3) 6.67%	0 (0) 0%	1 (1) 6.67%	2 (2) 13.33%
Mild	1 (1) 2.22%	0 (0) 0%	0 (0) 0%	1 (1) 6.67%
Moderate	2 (2) 4.44%	0 (0) 0%	1 (1) 6.67%	1 (1) 6.67%
Hypokalemia				
Total	3 (4) 6.67%	0 (0) 0%	2 (3) 13.33%	1 (1) 6.67%
Mild	1 (1) 2.22%	0 (0) 0%	1 (1) 6.67%	0 (0) 0%
Moderate	3 (3) 6.67%	0 (0) 0%	2 (2) 13.33%	1 (1) 6.67%
Nausea				
Total	3 (4) 6.67%	1 (1) 6.67%	1 (1) 6.67%	1 (2) 6.67%
Mild	3 (4) 6.67%	1 (1) 6.67%	1 (1) 6.67%	1 (2) 6.67%
Decreased appetite				
Total	2 (2) 4.44%	0 (0) 0%	0 (0) 0%	2 (2) 13.33%
Mild	2 (2) 4.44%	0 (0) 0%	0 (0) 0%	2 (2) 13.33%
Hemoglobin decreased				
Total	2 (2) 4.44%	0 (0) 0%	1 (1) 6.67%	1 (1) 6.67%
Mild	2 (2) 4.44%	0 (0) 0%	1 (1) 6.67%	1 (1) 6.67%
Alanine aminotransferase increased				
Total	1 (1) 2.22%	0 (0) 0%	0 (0) 0%	1 (1) 6.67%
Mild	1 (1) 2.22%	0 (0) 0%	0 (0) 0%	1 (1) 6.67%
Aspartate aminotransferase increased				
Total	1 (1) 2.22%	0 (0) 0%	0 (0) 0%	1 (1) 6.67%
Mild	1 (1) 2.22%	0 (0) 0%	0 (0) 0%	1 (1) 6.67%
Blood bilirubin increased				
Total	1 (1) 2.22%	0 (0) 0%	0 (0) 0%	1 (1) 6.67%
Mild	1 (1) 2.22%	0 (0) 0%	0 (0) 0%	1 (1) 6.67%
Diarrhea				
Total	1 (1) 2.22%	0 (0) 0%	1 (1) 6.67%	0 (0) 0%
Mild	1 (1) 2.22%	0 (0) 0%	1 (1) 6.67%	0 (0) 0%
Dizziness				
Total	1 (1) 2.22%	0 (0) 0%	1 (1) 6.67%	0 (0) 0%
Mild	1 (1) 2.22%	0 (0) 0%	1 (1) 6.67%	0 (0) 0%
Erythema				

Severity	Total # AEs	IM (n = 15)	IN (n = 15)	Placebo (n = 15)
Total	1 (1) 2.22%	0 (0) 0%	1 (1) 6.67%	0 (0) 0%
Mild	1 (1) 2.22%	0 (0) 0%	1 (1) 6.67%	0 (0) 0%
Eye discomfort				
Total	1 (1) 2.22%	0 (0) 0%	1 (1) 6.67%	0 (0) 0%
Mild	1 (1) 2.22%	0 (0) 0%	1 (1) 6.67%	0 (0) 0%
Fever				
Total	1 (1) 2.22%	0 (0) 0%	1 (1) 6.67%	0 (0) 0%
Severe	1 (1) 2.22%	0 (0) 0%	1 (1) 6.67%	0 (0) 0%
Hyperglycemia				
Total	1 (1) 2.22%	1 (1) 6.67%	0 (0) 0%	0 (0) 0%
Moderate	1 (1) 2.22%	1 (1) 6.67%	0 (0) 0%	0 (0) 0%
Hypophosphatemia				
Total	1 (1) 2.22%	1 (1) 6.67%	0 (0) 0%	0 (0) 0%
Mild	1 (1) 2.22%	1 (1) 6.67%	0 (0) 0%	0 (0) 0%
Injection site ecchymosis				
Total	1 (1) 2.22%	1 (1) 6.67%	0 (0) 0%	0 (0) 0%
Mild	1 (1) 2.22%	1 (1) 6.67%	0 (0) 0%	0 (0) 0%
Migraine				
Total	1 (1) 2.22%	1 (1) 6.67%	0 (0) 0%	0 (0) 0%
Moderate	1 (1) 2.22%	1 (1) 6.67%	0 (0) 0%	0 (0) 0%
Muscle weakness lower limb				
Total	1 (1) 2.22%	0 (0) 0%	1 (1) 6.67%	0 (0) 0%
Mild	1 (1) 2.22%	0 (0) 0%	1 (1) 6.67%	0 (0) 0%
Numbness				
Total	1 (1) 2.22%	1 (1) 6.67%	0 (0) 0%	0 (0) 0%
Mild	1 (1) 2.22%	1 (1) 6.67%	0 (0) 0%	0 (0) 0%
Pain				
Total	1 (1) 2.22%	0 (0) 0%	1 (1) 6.67%	0 (0) 0%
Mild	1 (1) 2.22%	0 (0) 0%	1 (1) 6.67%	0 (0) 0%
Paresthesia				
Total	1 (1) 2.22%	1 (1) 6.67%	0 (0) 0%	0 (0) 0%
Mild	1 (1) 2.22%	1 (1) 6.67%	0 (0) 0%	0 (0) 0%
Pruritus				
Total	1 (1) 2.22%	0 (0) 0%	1 (1) 6.67%	0 (0) 0%
Mild	1 (1) 2.22%	0 (0) 0%	1 (1) 6.67%	0 (0) 0%
Vertigo				
Total	1 (1) 2.22%	0 (0) 0%	1 (1) 6.67%	0 (0) 0%
Mild	1 (1) 2.22%	0 (0) 0%	1 (1) 6.67%	0 (0) 0%
WBC decreased				
Total	1 (1) 2.22%	1 (1) 6.67%	0 (0) 0%	0 (0) 0%

Severity	Total # AEs	IM (n = 15)	IN (n = 15)	Placebo (n = 15)
Mild	1 (1) 2.22%	1 (1) 6.67%	0 (0) 0%	0 (0) 0%
WBC increased				
Total	1 (1) 2.22%	0 (0) 0%	0 (0) 0%	1 (1) 6.67%
Mild	1 (1) 2.22%	0 (0) 0%	0 (0) 0%	1 (1) 6.67%

Table S4: Test of proportions on combined vaccine group (IM + IN) vs placebo for proportion of participants with all related AEs of combined grades up to the final study visit

Severity	Total # AEs	IM + IN (n = 30)	Placebo (n = 15)	p value
Fatigue				
Total	27 (31) 60%	18 (20) 60%	9 (11) 60%	1.000
Mild	20 (22) 44.44%	13 (14) 43.33%	7 (8) 46.67%	NA
Moderate	9 (9) 20%	6 (6) 20%	3 (3) 20%	NA
Injection site pain				
Total	20 (30) 44.44%	20 (30) 66.67%	0 (0) 0%	0.000
Mild	20 (29) 44.44%	20 (29) 66.67%	0 (0) 0%	NA
Moderate	1 (1) 2.22%	1 (1) 3.33%	0 (0) 0%	NA
Headache				
Total	17 (20) 37.78%	11 (13) 36.67%	6 (7) 40%	1.000
Mild	13 (16) 28.89%	8 (10) 26.67%	5 (6) 33.33%	NA
Moderate	4 (4) 8.89%	3 (3) 10%	1 (1) 6.67%	NA
Myalgia				
Total	9 (12) 20%	7 (10) 23.33%	2 (2) 13.33%	0.693
Mild	8 (10) 17.78%	6 (8) 20%	2 (2) 13.33%	NA
Moderate	2 (2) 4.44%	2 (2) 6.67%	0 (0) 0%	NA
Sore throat				
Total	9 (11) 20%	5 (7) 16.67%	4 (4) 26.67%	0.693
Mild	8 (9) 17.78%	4 (5) 13.33%	4 (4) 26.67%	NA
Moderate	2 (2) 4.44%	2 (2) 6.67%	0 (0) 0%	NA
Sneezing				
Total	6 (6) 13.33%	2 (2) 6.67%	4 (4) 26.67%	0.163
Mild	5 (5) 11.11%	1 (1) 3.33%	4 (4) 26.67%	NA
Moderate	1 (1) 2.22%	1 (1) 3.33%	0 (0) 0%	NA
Arthralgia				
Total	5 (6) 11.11%	4 (5) 13.33%	1 (1) 6.67%	0.867
Mild	5 (6) 11.11%	4 (5) 13.33%	1 (1) 6.67%	NA
Hoarseness				
Total	5 (6) 11.11%	4 (5) 13.33%	1 (1) 6.67%	0.867
Mild	5 (6) 11.11%	4 (5) 13.33%	1 (1) 6.67%	NA
Rhinorrhea				
Total	5 (5) 11.11%	3 (3) 10%	2 (2) 13.33%	1.000
Mild	4 (4) 8.89%	2 (2) 6.67%	2 (2) 13.33%	NA
Moderate	1 (1) 2.22%	1 (1) 3.33%	0 (0) 0%	NA
Nasal congestion				
Total	4 (4) 8.89%	1 (1) 3.33%	3 (3) 20%	0.195
Mild	3 (3) 6.67%	0 (0) 0%	3 (3) 20%	NA
Moderate	1 (1) 2.22%	1 (1) 3.33%	0 (0) 0%	NA

Severity	Total # AEs	IM + IN (n = 30)	Placebo (n = 15)	p value
Neutrophil count decreased				
Total	4 (6) 8.89%	2 (2) 6.67%	2 (4) 13.33%	0.853
Mild	2 (2) 4.44%	1 (1) 3.33%	1 (1) 6.67%	NA
Moderate	3 (4) 6.67%	1 (1) 3.33%	2 (3) 13.33%	NA
Chills				
Total	3 (4) 6.67%	0 (0) 0%	3 (4) 20%	0.057
Mild	3 (4) 6.67%	0 (0) 0%	3 (4) 20%	NA
Cough				
Total	3 (3) 6.67%	1 (1) 3.33%	2 (2) 13.33%	0.526
Mild	1 (1) 2.22%	0 (0) 0%	1 (1) 6.67%	NA
Moderate	2 (2) 4.44%	1 (1) 3.33%	1 (1) 6.67%	NA
Hypokalemia				
Total	3 (4) 6.67%	2 (3) 6.67%	1 (1) 6.67%	1.000
Mild	1 (1) 2.22%	1 (1) 3.33%	0 (0) 0%	NA
Moderate	3 (3) 6.67%	2 (2) 6.67%	1 (1) 6.67%	NA
Nausea				
Total	3 (4) 6.67%	2 (2) 6.67%	1 (2) 6.67%	1.000
Mild	3 (4) 6.67%	2 (2) 6.67%	1 (2) 6.67%	NA
Decreased appetite				
Total	2 (2) 4.44%	0 (0) 0%	2 (2) 13.33%	0.201
Mild	2 (2) 4.44%	0 (0) 0%	2 (2) 13.33%	NA
Hemoglobin decreased				
Total	2 (2) 4.44%	1 (1) 3.33%	1 (1) 6.67%	1.000
Mild	2 (2) 4.44%	1 (1) 3.33%	1 (1) 6.67%	NA
Alanine aminotransferase increased				
Total	1 (2) 2.22%	0 (0) 0%	1 (2) 6.67%	0.721
Mild	1 (2) 2.22%	0 (0) 0%	1 (2) 6.67%	NA
Aspartate aminotransferase increased				
Total	1 (2) 2.22%	0 (0) 0%	1 (2) 6.67%	0.721
Mild	1 (2) 2.22%	0 (0) 0%	1 (2) 6.67%	NA
Blood bilirubin increased				
Total	1 (1) 2.22%	0 (0) 0%	1 (1) 6.67%	0.721
Mild	1 (1) 2.22%	0 (0) 0%	1 (1) 6.67%	NA
Diarrhea				
Total	1 (1) 2.22%	1 (1) 3.33%	0 (0) 0%	1.000
Mild	1 (1) 2.22%	1 (1) 3.33%	0 (0) 0%	NA
Dizziness				
Total	1 (1) 2.22%	1 (1) 3.33%	0 (0) 0%	1.000
Mild	1 (1) 2.22%	1 (1) 3.33%	0 (0) 0%	NA
Erythema				

Severity	Total # AEs	IM + IN (n = 30)	Placebo (n = 15)	p value
Total	1 (1) 2.22%	1 (1) 3.33%	0 (0) 0%	1.000
Mild	1 (1) 2.22%	1 (1) 3.33%	0 (0) 0%	NA
Eye discomfort				
Total	1 (1) 2.22%	1 (1) 3.33%	0 (0) 0%	1.000
Mild	1 (1) 2.22%	1 (1) 3.33%	0 (0) 0%	NA
Fever				
Total	1 (1) 2.22%	1 (1) 3.33%	0 (0) 0%	1.000
Severe	1 (1) 2.22%	1 (1) 3.33%	0 (0) 0%	NA
Hyperglycemia				
Total	1 (1) 2.22%	1 (1) 3.33%	0 (0) 0%	1.000
Moderate	1 (1) 2.22%	1 (1) 3.33%	0 (0) 0%	NA
Hypophosphatemia				
Total	1 (1) 2.22%	1 (1) 3.33%	0 (0) 0%	1.000
Mild	1 (1) 2.22%	1 (1) 3.33%	0 (0) 0%	NA
Injection site ecchymosis				
Total	1 (1) 2.22%	1 (1) 3.33%	0 (0) 0%	1.000
Mild	1 (1) 2.22%	1 (1) 3.33%	0 (0) 0%	NA
Migraine				
Total	1 (1) 2.22%	1 (1) 3.33%	0 (0) 0%	1.000
Moderate	1 (1) 2.22%	1 (1) 3.33%	0 (0) 0%	NA
Muscle weakness lower limb				
Total	1 (1) 2.22%	1 (1) 3.33%	0 (0) 0%	1.000
Mild	1 (1) 2.22%	1 (1) 3.33%	0 (0) 0%	NA
Numbness				
Total	1 (1) 2.22%	1 (1) 3.33%	0 (0) 0%	1.000
Mild	1 (1) 2.22%	1 (1) 3.33%	0 (0) 0%	NA
Pain				
Total	1 (1) 2.22%	1 (1) 3.33%	0 (0) 0%	1.000
Mild	1 (1) 2.22%	1 (1) 3.33%	0 (0) 0%	NA
Paresthesia				
Total	1 (1) 2.22%	1 (1) 3.33%	0 (0) 0%	1.000
Mild	1 (1) 2.22%	1 (1) 3.33%	0 (0) 0%	NA
Pruritus				
Total	1 (1) 2.22%	1 (1) 3.33%	0 (0) 0%	1.000
Mild	1 (1) 2.22%	1 (1) 3.33%	0 (0) 0%	NA
Vertigo				
Total	1 (1) 2.22%	1 (1) 3.33%	0 (0) 0%	1.000
Mild	1 (1) 2.22%	1 (1) 3.33%	0 (0) 0%	NA
WBC decreased				
Total	1 (1) 2.22%	1 (1) 3.33%	0 (0) 0%	1.000

Severity	Total # AEs	IM + IN (n = 30)	Placebo (n = 15)	p value
Mild	1 (1) 2.22%	1 (1) 3.33%	0 (0) 0%	NA
WBC increased				
Total	1 (1) 2.22%	0 (0) 0%	1 (1) 6.67%	0.721
Mild	1 (1) 2.22%	0 (0) 0%	1 (1) 6.67%	NA

A two-sided test of proportions was performed on AE proportions (significance threshold: $p < 0.05$).

Table S5: Compare IM, IN and placebo for proportion of participants with all related AEs of combined and individual grades up to the final study visit

Severity	IM (n = 15)	IN (n = 15)	Placebo (n = 15)
Fatigue			
Total	10 (11) 66.67%	8 (9) 53.33%	9 (11) 60%
Mild	7 (7) 46.67%	6 (7) 40%	7 (8) 46.67%
Moderate	4 (4) 26.67%	2 (2) 13.33%	3 (3) 20%
Injection site pain			
Total	14 (24) 93.33%	6 (6) 40%	0 (0) 0%
Mild	14 (23) 93.33%	6 (6) 40%	0 (0) 0%
Moderate	1 (1) 6.67%	0 (0) 0%	0 (0) 0%
Headache			
Total	8 (10) 53.33%	3 (3) 20%	6 (7) 40%
Mild	5 (7) 33.33%	3 (3) 20%	5 (6) 33.33%
Moderate	3 (3) 20%	0 (0) 0%	1 (1) 6.67%
Myalgia			
Total	4 (6) 26.67%	3 (4) 20%	2 (2) 13.33%
Mild	4 (5) 26.67%	2 (3) 13.33%	2 (2) 13.33%
Moderate	1 (1) 6.67%	1 (1) 6.67%	0 (0) 0%
Sore throat			
Total	3 (3) 20%	2 (4) 13.33%	4 (4) 26.67%
Mild	2 (2) 13.33%	2 (3) 13.33%	4 (4) 26.67%
Moderate	1 (1) 6.67%	1 (1) 6.67%	0 (0) 0%
Sneezing			
Total	1 (1) 6.67%	1 (1) 6.67%	4 (4) 26.67%
Mild	1 (1) 6.67%	0 (0) 0%	4 (4) 26.67%
Moderate	0 (0) 0%	1 (1) 6.67%	0 (0) 0%
Arthralgia			
Total	1 (1) 6.67%	3 (4) 20%	1 (1) 6.67%
Mild	1 (1) 6.67%	3 (4) 20%	1 (1) 6.67%
Hoarseness			
Total	1 (1) 6.67%	3 (4) 20%	1 (1) 6.67%
Mild	1 (1) 6.67%	3 (4) 20%	1 (1) 6.67%
Rhinorrhea			
Total	1 (1) 6.67%	2 (2) 13.33%	2 (2) 13.33%
Mild	1 (1) 6.67%	1 (1) 6.67%	2 (2) 13.33%
Moderate	0 (0) 0%	1 (1) 6.67%	0 (0) 0%
Nasal congestion			
Total	0 (0) 0%	1 (1) 6.67%	3 (3) 20%
Mild	0 (0) 0%	0 (0) 0%	3 (3) 20%
Moderate	0 (0) 0%	1 (1) 6.67%	0 (0) 0%

Severity	IM (n = 15)	IN (n = 15)	Placebo (n = 15)
Neutrophil count decreased			
Total	2 (2) 13.33%	0 (0) 0%	2 (4) 13.33%
Mild	1 (1) 6.67%	0 (0) 0%	1 (1) 6.67%
Moderate	1 (1) 6.67%	0 (0) 0%	2 (3) 13.33%
Chills			
Total	0 (0) 0%	0 (0) 0%	3 (4) 20%
Mild	0 (0) 0%	0 (0) 0%	3 (4) 20%
Cough			
Total	0 (0) 0%	1 (1) 6.67%	2 (2) 13.33%
Mild	0 (0) 0%	0 (0) 0%	1 (1) 6.67%
Moderate	0 (0) 0%	1 (1) 6.67%	1 (1) 6.67%
Hypokalemia			
Total	0 (0) 0%	2 (3) 13.33%	1 (1) 6.67%
Mild	0 (0) 0%	1 (1) 6.67%	0 (0) 0%
Moderate	0 (0) 0%	2 (2) 13.33%	1 (1) 6.67%
Nausea			
Total	1 (1) 6.67%	1 (1) 6.67%	1 (2) 6.67%
Mild	1 (1) 6.67%	1 (1) 6.67%	1 (2) 6.67%
Decreased appetite			
Total	0 (0) 0%	0 (0) 0%	2 (2) 13.33%
Mild	0 (0) 0%	0 (0) 0%	2 (2) 13.33%
Hemoglobin decreased			
Total	0 (0) 0%	1 (1) 6.67%	1 (1) 6.67%
Mild	0 (0) 0%	1 (1) 6.67%	1 (1) 6.67%
Alanine aminotransferase increased			
Total	0 (0) 0%	0 (0) 0%	1 (2) 6.67%
Mild	0 (0) 0%	0 (0) 0%	1 (2) 6.67%
Aspartate aminotransferase increased			
Total	0 (0) 0%	0 (0) 0%	1 (2) 6.67%
Mild	0 (0) 0%	0 (0) 0%	1 (2) 6.67%
Blood bilirubin increased			
Total	0 (0) 0%	0 (0) 0%	1 (1) 6.67%
Mild	0 (0) 0%	0 (0) 0%	1 (1) 6.67%
Diarrhea			
Total	0 (0) 0%	1 (1) 6.67%	0 (0) 0%
Mild	0 (0) 0%	1 (1) 6.67%	0 (0) 0%
Dizziness			
Total	0 (0) 0%	1 (1) 6.67%	0 (0) 0%
Mild	0 (0) 0%	1 (1) 6.67%	0 (0) 0%
Erythema			

Severity	IM (n = 15)	IN (n = 15)	Placebo (n = 15)
Total	0 (0) 0%	1 (1) 6.67%	0 (0) 0%
Mild	0 (0) 0%	1 (1) 6.67%	0 (0) 0%
Eye discomfort			
Total	0 (0) 0%	1 (1) 6.67%	0 (0) 0%
Mild	0 (0) 0%	1 (1) 6.67%	0 (0) 0%
Fever			
Total	0 (0) 0%	1 (1) 6.67%	0 (0) 0%
Severe	0 (0) 0%	1 (1) 6.67%	0 (0) 0%
Hyperglycemia			
Total	1 (1) 6.67%	0 (0) 0%	0 (0) 0%
Moderate	1 (1) 6.67%	0 (0) 0%	0 (0) 0%
Hypophosphatemia			
Total	1 (1) 6.67%	0 (0) 0%	0 (0) 0%
Mild	1 (1) 6.67%	0 (0) 0%	0 (0) 0%
Injection site ecchymosis			
Total	1 (1) 6.67%	0 (0) 0%	0 (0) 0%
Mild	1 (1) 6.67%	0 (0) 0%	0 (0) 0%
Migraine			
Total	1 (1) 6.67%	0 (0) 0%	0 (0) 0%
Moderate	1 (1) 6.67%	0 (0) 0%	0 (0) 0%
Muscle weakness lower limb			
Total	0 (0) 0%	1 (1) 6.67%	0 (0) 0%
Mild	0 (0) 0%	1 (1) 6.67%	0 (0) 0%
Numbness			
Total	1 (1) 6.67%	0 (0) 0%	0 (0) 0%
Mild	1 (1) 6.67%	0 (0) 0%	0 (0) 0%
Pain			
Total	0 (0) 0%	1 (1) 6.67%	0 (0) 0%
Mild	0 (0) 0%	1 (1) 6.67%	0 (0) 0%
Paresthesia			
Total	1 (1) 6.67%	0 (0) 0%	0 (0) 0%
Mild	1 (1) 6.67%	0 (0) 0%	0 (0) 0%
Pruritus			
Total	0 (0) 0%	1 (1) 6.67%	0 (0) 0%
Mild	0 (0) 0%	1 (1) 6.67%	0 (0) 0%
Vertigo			
Total	0 (0) 0%	1 (1) 6.67%	0 (0) 0%
Mild	0 (0) 0%	1 (1) 6.67%	0 (0) 0%
WBC decreased			
Total	1 (1) 6.67%	0 (0) 0%	0 (0) 0%

Severity	IM (n = 15)	IN (n = 15)	Placebo (n = 15)
Mild	1 (1) 6.67%	0 (0) 0%	0 (0) 0%
WBC increased			
Total	0 (0) 0%	0 (0) 0%	1 (1) 6.67%
Mild	0 (0) 0%	0 (0) 0%	1 (1) 6.67%

Table S6: Compare combined vaccine group (IM + IN) vs placebo for proportion of participants with all AEs (including unrelated) of combined and individual grades up to the final study visit

**Severity**	**Total # AEs**	**IM + IN (n = 30)**	**Placebo (n = 15)**	**p value**
Fatigue				
Total	31 (50) 68.89%	21 (32) 70%	10 (18) 66.67%	1.000
Mild	23 (31) 51.11%	15 (19) 50%	8 (12) 53.33%	NA
Moderate	13 (17) 28.89%	9 (12) 30%	4 (5) 26.67%	NA
Severe	2 (2) 4.44%	1 (1) 3.33%	1 (1) 6.67%	NA
Injection site pain				
Total	27 (41) 60%	21 (33) 70%	6 (8) 40%	0.107
Mild	27 (40) 60%	21 (32) 70%	6 (8) 40%	NA
Moderate	1 (1) 2.22%	1 (1) 3.33%	0 (0) 0%	NA
Headache				
Total	26 (44) 57.78%	17 (31) 56.67%	9 (13) 60%	1.000
Mild	22 (35) 48.89%	15 (24) 50%	7 (11) 46.67%	NA
Moderate	8 (9) 17.78%	6 (7) 20%	2 (2) 13.33%	NA
Sore throat				
Total	23 (30) 51.11%	14 (19) 46.67%	9 (11) 60%	0.598
Mild	19 (22) 42.22%	11 (14) 36.67%	8 (8) 53.33%	NA
Moderate	8 (8) 17.78%	5 (5) 16.67%	3 (3) 20%	NA
Nasal congestion				
Total	22 (27) 48.89%	12 (15) 40%	10 (12) 66.67%	0.170
Mild	20 (24) 44.44%	10 (12) 33.33%	10 (12) 66.67%	NA
Moderate	3 (3) 6.67%	3 (3) 10%	0 (0) 0%	NA
Respiratory rate				
Total	21 (30) 46.67%	15 (21) 50%	6 (9) 40%	0.751
Mild	21 (30) 46.67%	15 (21) 50%	6 (9) 40%	NA
Cough				
Total	19 (23) 42.22%	12 (16) 40%	7 (7) 46.67%	0.915
Mild	11 (14) 24.44%	7 (10) 23.33%	4 (4) 26.67%	NA
Moderate	8 (8) 17.78%	5 (5) 16.67%	3 (3) 20%	NA
Severe	1 (1) 2.22%	1 (1) 3.33%	0 (0) 0%	NA
Myalgia				
Total	19 (25) 42.22%	12 (16) 40%	7 (9) 46.67%	0.915
Mild	16 (20) 35.56%	10 (13) 33.33%	6 (7) 40%	NA
Moderate	5 (5) 11.11%	3 (3) 10%	2 (2) 13.33%	NA
Hyperglycemia				
Total	18 (28) 40%	13 (19) 43.33%	5 (9) 33.33%	0.747
Mild	14 (20) 31.11%	9 (12) 30%	5 (8) 33.33%	NA
Moderate	8 (8) 17.78%	7 (7) 23.33%	1 (1) 6.67%	NA
Bradycardia				

Severity	Total # AEs	IM + IN (n = 30)	Placebo (n = 15)	p value
Total	14 (24) 31.11%	9 (15) 30%	5 (9) 33.33%	1.000
Mild	10 (10) 22.22%	5 (5) 16.67%	5 (5) 33.33%	NA
Moderate	5 (7) 11.11%	3 (5) 10%	2 (2) 13.33%	NA
Severe	3 (7) 6.67%	1 (5) 3.33%	2 (2) 13.33%	NA
Hypokalemia				
Total	14 (19) 31.11%	8 (11) 26.67%	6 (8) 40%	0.569
Mild	11 (13) 24.44%	8 (8) 26.67%	3 (5) 20%	NA
Moderate	6 (6) 13.33%	3 (3) 10%	3 (3) 20%	NA
Hemoglobin decreased				
Total	13 (18) 28.89%	9 (12) 30%	4 (6) 26.67%	1.000
Mild	8 (11) 17.78%	4 (5) 13.33%	4 (6) 26.67%	NA
Moderate	5 (6) 11.11%	5 (6) 16.67%	0 (0) 0%	NA
Severe	1 (1) 2.22%	1 (1) 3.33%	0 (0) 0%	NA
CPK increased				
Total	12 (16) 26.67%	7 (8) 23.33%	5 (8) 33.33%	0.721
Mild	5 (6) 11.11%	2 (2) 6.67%	3 (4) 20%	NA
Moderate	4 (4) 8.89%	2 (2) 6.67%	2 (2) 13.33%	NA
Severe	3 (3) 6.67%	1 (1) 3.33%	2 (2) 13.33%	NA
Life-threatening*	3 (3) 6.67%	3 (3) 10%	0 (0) 0%	NA
Neutrophil count decreased				
Total	12 (21) 26.67%	6 (9) 20%	6 (12) 40%	0.283
Mild	8 (11) 17.78%	5 (6) 16.67%	3 (5) 20%	NA
Moderate	5 (8) 11.11%	2 (3) 6.67%	3 (5) 20%	NA
Severe	2 (2) 4.44%	0 (0) 0%	2 (2) 13.33%	NA
Rhinorrhea				
Total	12 (14) 26.67%	8 (10) 26.67%	4 (4) 26.67%	1.000
Mild	9 (11) 20%	5 (7) 16.67%	4 (4) 26.67%	NA
Moderate	3 (3) 6.67%	3 (3) 10%	0 (0) 0%	NA
Chills				
Total	11 (12) 24.44%	7 (7) 23.33%	4 (5) 26.67%	1.000
Mild	9 (10) 20%	5 (5) 16.67%	4 (5) 26.67%	NA
Moderate	1 (1) 2.22%	1 (1) 3.33%	0 (0) 0%	NA
Severe	1 (1) 2.22%	1 (1) 3.33%	0 (0) 0%	NA
Sneezing				
Total	10 (11) 22.22%	4 (5) 13.33%	6 (6) 40%	0.099
Mild	7 (8) 15.56%	2 (3) 6.67%	5 (5) 33.33%	NA
Moderate	3 (3) 6.67%	2 (2) 6.67%	1 (1) 6.67%	NA
COVID-19				
Total	9 (9) 20%	4 (4) 13.33%	5 (5) 33.33%	0.236
Mild	5 (5) 11.11%	1 (1) 3.33%	4 (4) 26.67%	NA
Moderate	4 (4) 8.89%	3 (3) 10%	1 (1) 6.67%	NA

Severity	Total # AEs	IM + IN (n = 30)	Placebo (n = 15)	p value
Hoarseness				
Total	9 (11) 20%	6 (8) 20%	3 (3) 20%	1.000
Mild	9 (11) 20%	6 (8) 20%	3 (3) 20%	NA
Arthralgia				
Total	7 (8) 15.56%	6 (7) 20%	1 (1) 6.67%	0.467
Mild	7 (8) 15.56%	6 (7) 20%	1 (1) 6.67%	NA
Aspartate aminotransferase increased				
Total	7 (8) 15.56%	5 (5) 16.67%	2 (3) 13.33%	1.000
Mild	5 (6) 11.11%	3 (3) 10%	2 (3) 13.33%	NA
Moderate	1 (1) 2.22%	1 (1) 3.33%	0 (0) 0%	NA
Severe	1 (1) 2.22%	1 (1) 3.33%	0 (0) 0%	NA
Decreased appetite				
Total	7 (9) 15.56%	3 (5) 10%	4 (4) 26.67%	0.309
Mild	6 (8) 13.33%	2 (4) 6.67%	4 (4) 26.67%	NA
Moderate	1 (1) 2.22%	1 (1) 3.33%	0 (0) 0%	NA
Upper respiratory infection				
Total	7 (8) 15.56%	6 (7) 20%	1 (1) 6.67%	0.467
Mild	3 (3) 6.67%	3 (3) 10%	0 (0) 0%	NA
Moderate	5 (5) 11.11%	4 (4) 13.33%	1 (1) 6.67%	NA
Fever				
Total	6 (6) 13.33%	4 (4) 13.33%	2 (2) 13.33%	1.000
Mild	3 (3) 6.67%	2 (2) 6.67%	1 (1) 6.67%	NA
Moderate	2 (2) 4.44%	1 (1) 3.33%	1 (1) 6.67%	NA
Severe	1 (1) 2.22%	1 (1) 3.33%	0 (0) 0%	NA
Hyponatremia				
Total	6 (7) 13.33%	5 (6) 16.67%	1 (1) 6.67%	0.642
Mild	6 (7) 13.33%	5 (6) 16.67%	1 (1) 6.67%	NA
Hypophosphatemia				
Total	6 (6) 13.33%	2 (2) 6.67%	4 (4) 26.67%	0.163
Mild	3 (3) 6.67%	2 (2) 6.67%	1 (1) 6.67%	NA
Moderate	3 (3) 6.67%	0 (0) 0%	3 (3) 20%	NA
Lymphocyte count decreased				
Total	6 (8) 13.33%	2 (3) 6.67%	4 (5) 26.67%	0.163
Mild	5 (7) 11.11%	2 (3) 6.67%	3 (4) 20%	NA
Moderate	1 (1) 2.22%	0 (0) 0%	1 (1) 6.67%	NA
Nausea				
Total	5 (6) 11.11%	3 (3) 10%	2 (3) 13.33%	1.000
Mild	5 (6) 11.11%	3 (3) 10%	2 (3) 13.33%	NA
Abdominal cramps				
Total	4 (5) 8.89%	3 (3) 10%	1 (2) 6.67%	1.000
Mild	3 (4) 6.67%	2 (2) 6.67%	1 (2) 6.67%	NA

Severity	Total # AEs	IM + IN (n = 30)	Placebo (n = 15)	p value
Moderate	1 (1) 2.22%	1 (1) 3.33%	0 (0) 0%	NA
WBC decreased				
Total	4 (4) 8.89%	1 (1) 3.33%	3 (3) 20%	0.195
Mild	2 (2) 4.44%	1 (1) 3.33%	1 (1) 6.67%	NA
Moderate	2 (2) 4.44%	0 (0) 0%	2 (2) 13.33%	NA
Alanine aminotransferase increased				
Total	3 (4) 6.67%	2 (2) 6.67%	1 (2) 6.67%	1.000
Mild	2 (3) 4.44%	1 (1) 3.33%	1 (2) 6.67%	NA
Moderate	1 (1) 2.22%	1 (1) 3.33%	0 (0) 0%	NA
Diarrhea				
Total	3 (3) 6.67%	1 (1) 3.33%	2 (2) 13.33%	0.526
Mild	3 (3) 6.67%	1 (1) 3.33%	2 (2) 13.33%	NA
Eye discomfort				
Total	3 (3) 6.67%	2 (2) 6.67%	1 (1) 6.67%	1.000
Mild	3 (3) 6.67%	2 (2) 6.67%	1 (1) 6.67%	NA
Feverish				
Total	3 (3) 6.67%	3 (3) 10%	0 (0) 0%	0.526
Mild	2 (2) 4.44%	2 (2) 6.67%	0 (0) 0%	NA
Moderate	1 (1) 2.22%	1 (1) 3.33%	0 (0) 0%	NA
Hypoglycemia				
Total	3 (3) 6.67%	2 (2) 6.67%	1 (1) 6.67%	1.000
Mild	2 (2) 4.44%	1 (1) 3.33%	1 (1) 6.67%	NA
Moderate	1 (1) 2.22%	1 (1) 3.33%	0 (0) 0%	NA
Vomiting				
Total	3 (3) 6.67%	2 (2) 6.67%	1 (1) 6.67%	1.000
Mild	3 (3) 6.67%	2 (2) 6.67%	1 (1) 6.67%	NA
WBC increased				
Total	3 (3) 6.67%	2 (2) 6.67%	1 (1) 6.67%	1.000
Mild	3 (3) 6.67%	2 (2) 6.67%	1 (1) 6.67%	NA
Abdominal pain				
Total	2 (2) 4.44%	2 (2) 6.67%	0 (0) 0%	0.798
Mild	1 (1) 2.22%	1 (1) 3.33%	0 (0) 0%	NA
Severe	1 (1) 2.22%	1 (1) 3.33%	0 (0) 0%	NA
Back pain				
Total	2 (2) 4.44%	1 (1) 3.33%	1 (1) 6.67%	1.000
Mild	1 (1) 2.22%	1 (1) 3.33%	0 (0) 0%	NA
Moderate	1 (1) 2.22%	0 (0) 0%	1 (1) 6.67%	NA
Blood bilirubin increased				
Total	2 (2) 4.44%	1 (1) 3.33%	1 (1) 6.67%	1.000
Mild	2 (2) 4.44%	1 (1) 3.33%	1 (1) 6.67%	NA

Severity	Total # AEs	IM + IN (n = 30)	Placebo (n = 15)	p value
Chest pain				
Total	2 (2) 4.44%	1 (1) 3.33%	1 (1) 6.67%	1.000
Mild	1 (1) 2.22%	1 (1) 3.33%	0 (0) 0%	NA
Moderate	1 (1) 2.22%	0 (0) 0%	1 (1) 6.67%	NA
Ecchymosis				
Total	2 (2) 4.44%	1 (1) 3.33%	1 (1) 6.67%	1.000
Mild	2 (2) 4.44%	1 (1) 3.33%	1 (1) 6.67%	NA
Hyperkalemia				
Total	2 (2) 4.44%	2 (2) 6.67%	0 (0) 0%	0.798
Mild	2 (2) 4.44%	2 (2) 6.67%	0 (0) 0%	NA
Platelet count decreased				
Total	2 (2) 4.44%	0 (0) 0%	2 (2) 13.33%	0.201
Moderate	1 (1) 2.22%	0 (0) 0%	1 (1) 6.67%	NA
Severe	1 (1) 2.22%	0 (0) 0%	1 (1) 6.67%	NA
Pruritus				
Total	2 (2) 4.44%	2 (2) 6.67%	0 (0) 0%	0.798
Mild	2 (2) 4.44%	2 (2) 6.67%	0 (0) 0%	NA
Rhinovirus infection				
Total	2 (2) 4.44%	1 (1) 3.33%	1 (1) 6.67%	1.000
Mild	2 (2) 4.44%	1 (1) 3.33%	1 (1) 6.67%	NA
Shortness of breath				
Total	2 (2) 4.44%	0 (0) 0%	2 (2) 13.33%	0.201
Moderate	2 (2) 4.44%	0 (0) 0%	2 (2) 13.33%	NA
Shoulder pain				
Total	2 (2) 4.44%	1 (1) 3.33%	1 (1) 6.67%	1.000
Mild	2 (2) 4.44%	1 (1) 3.33%	1 (1) 6.67%	NA
Anterior cruciate ligament tear				
Total	1 (1) 2.22%	0 (0) 0%	1 (1) 6.67%	0.721
Severe	1 (1) 2.22%	0 (0) 0%	1 (1) 6.67%	NA
BUN increased				
Total	1 (1) 2.22%	1 (1) 3.33%	0 (0) 0%	1.000
Mild	1 (1) 2.22%	1 (1) 3.33%	0 (0) 0%	NA
Cotton wool spots				
Total	1 (1) 2.22%	1 (1) 3.33%	0 (0) 0%	1.000
Mild	1 (1) 2.22%	1 (1) 3.33%	0 (0) 0%	NA
Dental implant user				
Total	1 (1) 2.22%	0 (0) 0%	1 (1) 6.67%	0.721
Mild	1 (1) 2.22%	0 (0) 0%	1 (1) 6.67%	NA
Depression				
Total	1 (1) 2.22%	0 (0) 0%	1 (1) 6.67%	0.721

Severity	Total # AEs	IM + IN (n = 30)	Placebo (n = 15)	p value
Mild	1 (1) 2.22%	0 (0) 0%	1 (1) 6.67%	NA
Diaphoresis				
Total	1 (1) 2.22%	1 (1) 3.33%	0 (0) 0%	1.000
Mild	1 (1) 2.22%	1 (1) 3.33%	0 (0) 0%	NA
Difficulty thinking				
Total	1 (1) 2.22%	0 (0) 0%	1 (1) 6.67%	0.721
Mild	1 (1) 2.22%	0 (0) 0%	1 (1) 6.67%	NA
Dizziness				
Total	1 (1) 2.22%	1 (1) 3.33%	0 (0) 0%	1.000
Mild	1 (1) 2.22%	1 (1) 3.33%	0 (0) 0%	NA
Dyspepsia				
Total	1 (1) 2.22%	1 (1) 3.33%	0 (0) 0%	1.000
Mild	1 (1) 2.22%	1 (1) 3.33%	0 (0) 0%	NA
Erythema				
Total	1 (1) 2.22%	1 (1) 3.33%	0 (0) 0%	1.000
Mild	1 (1) 2.22%	1 (1) 3.33%	0 (0) 0%	NA
Fracture				
Total	1 (1) 2.22%	0 (0) 0%	1 (1) 6.67%	0.721
Severe	1 (1) 2.22%	0 (0) 0%	1 (1) 6.67%	NA
Hypotension				
Total	1 (1) 2.22%	1 (1) 3.33%	0 (0) 0%	1.000
Mild	1 (1) 2.22%	1 (1) 3.33%	0 (0) 0%	NA
Injection site ecchymosis				
Total	1 (1) 2.22%	1 (1) 3.33%	0 (0) 0%	1.000
Mild	1 (1) 2.22%	1 (1) 3.33%	0 (0) 0%	NA
Injection site itching				
Total	1 (1) 2.22%	0 (0) 0%	1 (1) 6.67%	0.721
Mild	1 (1) 2.22%	0 (0) 0%	1 (1) 6.67%	NA
Itchy throat				
Total	1 (1) 2.22%	0 (0) 0%	1 (1) 6.67%	0.721
Mild	1 (1) 2.22%	0 (0) 0%	1 (1) 6.67%	NA
Loss of smell				
Total	1 (1) 2.22%	0 (0) 0%	1 (1) 6.67%	0.721
Mild	1 (1) 2.22%	0 (0) 0%	1 (1) 6.67%	NA
Low back pain				
Total	1 (1) 2.22%	0 (0) 0%	1 (1) 6.67%	0.721
Mild	1 (1) 2.22%	0 (0) 0%	1 (1) 6.67%	NA
Meniscus tear of knee				
Total	1 (1) 2.22%	0 (0) 0%	1 (1) 6.67%	0.721
Severe	1 (1) 2.22%	0 (0) 0%	1 (1) 6.67%	NA

Severity	Total # AEs	IM + IN (n = 30)	Placebo (n = 15)	p value
Menstrual irregularity				
Total	1 (1) 2.22%	1 (1) 3.33%	0 (0) 0%	1.000
Mild	1 (1) 2.22%	1 (1) 3.33%	0 (0) 0%	NA
Migraine				
Total	1 (1) 2.22%	1 (1) 3.33%	0 (0) 0%	1.000
Moderate	1 (1) 2.22%	1 (1) 3.33%	0 (0) 0%	NA
Muscle soreness				
Total	1 (1) 2.22%	1 (1) 3.33%	0 (0) 0%	1.000
Mild	1 (1) 2.22%	1 (1) 3.33%	0 (0) 0%	NA
Muscle strain				
Total	1 (1) 2.22%	1 (1) 3.33%	0 (0) 0%	1.000
Mild	1 (1) 2.22%	1 (1) 3.33%	0 (0) 0%	NA
Muscle weakness lower limb				
Total	1 (1) 2.22%	1 (1) 3.33%	0 (0) 0%	1.000
Mild	1 (1) 2.22%	1 (1) 3.33%	0 (0) 0%	NA
Neck pain				
Total	1 (1) 2.22%	0 (0) 0%	1 (1) 6.67%	0.721
Mild	1 (1) 2.22%	0 (0) 0%	1 (1) 6.67%	NA
Numbness				
Total	1 (1) 2.22%	1 (1) 3.33%	0 (0) 0%	1.000
Mild	1 (1) 2.22%	1 (1) 3.33%	0 (0) 0%	NA
Pain				
Total	1 (1) 2.22%	1 (1) 3.33%	0 (0) 0%	1.000
Mild	1 (1) 2.22%	1 (1) 3.33%	0 (0) 0%	NA
Paresthesia				
Total	1 (1) 2.22%	1 (1) 3.33%	0 (0) 0%	1.000
Mild	1 (1) 2.22%	1 (1) 3.33%	0 (0) 0%	NA
Puncture wound				
Total	1 (1) 2.22%	1 (1) 3.33%	0 (0) 0%	1.000
Mild	1 (1) 2.22%	1 (1) 3.33%	0 (0) 0%	NA
Rash				
Total	1 (1) 2.22%	0 (0) 0%	1 (1) 6.67%	0.721
Mild	1 (1) 2.22%	0 (0) 0%	1 (1) 6.67%	NA
Respiratory syncytial virus infection				
Total	1 (1) 2.22%	1 (1) 3.33%	0 (0) 0%	1.000
Moderate	1 (1) 2.22%	1 (1) 3.33%	0 (0) 0%	NA
Respiratory tract congestion				
Total	1 (2) 2.22%	1 (2) 3.33%	0 (0) 0%	1.000
Mild	1 (2) 2.22%	1 (2) 3.33%	0 (0) 0%	NA

Severity	Total # AEs	IM + IN (n = 30)	Placebo (n = 15)	p value
Scar				
Total	1 (1) 2.22%	0 (0) 0%	1 (1) 6.67%	0.721
Mild	1 (1) 2.22%	0 (0) 0%	1 (1) 6.67%	NA
Skin rash				
Total	1 (1) 2.22%	1 (1) 3.33%	0 (0) 0%	1.000
Mild	1 (1) 2.22%	1 (1) 3.33%	0 (0) 0%	NA
Smell alteration				
Total	1 (1) 2.22%	1 (1) 3.33%	0 (0) 0%	1.000
Mild	1 (1) 2.22%	1 (1) 3.33%	0 (0) 0%	NA
Syncope				
Total	1 (1) 2.22%	1 (1) 3.33%	0 (0) 0%	1.000
Moderate	1 (1) 2.22%	1 (1) 3.33%	0 (0) 0%	NA
Systolic hypertension				
Total	1 (1) 2.22%	1 (1) 3.33%	0 (0) 0%	1.000
Mild	1 (1) 2.22%	1 (1) 3.33%	0 (0) 0%	NA
Tachycardia				
Total	1 (1) 2.22%	1 (1) 3.33%	0 (0) 0%	1.000
Mild	1 (1) 2.22%	1 (1) 3.33%	0 (0) 0%	NA
Taste alteration				
Total	1 (1) 2.22%	1 (1) 3.33%	0 (0) 0%	1.000
Mild	1 (1) 2.22%	1 (1) 3.33%	0 (0) 0%	NA
Tendinitis				
Total	1 (1) 2.22%	1 (1) 3.33%	0 (0) 0%	1.000
Mild	1 (1) 2.22%	1 (1) 3.33%	0 (0) 0%	NA
Uric acid high				
Total	1 (1) 2.22%	0 (0) 0%	1 (1) 6.67%	0.721
Mild	1 (1) 2.22%	0 (0) 0%	1 (1) 6.67%	NA
Urinary tract infection				
Total	1 (1) 2.22%	1 (1) 3.33%	0 (0) 0%	1.000
Severe	1 (1) 2.22%	1 (1) 3.33%	0 (0) 0%	NA
Venipuncture site bruise				
Total	1 (1) 2.22%	1 (1) 3.33%	0 (0) 0%	1.000
Mild	1 (1) 2.22%	1 (1) 3.33%	0 (0) 0%	NA
Venipuncture site pain				
Total	1 (1) 2.22%	1 (1) 3.33%	0 (0) 0%	1.000
Mild	1 (1) 2.22%	1 (1) 3.33%	0 (0) 0%	NA
Vertigo				
Total	1 (1) 2.22%	1 (1) 3.33%	0 (0) 0%	1.000
Mild	1 (1) 2.22%	1 (1) 3.33%	0 (0) 0%	NA

Severity	Total # AEs	IM + IN (n = 30)	Placebo (n = 15)	p value
Weight loss				
Total	1 (1) 2.22%	1 (1) 3.33%	0 (0) 0%	1.000
Mild	1 (1) 2.22%	1 (1) 3.33%	0 (0) 0%	NA

A two-sided test of proportions was performed on AE proportions (significance threshold: $p < 0.05$).

* While adverse events that are grade level 4 per the FDA guidance document (Toxicity Grading Scale for Healthy Adult and Adolescent Volunteers Enrolled in Preventive Vaccine Clinical Trials) are defined as "life-threatening", the elevated creatine kinase (CK) levels noted in 3 participants during the study were not considered dangerous or clinically significant by blinded study staff. They were asymptomatic, not associated with any other clinical or laboratory abnormalities, attributed to recent intense exercise, and resolved without any medical intervention. One was detected in a participant at 7 days after the booster dose with IM BPL-1357. The second grade 4 CK elevation was detected in an IN BPL-1357 participant 7 days after the prime dose. This participant had mildly elevated CK at baseline which persisted throughout the study and did not experience an increase in CK after the booster dose of IN BPL-1357. The third participant with grade 4 CK elevation had received the prime dose of IN BPL-1357 twenty-four days earlier and had mildly elevated CK at baseline. After receiving the booster dose of IN BPL-1357, his CK continued to improve back to baseline levels.

Table S7: Number of mucosal and serum samples for each timepoint

Sample Type	Group	Visit	Total.N
mucosal	IM	V1D0	15
mucosal	IM	V1D7	14
mucosal	IM	V1D14	14
mucosal	IM	V2D0	15
mucosal	IM	V2D7	15
mucosal	IM	V2D14	15
mucosal	IM	V2D28	14
mucosal	IM	V2D56	13
mucosal	IM	V2D182	14
mucosal	IN	V1D0	14
mucosal	IN	V1D7	14
mucosal	IN	V1D14	13
mucosal	IN	V2D0	14
mucosal	IN	V2D7	14
mucosal	IN	V2D14	14
mucosal	IN	V2D28	14
mucosal	IN	V2D56	14
mucosal	IN	V2D182	14
mucosal	placebo	V1D0	15
mucosal	placebo	V1D7	14
mucosal	placebo	V1D14	13
mucosal	placebo	V2D0	14
mucosal	placebo	V2D7	14
mucosal	placebo	V2D14	14
mucosal	placebo	V2D28	15
mucosal	placebo	V2D56	14
mucosal	placebo	V2D182	14
serum	IM	V1D0	15
serum	IM	V1D7	15
serum	IM	V1D14	15
serum	IM	V2D0	15
serum	IM	V2D7	15
serum	IM	V2D14	15
serum	IM	V2D28	14
serum	IM	V2D56	13
serum	IM	V2D182	14
serum	IN	V1D0	14
serum	IN	V1D7	14
serum	IN	V1D14	13
serum	IN	V2D0	14
serum	IN	V2D7	14
serum	IN	V2D14	14
serum	IN	V2D28	14

Sample Type	Group	Visit	Total.N
serum	IN	V2D56	14
serum	IN	V2D182	14
serum	placebo	V1D0	15
serum	placebo	V1D7	14
serum	placebo	V1D14	14
serum	placebo	V2D0	14
serum	placebo	V2D7	14
serum	placebo	V2D14	14
serum	placebo	V2D28	15
serum	placebo	V2D56	15
serum	placebo	V2D182	14

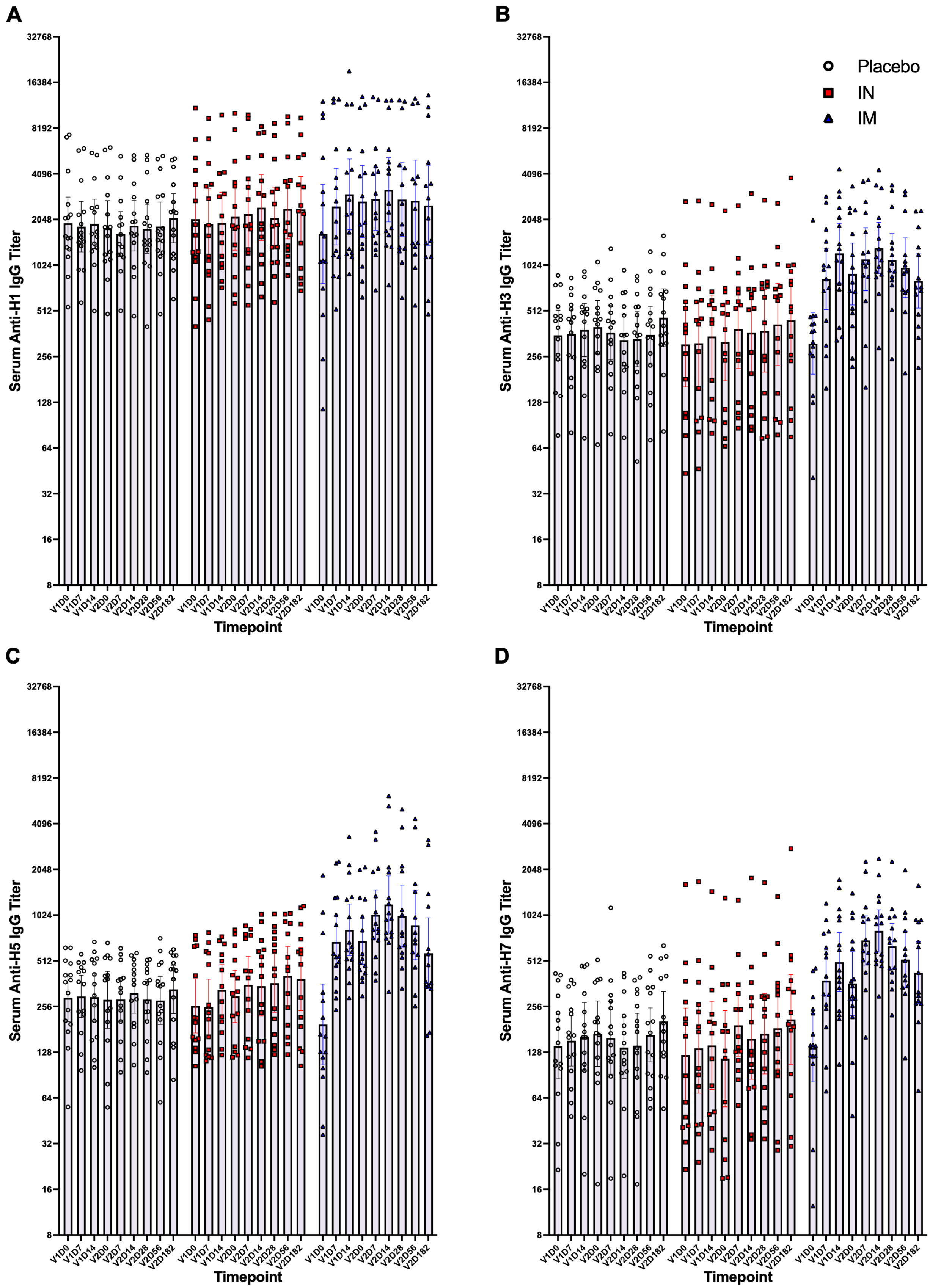
A
B
C
D
Serum Anti-H1 IgG Titer
Serum Anti-H3 IgG Titer
Serum Anti-H5 IgG Titer
Serum Anti-H7 IgG Titer
32768
16384
8192
4096
2048
1024
512
256
128
64
32
16
8
V1D0
V1D7
V1D14
V2D0
V2D7
V2D14
V2D28
V2D56
V2D182
Timepoint
Placebo
IN
IM

Figure S1: Serum IgG titers against H1, H3, H5, and H7 after IM and IN BPL-1357

Serum IgG geometric mean titers measured by enzyme-linked immunosorbent assay to vaccine HA (H1, H3, H5, H7) antigens are shown as absolute half-maximal effective concentration EC_{50} values for participants who received intramuscular (IM) BPL-1357 (n=15), intranasal (IN) BPL-1357 (n=14), and placebo (n=15). Titers are shown across all timepoints, including at baseline (vaccine 1 day 0 [V1D0]), 7 and 14 days after the prime vaccination (vaccine 1 day 7 [V1D7] and V1D14), prior to the booster vaccination (boost study day 0 which occurs 28 days after the prime dose [V2D0]), and after the booster vaccination (V2D7, V2D14, V2D28, V2D56. V2D182). Vertical lines denote 95% confidence intervals.

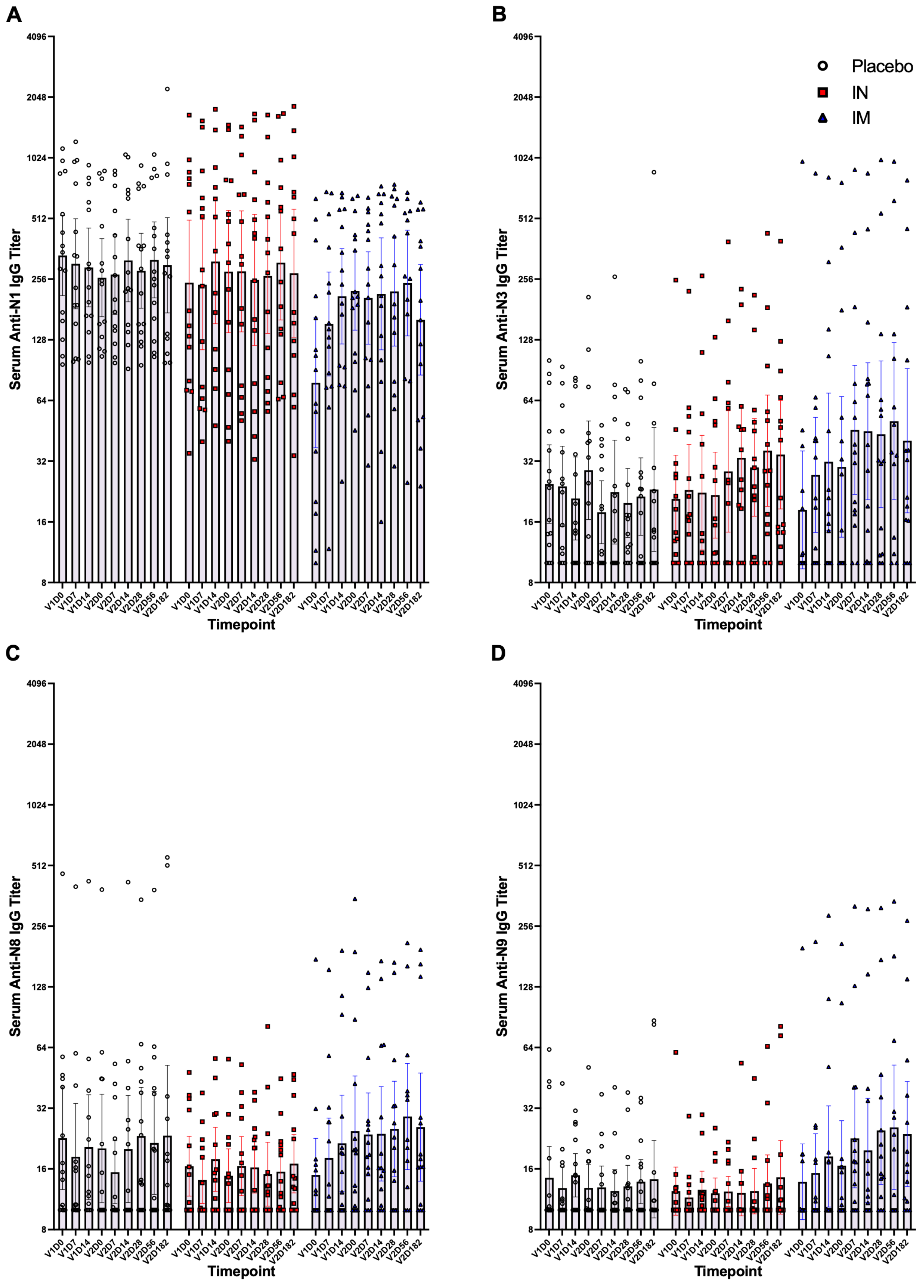

A
Serum Anti-N1 IgG Titer
B
Serum Anti-N3 IgG Titer
Placebo
IN
IM
C
Serum Anti-N8 IgG Titer
D
Serum Anti-N9 IgG Titer
4096
2048
1024
512
256
128
64
32
16
8
V1D0
V1D7
V1D14
V2D0
V2D7
V2D14
V2D28
V2D56
V2D182
Timepoint

Figure S2: Serum IgG titers against N1, N3, N8, and N9 after IM and IN BPL-1357

Serum IgG geometric mean titers measured by enzyme-linked immunosorbent assay to vaccine NA (N1, N3, N8, N9) antigens are shown as absolute half-maximal effective concentration EC_{50} values for participants who received intramuscular (IM) BPL-1357 (n=15), intranasal (IN) BPL-1357 (n=14), and placebo (n=15). Titers are shown across all timepoints, including at baseline (vaccine 1 day 0 [V1D0]), 7 and 14 days after the prime vaccination (vaccine 1 day 7 [V1D7] and V1D14), prior to the booster vaccination (boost study day 0 which occurs 28 days after the prime dose [V2D0]), and after the booster vaccination (V2D7, V2D14, V2D28, V2D56. V2D182). Vertical lines denote 95% confidence intervals.

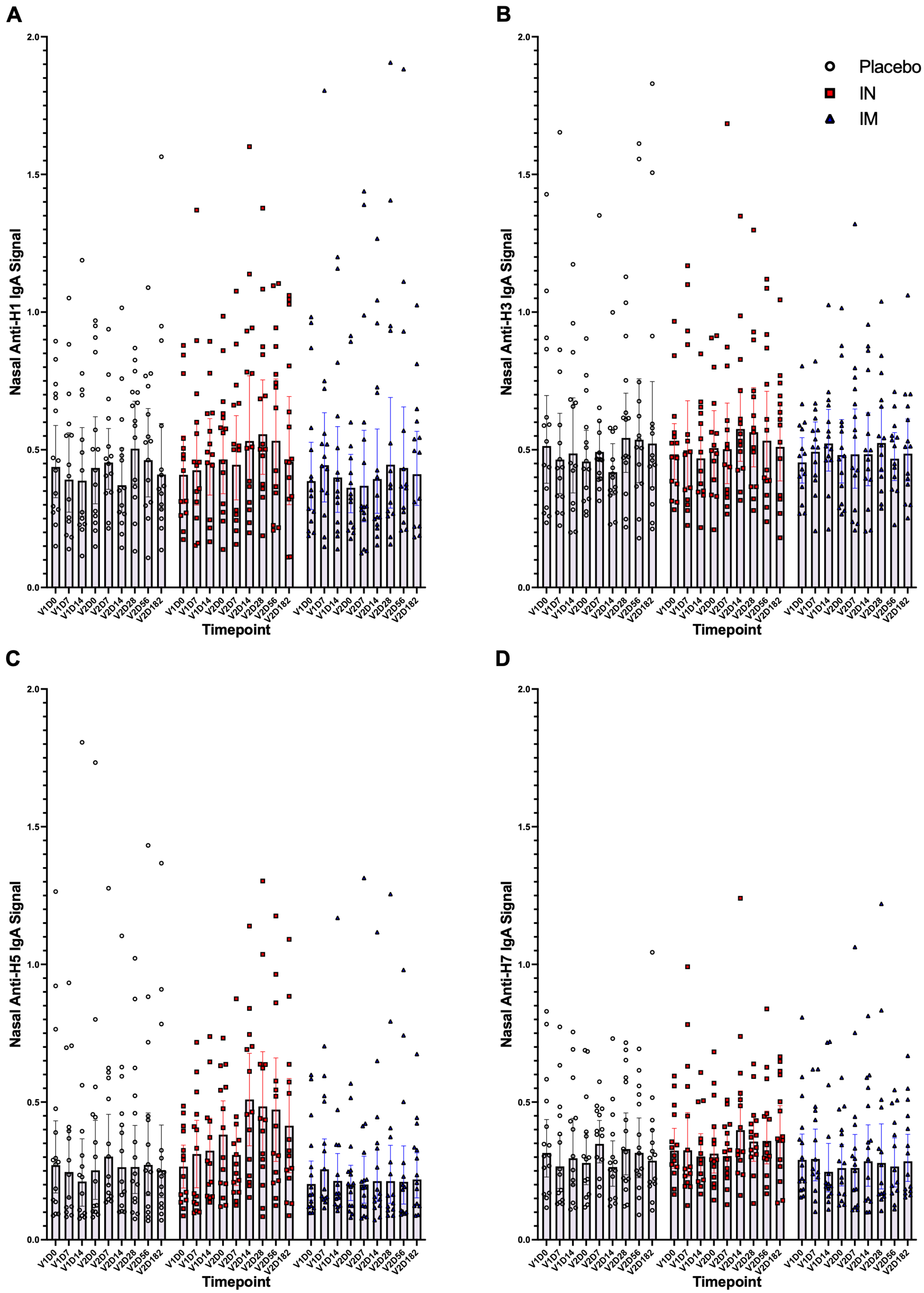
A
B
C
D
Placebo
IN
IM
Nasal Anti-H1 IgA Signal
Nasal Anti-H3 IgA Signal
Nasal Anti-H5 IgA Signal
Nasal Anti-H7 IgA Signal
0.0
0.5
1.0
1.5
2.0
V1D0
V1D7
V1D14
V2D0
V2D7
V2D14
V2D28
V2D56
V2D182
Timepoint

Figure S3: Nasal IgA signal against H1, H3, H5, and H7 after IM and IN BPL-1357

Nasal IgA geometric mean signal measured by enzyme-linked immunosorbent assay to vaccine HA (H1, H3, H5, H7) antigens are shown as fold change in optical density values from baseline for participants who received intramuscular (IM) BPL-1357 (n=15), intranasal (IN) BPL-1357 (n=14), and placebo (n=15). Data are shown across all timepoints, including at baseline (vaccine 1 day 0 [V1D0]), 7 and 14 days after the prime vaccination (vaccine 1 day 7 [V1D7] and V1D14), prior to the booster vaccination (boost study day 0 which occurs 28 days after the prime dose [V2D0]), and after the booster vaccination (V2D7, V2D14, V2D28, V2D56. V2D182). Vertical lines denote 95% confidence intervals.

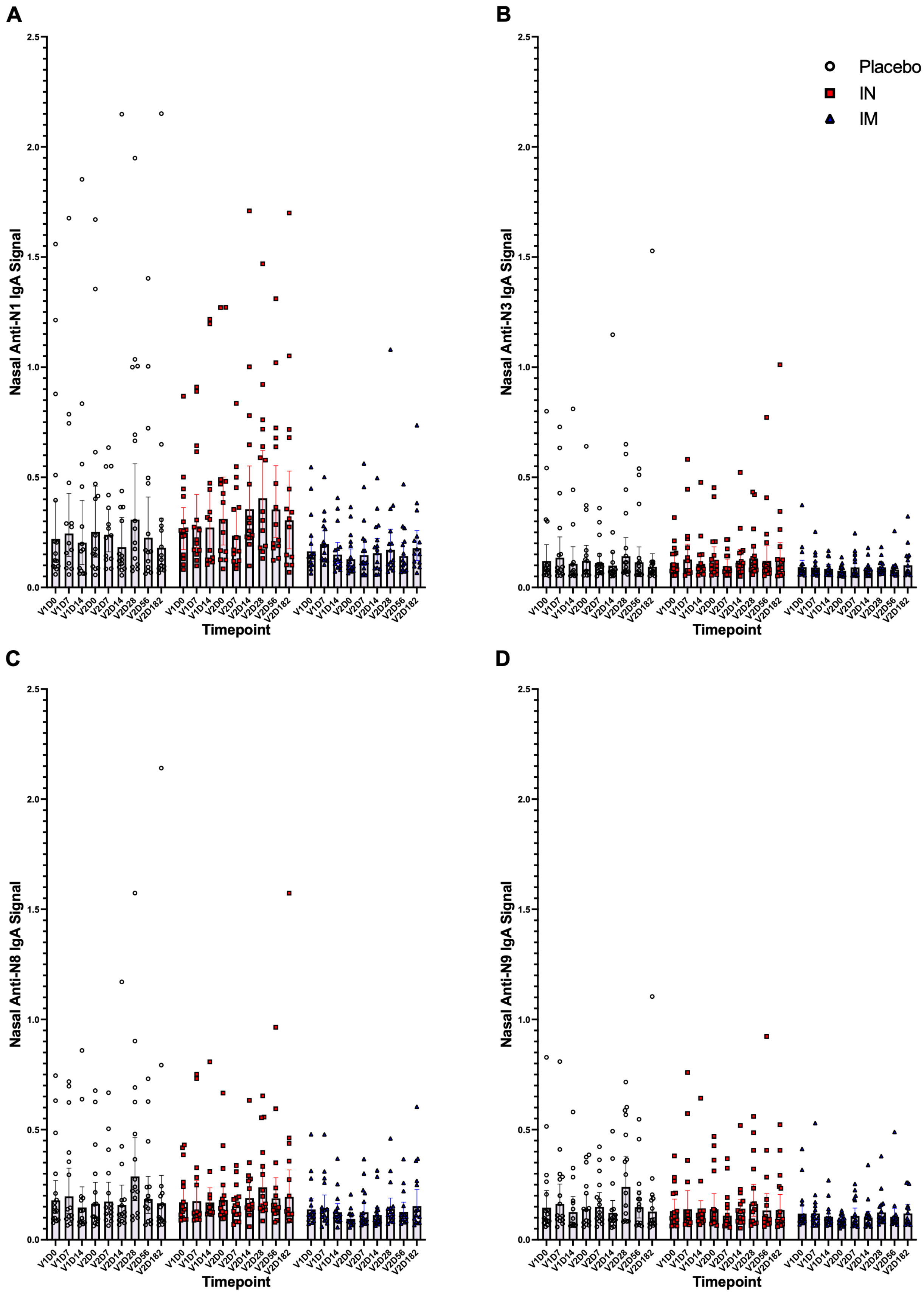
A
B
C
D
Nasal Anti-N1 IgA Signal
Nasal Anti-N3 IgA Signal
Nasal Anti-N8 IgA Signal
Nasal Anti-N9 IgA Signal
Timepoint
Placebo
IN
IM
V1D0
V1D7
V1D14
V2D0
V2D7
V2D14
V2D28
V2D56
V2D182

Figure S4: Nasal IgA signal against N1, N3, N8, and N9 after IM and IN BPL-1357

Nasal IgA geometric mean signal measured by enzyme-linked immunosorbent assay to vaccine NA (N1, N3, N8, N9) antigens are shown as fold change in optical density values from baseline for participants who received intramuscular (IM) BPL-1357 (n=15), intranasal (IN) BPL-1357 (n=14), and placebo (n=15). Data are shown across all timepoints, including at baseline (vaccine 1 day 0 [V1D0]), 7 and 14 days after the prime vaccination (vaccine 1 day 7 [V1D7] and V1D14), prior to the booster vaccination (boost study day 0 which occurs 28 days after the prime dose [V2D0]), and after the booster vaccination (V2D7, V2D14, V2D28, V2D56. V2D182). Vertical lines denote 95% confidence intervals.

CONSORT 2025 checklist of information to include when reporting a randomised trial

Section / Topic	No	CONSORT 2025 checklist item description	Reported on page no.
**Title and abstract**			
Title and structured abstract	1a	Identification as a randomised trial	1,2
	1b	Structured summary of the trial design, methods, results, and conclusions	2
**Open science**			
Trial registration	2	Name of trial registry, identifying number (with URL) and date of registration	9
Protocol and statistical analysis plan	3	Where the trial protocol and statistical analysis plan can be accessed	9
Data sharing	4	Where and how the individual de-identified participant data (including data dictionary), statistical code and any other materials can be accessed	12
Funding and conflicts of interest	5a	Sources of funding and other support (e.g., supply of drugs), and role of funders in the design, conduct, analysis and reporting of the trial	16
	5b	Financial and other conflicts of interest of the manuscript authors	16
**Introduction**			
Background and rationale	6	Scientific background and rationale	3
Objectives	7	Specific objectives related to benefits and harms	4
**Methods**			
Patient and public involvement	8	Details of patient or public involvement in the design, conduct and reporting of the trial	N/A
Trial design	9	Description of trial design including type of trial (e.g., parallel group, crossover), allocation ratio, and framework (e.g., superiority, equivalence, non-inferiority, exploratory)	8, 9
Changes to trial protocol	10	Important changes to the trial after it commenced including any outcomes or analyses that were not prespecified, with reason	11
Trial setting	11	Settings (e.g., community, hospital) and locations (e.g., countries, sites) where the trial was conducted	8
Eligibility criteria	12a	Eligibility criteria for participants	8, 9 supplemental
	12b	If applicable, eligibility criteria for sites and for individuals delivering the interventions (e.g., surgeons, physiotherapists)	N/A
Intervention and comparator	13	Intervention and comparator with sufficient details to allow replication. If relevant, where additional materials describing the intervention and comparator (e.g., intervention manual) can be accessed	8, 9, 10
Outcomes	14	Pre-specified primary and secondary outcomes, including the specific measurement variable (e.g., systolic blood pressure), analysis metric (e.g., change from baseline, final value, time to event), method of aggregation (e.g., median, proportion), and time point for each outcome	11, supplemental
Harms	15	How harms were defined and assessed (e.g., systematically, non-systematically)	9, 10, 12, supplemental
Sample size	16a	How sample size was determined, including all assumptions supporting the sample size calculation	11, supplemental
	16b	Explanation of any interim analyses and stopping guidelines	N/A
Randomisation:			

Sequence generation	17a	Who generated the random allocation sequence and the method used	9
	17b	Type of randomisation and details of any restriction (e.g., stratification, blocking and block size)	9
Allocation concealment mechanism	18	Mechanism used to implement the random allocation sequence (e.g., central computer/telephone; sequentially numbered, opaque, sealed containers), describing any steps to conceal the sequence until interventions were assigned	9
Implementation	19	Whether the personnel who enrolled and those who assigned participants to the interventions had access to the random allocation sequence	9
Blinding	20a	Who was blinded after assignment to interventions (e.g., participants, care providers, outcome assessors, data analysts)	9
	20b	If blinded, how blinding was achieved and description of the similarity of interventions	9
Statistical methods	21a	Statistical methods used to compare groups for primary and secondary outcomes, including harms	11, 12
	21b	Definition of who is included in each analysis (e.g., all randomised participants), and in which group	11, 12
	21c	How missing data were handled in the analysis	12
	21d	Methods for any additional analyses (e.g., subgroup and sensitivity analyses), distinguishing prespecified from post-hoc	11
**Results**			
Participant flow, including flow diagram	22a	For each group, the numbers of participants who were randomly assigned, received intended intervention, and were analysed for the primary outcome	4
	22b	For each group, losses and exclusions after randomisation, together with reasons	4
Recruitment	23a	Dates defining the periods of recruitment and follow-up for outcomes of benefits and harms	4
	23b	If relevant, why the trial ended or was stopped	N/A
Intervention and comparator delivery	24a	Intervention and comparator as they were actually administered (e.g., where appropriate, who delivered the intervention/comparator, how participants adhered, whether they were delivered as intended [fidelity])	4
	24b	Concomitant care received during the trial for each group	N/A
Baseline data	25	A table showing baseline demographic and clinical characteristics for each group	17
Numbers analysed, outcomes and estimation	26	For each primary and secondary outcome, by group:<br>● the number of participants included in the analysis<br>● the number of participants with available data at the outcome time point<br>● result for each group, and the estimated effect size and its precision (such as 95% confidence interval)<br>● for binary outcomes, presentation of both absolute and relative effect size	4- 6, 12, 17-22, supplemental
Harms	27	All harms or unintended events in each group	4, supplemental
Ancillary analyses	28	Any other analyses performed, including subgroup and sensitivity analyses, distinguishing pre-specified from post-hoc	5, 6, 12, supplemental
**Discussion**			
Interpretation	29	Interpretation consistent with results, balancing benefits and harms, and considering other relevant evidence	6, 7, 8
Limitations	30	Trial limitations, addressing sources of potential bias, imprecision, generalisability, and, if relevant, multiplicity of analyses	8

Abbreviated Title: BPL-1357 Phase 1
Protocol #: *000320*
Version Date: June 30, 2022

Title: Randomized, Double-Blinded, Placebo-Controlled, Phase 1 Study of the Safety and Immunogenicity of BPL-1357, A BPL-Inactivated, Whole-Virus, Universal Influenza Vaccine

Investigators:

Principal Investigator (PI): Matthew J. Memoli, MD, MS
Director, Laboratory of Infectious Diseases (LID) Clinical Studies Unit (CSU)
Laboratory of Infectious Diseases (LID)
National Institute of Allergy and Infectious Diseases (NIAID)
National Institutes of Health (NIH)
MSC 3203 33 North Dr Room 3E13C4
Bethesda, MD 20892-3203
Phone: 301-443-5971
Email: memolim@niaid.nih.gov

Investigational Agent:

Drug Name:	BPL-1357
Investigational New Drug (IND) Number:	027691
Sponsor:	Office of Clinical Research Policy and Regulatory Operations (OCRPRO), NIAID
Manufacturer:	Charles River Laboratories, Malvern, PA

Data and Safety Monitoring Board (DSMB): NIAID Intramural DSMB

TABLE OF CONTENTS

STATEMENT OF COMPLIANCE 8
1 PROTOCOL SUMMARY 9
1.1 Synopsis 9
1.2 Schema 10
1.3 Schedule of Activities 11
2 INTRODUCTION 13
2.1 Study Rationale 13
2.2 Background 13
2.3 Risk/Benefit Assessment 19
2.3.1 Known Potential Risks 19
2.3.1.1 Risks of BPL-1357 Influenza Vaccination 19
2.3.1.2 Risks of Placebo Administration 21
2.3.1.3 Risks of Blood Draw 21
2.3.1.4 Risks of Nasal Sampling 22
2.3.1.5 Risks of Urine Drug Testing 22
2.3.2 Known Potential Benefits 22
2.3.3 Assessment of Potential Risks and Benefits 22
3 OBJECTIVES AND ENDPOINTS 22
4 STUDY DESIGN 23
4.1 Overall Design 23
4.2 Scientific Rationale for Study Design 24
4.3 Justification for Dose 24
5 STUDY POPULATION 24
5.1 Inclusion Criteria 24
5.2 Exclusion Criteria 25
5.3 Inclusion of Vulnerable Participants 26
5.4 Lifestyle Considerations 27
5.5 Screen Failures 27
5.6 Strategies for Recruitment and Retention 27
5.6.1 Costs 28
5.6.2 Compensation 28

6 STUDY INTERVENTION 29
6.1 Study Interventions Administration 29
6.1.1 Study Intervention Description 29
6.1.2 Dosing and Administration 29
6.1.3 Drug Administration 29
6.2 Preparation/Handling/Storage/Accountability 29
6.2.1 Acquisition and Accountability 29
6.2.2 Formulation, Appearance, Packaging, and Labeling 30
6.2.3 Product Storage and Stability 30
6.2.4 Preparation 30
6.3 Measures to Minimize Bias: Randomization and Blinding 31
6.3.1 Randomization 31
6.3.2 Blinding 31
6.3.3 Scheduled Unblinding 31
6.3.4 Unscheduled Unblinding 31
6.4 Study Intervention Compliance 32
6.5 Concomitant Therapy 32
7 STUDY INTERVENTION DISCONTINUATION AND PARTICIPANT DISCONTINUATION/WITHDRAWAL 33
7.1 Discontinuation of Study Intervention 33
7.2 Participant Discontinuation/Withdrawal from the Study 33
7.3 Lost to Follow-up 33
8 STUDY ASSESSMENTS AND PROCEDURES 34
8.1 Screening Procedures 34
8.2 Efficacy Assessments 34
8.2.1 Clinical Evaluations 34
8.2.2 Biospecimen Evaluations 34
8.2.3 Correlative Studies for Research/Pharmacokinetic Studies 35
8.2.4 Samples for Genetic/Genomic Analysis 37
8.2.4.1 Description of the scope of genetic/genomic analysis 37
8.2.4.2 Description of how privacy and confidentiality of medical information/biological specimens will be maximized 37
8.2.4.3 Management of results 37

8.2.4.4 Genetic counseling 37
8.3 Safety and Other Assessments 37
8.4 Safety Definitions, Management, and Sponsor Reporting 38
8.4.1 Definitions 38
8.4.2 Protocol-Specified Exempt Events (PSEEs) 42
8.4.3 Documenting, Assessing, Recording, and Reporting Events 42
8.4.3.1 Investigator Assessment of Adverse Events 43
8.4.3.1.1 Severity Grading 43
8.4.3.1.2 Causality 44
8.4.3.2 Recording of Events 45
8.4.3.3 Investigator Reporting Responsibilities 45
8.4.3.3.1 Adverse Events 46
8.4.3.3.2 Serious Adverse Events (Expedited Reporting) 46
8.4.3.3.3 Unanticipated Problems 47
8.4.3.3.4 Pregnancy 47
8.4.3.4 Sponsor's Reporting Responsibilities 48
8.4.4 Withdrawal Criteria for an Individual Subject 48
8.4.4.1 Re-enrollment and Unplanned Procedure Repetition 49
8.4.4.2 Replacement of Withdrawn Subjects or Subjects Who Discontinue Study Agent 49
8.4.5 Additional Safety Oversight 49
8.4.5.1 Safety Review and Communications Plan 49
8.4.5.2 Sponsor Medical Monitor 49
8.4.5.3 Oversight Committees 49
8.4.5.3.1 Data and Safety Monitoring Board 49
8.4.6 Pausing Rules for the Protocol 50
8.4.6.1 Reporting a Pause 50
8.4.6.2 Resumption Following a Pause 50
8.4.6.3 Discontinuation of Study Agent 51
8.4.7 Halting Rules for the Protocol 51
8.4.7.1 Reporting a Study Halt 51
8.4.7.2 Resumption of a Halted Study 51
8.4.7.3 Discontinuation of Study Agent 52

8.5 Unanticipated Problems 52
8.5.1 Definition of Unanticipated Problems 52
8.5.2 Unanticipated Problem Reporting 52
8.5.3 NIH Intramural IRB Reporting of IND Safety Reports 52
8.6 Additional Reporting Requirements 52
8.6.1 Reporting to the NIH IRB 52
8.6.2 Reporting to the NIAID Clinical Director 52
9 STATISTICAL CONSIDERATIONS 52
9.1 Statistical Hypothesis 52
9.2 Sample Size Determination 52
9.3 Populations for Analyses 53
9.3.1 Evaluable for Toxicity 53
9.3.2 Evaluable for Objective Response 53
9.3.3 Evaluable Non-Target Disease Response 53
9.4 Statistical Analyses 54
9.4.1 General Approach 54
9.4.2 Analysis of the Primary Endpoints 54
9.4.3 Baseline Descriptive Statistics 54
9.4.4 Sub-Group Analyses 54
9.4.5 Tabulation of Individual Participant Data 54
9.4.6 Exploratory Analyses 54
10 REGULATORY AND OPERATIONAL CONSIDERATIONS 55
10.1 Informed Consent Process 55
10.1.1 Consent/Assent Procedures and Documentation 55
10.1.2 Consent for Minors When They Reach the Age of Majority 55
10.1.3 Telephone Consent 55
10.1.4 Telephone Assent 55
10.1.5 Participation of Subjects Who Are/Become Decisionally Impaired 55
10.1.1 Participation of NIH Employees 56
10.2 Study Discontinuation and Closure 56
10.3 Confidentiality and Privacy 56
10.4 Future Use of Stored Specimens and Data 57

10.5 Safety Oversight 57
10.6 Clinical Monitoring 57
10.7 Quality Assurance and Quality Control 58
10.8 Data Handling and Record Keeping 58
10.8.1 Data Collection and Management Responsibilities 58
10.8.2 Study Records Retention 59
10.9 Protocol Deviations 59
10.9.1 NIH Definition of Protocol Deviation 59
10.10 Publication and Data Sharing Policy 59
10.10.1 Human Data Sharing Plan 59
10.10.2 Genomic Data Sharing Plan 60
10.11 Collaborative Agreements 60
10.11.1 Agreement Type 60
10.12 Conflict of Interest Policy 60
11 ABBREVIATIONS 60
12 REFERENCES 63

LIST OF TABLES

Table 1. Schedule of Activities 11
Table 2: Objectives and Endpoints 22
Table 3. Participant Remuneration 28
Table 4: Vaccine Component Total Protein Concentrations, Volumes and Amounts 30
Table 5. Volumes for Specimen Collection 34
Table 6. Standard Event Recording, Assessment, and Reporting Timeframes 42
Table 7. PSEE List and Reporting 42
Table 8. Probability of meeting pausing criteria 52

LIST OF FIGURES

Figure 1. BPL-1357 Phase 1 Group Randomization Flowchart 10
Figure 2. BPL-1357 Phase 1 Study Timeline 10

Figure 3a,b,c. Serum IgG and BAL IgA titers against HA (H1, H3, H5, H7) and NA (N1, N3, N8, N9) as measured by ELISA area-under-the-curve (AUC) in mice vaccinated with intramuscular (IM) BPL-1357, intranasal (IN) BPL-1357, or mock vaccine................................16

Figure 4. Weight loss and survival following lethal challenge with H1N1 (A), H5N1 (B), H5N8 (C), H6N1 (D), H7N1 (E), H7N9 (F), and H10N7 (G) in mice vaccinated with intramuscular (IM) BPL-1357, intranasal (IN) BPL-1357, or mock vaccine..17

Figure 5. Ferret histopathology following viral challenge in ferrets vaccinated with BPL-1357 intramuscularly (IM), intranasally (IN), or mock vaccine. 18

Figure 6. Viral lung titers and immunohistochemistry following viral challenge in ferrets vaccinated with BPL-1357 intramuscularly (IM), intranasally (IN), or mock vaccine. 19

Figure 7: Drug Product Labels 30

STATEMENT OF COMPLIANCE

The trial will be carried out in accordance with International Council on Harmonisation (ICH) Good Clinical Practice (GCP) and the following:

- United States (US) Code of Federal Regulations (CFR) applicable to clinical studies (45 CFR Part 46, 21 CFR Part 50, 21 CFR Part 56, 21 CFR Part 312, and/or 21 CFR Part 812).

NIH-funded investigators and clinical trial site staff who are responsible for the conduct, management, or oversight of NIH-funded clinical trials have completed Human Subjects Protection and ICH GCP Training.

The protocol, informed consent form(s), recruitment materials, and all participant materials will be submitted to the institutional review board (IRB) for review and approval. Approval of both the protocol and the consent form must be obtained before any participant is enrolled. Any amendment to the protocol will require review and approval by the IRB before the changes are implemented to the study. In addition, all changes to the consent form will be IRB-approved; a determination will be made regarding whether a new consent needs to be obtained from participants who provided consent using a previously approved consent form.

1 PROTOCOL SUMMARY

1.1 SYNOPSIS

**Title:**	Randomized, Double-Blinded, Placebo-Controlled, Phase 1 Study of the Safety and Immunogenicity of BPL-1357, a BPL-Inactivated, Whole-Virus, Universal Influenza Vaccine
**Study Description:**	This is a randomized, double-blinded, placebo-controlled, single-center, phase 1 clinical trial of β-propiolactone (BPL)-inactivated quadruple influenza virus cocktail vaccine (BPL-1357) administered intramuscularly (IM) or intranasally (IN) in 2 doses 28 days apart. Participants will be randomized to one of three groups for treatment assignment. The primary hypothesis is that IN and IM BPL-1357 will be well tolerated.
**Objectives:**	Primary Objective:<br>1. To assess the safety of BPL-1357 given IM or IN, compared to placebo.<br>Secondary Objective:<br>1. To further assess the safety of BPL-1357 given IM or IN, compared to placebo.<br>2. To assess the immunogenicity of BPL-1357 given IM or IN, compared to placebo.<br>Tertiary Objective:<br>1. To characterize the systemic and mucosal humoral immune responses induced by BPL-1357 given IM or IN, compared to placebo.<br>2. To further characterize the immune response induced by BPL-1357 given IM or IN through variable, diversity, and joining (VDJ) gene repertoire analysis, cytokine analysis, cytometry, transcriptomics, and assessment of T-cell responses.<br>3. To assess the rates of influenza disease among groups given IM or IN BPL-1357 compared to placebo.
**Endpoints:**	Primary Endpoints:<br>1. Type and severity (by grading) of adverse events (AEs) through vaccine 2 (V_2) day 28 (D28) [28 days after vaccine dose 2].<br>2. Type of serious adverse events (SAEs) through V_2D28 [28 days after vaccine dose 2].<br>Secondary Endpoints:<br>1. Safety<br>a. Type and severity (by grading) of AEs through V_2D182 [182 days after vaccine dose 2].

b. Type of SAEs through V_2D182 [182 days after vaccine dose 2].

2. Immunogenicity

   a. Antibodies against H1, H3, H5, and H7 head and stalk as measured by hemagglutination inhibition (HAI) or enzyme-linked immunosorbent assay (ELISA) from blood and mucosal samples at V_2D28.

   b. Antibodies against N1, N3, N8, and N9 as measured by neuraminidase inhibition (NAI) or ELISA from blood and mucosal samples at V_2D28.

Tertiary Endpoints:

1. Additional antibody titer characterization via:
   a. Antibodies against H1, H3, H5, and H7 head and stalk as measured by HAI or ELISA from blood and mucosal samples at V_1D7, V_1D14, V_1D28, V_2D7, V_2D14, V_2D56, and V_2D182.
   b. Antibodies against N1, N3, N8, and N9 as measured by NAI or ELISA from blood and mucosal samples at V_1D7, V_1D14, V_1D28, V_2D7, V_2D14, V_2D56, and V_2D182.
2. Additional immune response characterization via:
   a. VDJ gene repertoire analysis.
   b. Cytokine analysis.
   c. Flow cytometric phenotyping of lymphocytes.
   d. Transcriptomic gene expression.
   e. T-cell responses.
3. Influenza disease

**Study Population:**	Healthy volunteers, male and female, ≥ 18 to ≤ 55 years of age. The target sample size is 45 participants with an accrual ceiling of 100 participants.
**Phase:**	Phase 1
**Description of Sites/Facilities Enrolling Participants:**	National Institutes of Health (NIH) Clinical Center (CC)
**Description of Study Intervention:**	Two-doses 28 days apart of BPL-1357 and/or normal saline placebo, according to randomized (1:1:1) group assignment: Group A will receive two-doses IM BPL-1357 plus two-doses IN placebo; Group B will receive two-doses IM placebo plus two-doses IN BPL-1357; and Group C will receive two-doses IM placebo and two-doses IN placebo.
**Study Duration:**	12 months

Participant Duration: 7 months

1.2 SCHEMA

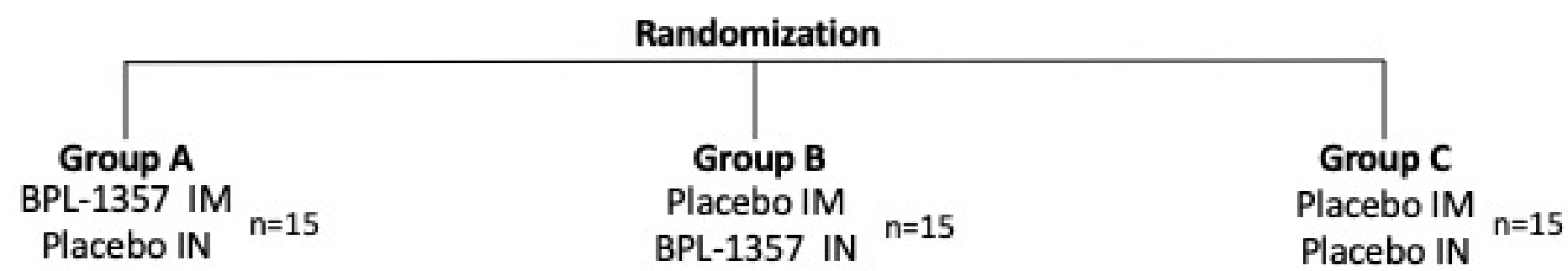

Figure 1. BPL-1357 Phase 1 Group Randomization Flowchart

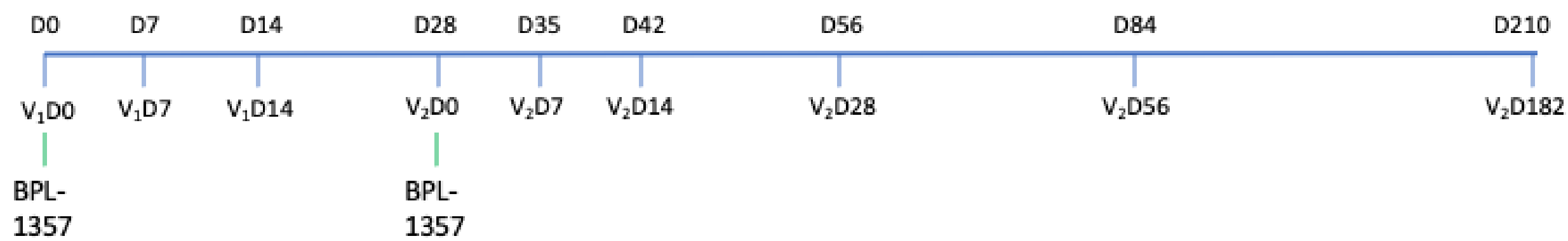

V1=Vaccine dose 1, V2=Vaccine dose 2. D=Day

Figure 2. BPL-1357 Phase 1 Study Timeline

1.3 SCHEDULE OF ACTIVITIES

Table 1. Schedule of Activities

Procedure/Evaluation	Study Visit (window, in days)								
	V_1D0	V_1D7 (± 1)	V_1D14 (± 2)	V_2D0 (± 2)	V_2D7 (± 2)	V_2D14 (± 2)	V_2D28 (± 7)	V_2D56 (± 7)	V_2D182 (± 7)
Written informed consent	X[a]								
Randomization	X[a]								
Medical/Medication history	X[a]	X	X	X[a]	X	X	X	X	X
Clinician assessment and PE	X[a]	X	X	X[a]	X	X	X	X	X
Vaccine injection-site and AE assessment		X	X	X[a]	X	X			
Survey distribution[b]	X			X					
Survey review		X			X				
Vital signs[c]	X[a]	X	X	X[a]	X	X	X	X	X
Urine pregnancy test[d]	X[a]			X[a]					
Urine drug test	X[a]								
Nasal SAM	X[a]	X	X	X[a]	X	X	X	X	X
Nasal cytology brushing		X[e]			X[e]				
Vaccination	X[f]			X[f]					
CBC + diff	X[a]	X	X	X[a]	X	X	X	X	X
Acute care, mineral, and hepatic panels	X[a]	X	X	X[a]	X	X	X	X	X
LDH, uric acid, creatine kinase, and total protein	X[a]	X	X	X[a]	X	X	X	X	X
HLA testing	X[a]								
Serum collection	X[a]	X	X	X[a]	X	X	X	X	X
Whole blood collection (PBMC)	X[a]	X	X	X[a]	X	X	X	X	X
Whole blood collection (PAXgene)	X[a]	X		X[a]	X				

AE = adverse event; CBC + diff = complete blood count with differential; D = day; HLA = human leukocyte antigen; LDH = lactate dehydrogenase; PBMC = peripheral blood mononuclear cells; PE = physical exam; SAM = synthetic absorptive matrices; X = to be performed.
Notes:

- Screening will be performed under NIH screening protocol 11-I-0183.

- If medically necessary (e.g., if a safety condition arises), the participant may be asked to return for an unscheduled interim visit for AE assessment and other safety assessments as appropriate.
- Acute care panel includes Sodium (NA), Potassium (K), Chloride (CL) Total CO2 (Bicarbonate), Creatinine, Glucose, Urea nitrogen, eGFR, Anion Gap
- Mineral panel includes Albumin, Calcium, Magnesium (Mg), and Phosphorus.
- Hepatic panel includes Alkaline Phosphatase, ALT/GPT, AST/GOT, Total Bilirubin, Direct Bilirubin

[a] Complete prior to vaccine administration.

[b] Survey (paper or electronic) will be completed each evening for 7 evenings starting V_1D0 and for another 7 evenings starting V_2D0 to capture temperature and any local symptoms (e.g. redness, swelling, and pain) or general symptoms (e.g., fever, chills, headache, myalgia, arthralgia, fatigue, nausea, vomiting).

[c] Prior to vital sign collection, participant must be sitting for a minimum of 5 minutes; vital signs include blood pressure, mean arterial pressure, heart rate, respiratory rate, temperature, weight, and pulse oximetry. Height will be performed once on V_1D0.

[d] Females of childbearing potential only.

[e] To be collected after nasal SAM is completed.

[f] Post-vaccination monitoring: Vital signs at 30 minutes, 60 minutes, and 120 minutes post-vaccination (± 15 minutes); and vaccine injection-site and AE assessment at 60 minutes and 120 minutes (± 15 minutes).

2 INTRODUCTION

2.1 STUDY RATIONALE

Since the start of 20th century, humanity has experienced five waves of influenza pandemics. The large diversity of influenza viruses in animal reservoirs portends a constant risk of the next inevitable pandemic. Current influenza vaccines are designed to protect against seasonal influenza but leave individuals largely susceptible against heterologous influenza viruses. The development of a new vaccine which can greatly expand the breadth of immunity to future pandemic strains would have the potential to save many lives and decrease global morbidity by a significant amount.

2.2 BACKGROUND

Influenza A virus infections, including annual influenza epidemics of continually drifting H3N2 and H1N1 strains, ongoing zoonotic infections with animal-derived influenza viruses (e.g. H5N1 or H7N9), and unpredictable pandemics, all make influenza a major health threat and the control of influenza a global public health priority. Globally, influenza results in 3 to 5 million severe illnesses and up to 500,000 deaths annually[1,2]. Influenza pandemics - in which novel influenza A viruses (IAVs) unpredictably emerge and against which most humans lack protective immunity - can have even larger global impacts[3]. The 1918 influenza pandemic resulted in at least 50 million deaths[4]. Current vaccine strategies rely on matching circulating viruses with vaccine antigens and have a less than optimal effectiveness. This approach will not work in a pandemic because current manufacturing techniques require months to produce enough seasonal vaccine and even with matching vaccine strains to circulating strains during the season, effectiveness is 60%.[5] Because these vaccines are unlikely to protect against an antigenically divergent strain or a new pandemic virus with a novel hemagglutinin (HA) subtype[6], there is a critical need for influenza vaccines that protect against all IAVs, a so-called “universal” vaccine[7]. Recently, there has been a large focus, both governmental and nongovernmental, to develop new generations of broadly protective or “universal” influenza vaccines[8].

IAVs are enveloped, negative-sense, single-stranded ribonucleic acid (RNA) viruses with segmented genomes. In addition to humans, IAVs infect large numbers of warm-blooded animal hosts including over 100 avian species and many mammalian species, with numerous species of wild aquatic birds serving as the major natural reservoir[4]. IAVs express three surface proteins—HA, neuraminidase (NA), and matrix 2 (M2). IAVs are subtyped by antigenic characterization of the HA and NA glycoproteins. Sixteen HA and 9 NA subtypes are consistently found in avian hosts in various combinations (e.g., H1N1 or H3N2), and these wild bird viruses are thought to be the ultimate source of previous human pandemic influenza viruses. IAV genome segmentation allows for viral reassortment, and since HA and NA are encoded on separate gene segments, novel IAVs of any subtype can be generated following mixed infections in any host, a process that has been termed “antigenic shift.” IAVs are also evolutionarily dynamic RNA viruses with high mutation rates. Mutations that change amino acids in the antigenic portions of HA and NA proteins may allow strains to evade population immunity (“antigenic drift”). Unfortunately, despite enhanced surveillance and research on host switch events, future pandemics cannot be predicted, including when and where a pandemic virus strain will emerge,

what the viral subtype will be, or how pathogenic it will be in humans. Severe human infections with animal-origin IAVs have also been observed, including recent human infections with avian H5N1 and H7N9 viruses[9,10].

Therefore, there are two major issues that with existing influenza vaccines that need to be improved upon: breadth of protection (including against IAVs not currently endemic in the human population) and vaccine effectiveness. It is not well understood why influenza vaccines have not been able to improve their efficacy rate since their development nearly 70 years ago,[2,11] but there has been recent interest among the scientific community in the otherwise neglected role of mucosal immunity. While intramuscular vaccines primarily induce immunoglobulin G (IgG) and not immunoglobulin A (IgA) responses, IgA is much more prevalent in upper respiratory mucosa compared to IgG, which may play a larger role in the lower respiratory tract.[12] Mucosal influenza vaccination has been shown to be effective in animal studies,[13] but outside of live attenuated vaccines, has not been well studied in humans.

Laboratory of Infectious Diseases (LID) Viral Pathogenesis and Evolution Section (VPES) has completed pre-clinical development of a novel universal influenza vaccine candidate which offers broad protective efficacy against a wide variety of IAV subtypes and strains in mice and ferrets. Preclinical development and Good Manufacturing Practice (GMP) production of the vaccine have been facilitated by a gift fund from the Bill & Melinda Gates Foundation (BMGF) to National Institute of Allergy and Infectious Diseases (NIAID).

The vaccine is a non-infectious, chemically inactivated, whole-virus vaccine consisting of four low-pathogenicity avian IAVs (H1N9, H3N8, H5N1, H7N3) representing 4 different HA and 4 different NA subtypes. The vaccine has been shown to provide broad protection in mice and ferrets with either IM (adjuvanted with AddaVax™, a squalene-based oil-in-water emulsion) or IN dose administration.

In mice, BPL-1357 was immunogenic. Vaccinated animals developed detectable antibodies against the vaccine antigen HA heads, HA stalks, and against NA (Figure 3d,e,f. Serum IgG and BAL IgA titers against HA (H1, H3, H5, H7) and NA (N1, N3, N8, N9) as measured by ELISA area-under-the-curve (AUC) in mice vaccinated with intramuscular (IM) BPL-1357, intranasal (IN) BPL-1357, or mock vaccine.). IM vaccinated animals generally had more robust serum titers while IN vaccinated animals had more robust mucosal titers. However, both routes of administration were protective after infectious challenge. Protection was observed in vaccinated animals subsequently challenged with lethal doses of multiple strains of IAV (Figure 4. Weight loss and survival following lethal challenge with H1N1 (A), H5N1 (B), H5N8 (C), H6N1 (D), H7N1 (E), H7N9 (F), and H10N7 (G) in mice vaccinated with intramuscular (IM) BPL-1357, intranasal (IN) BPL-1357, or mock vaccine.), including mismatched (Figure 4a, 4d) and heterosubtypic viruses (Figure 4g) compared to mock-vaccinated animals (either phosphate-buffered saline [PBS] IN or PBS+adjuvant IM). Vaccinated mice showed improved survival and decreased weight loss compared to control animals in the face of lethal challenge. These observations demonstrated very broad, potent, protective efficacy, including protection against lethal infections of completely novel IAVs.

In ferrets, broad, potent, protective efficacy was observed against mismatched and completely heterosubtypic challenge with a variety of influenza virus challenge strains. Vaccinated ferrets showed marked abrogation of pneumonia as assessed by histopathology (Figure 5) and immunohistochemistry (Figure 6) for viral antigen as well as reduced viral loads (Figure 6). Lung sections of unvaccinated ferrets showed marked pathological changes involving over 50% of the lung parenchyma, including multifocal, moderate-to-severe, necrotizing bronchitis and bronchiolitis, along with moderate-to-severe alveolitis with a mixed inflammatory cell infiltrate, and focal pulmonary edema and fibrinous exudates. In a separate experiment (data not shown) with ferrets to determine the effect of pre-existing immunity on vaccine response, animals were exposed to either seasonal H1N1 and H3N2 viruses prior to vaccination. Subsequent vaccine-associated protective efficacy was still demonstrated after challenge with heterosubtypic virus (H3N2 for animals initially exposed to H1N1, and H1N1 for animals initially exposed to H3N2). These data suggest that protective efficacy is not negatively impacted by pre-existing immunity from prior influenza virus exposure.

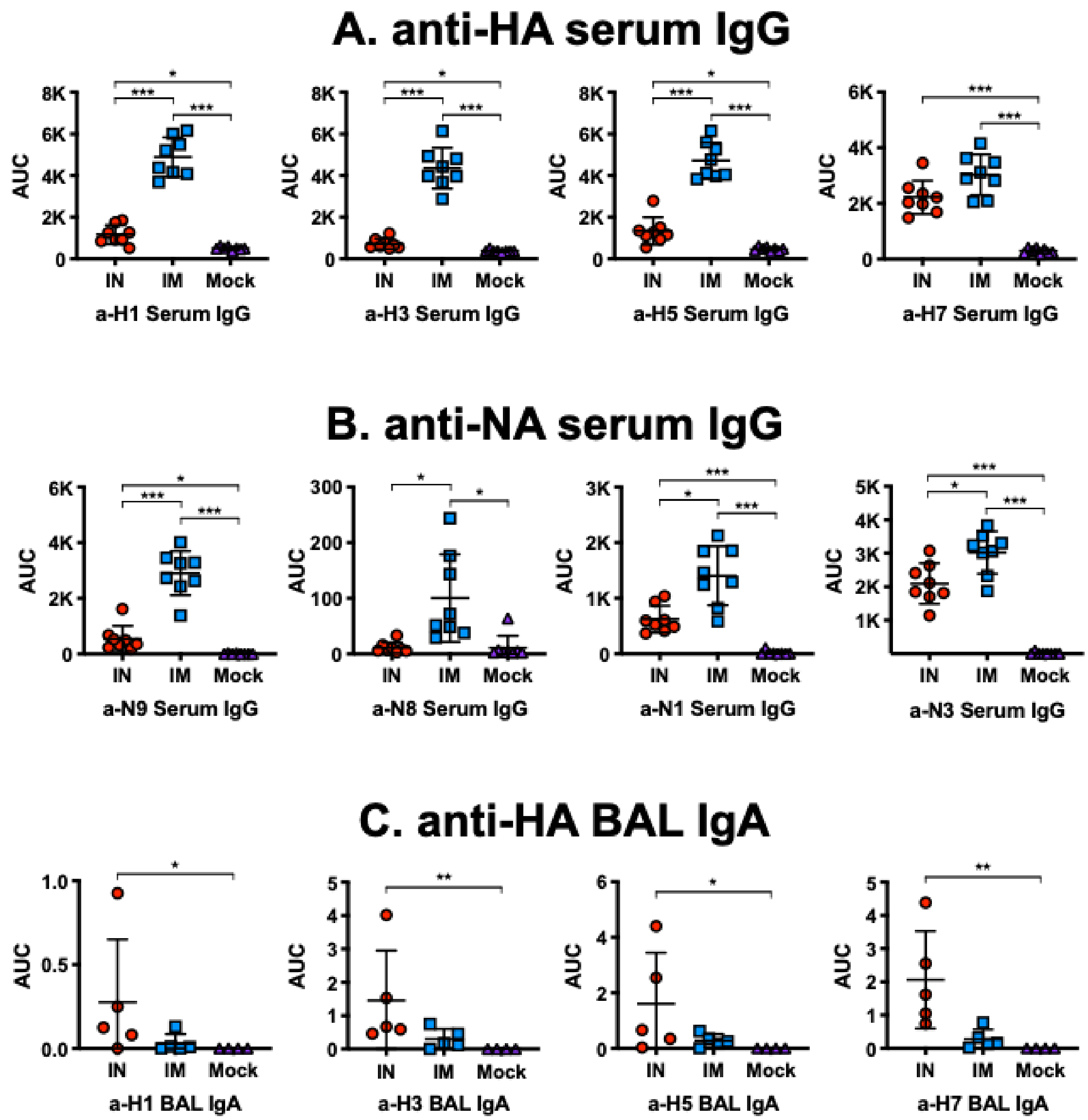

Figure 3a,b,c. Serum IgG and BAL IgA titers against HA (H1, H3, H5, H7) and NA (N1, N3, N8, N9) as measured by ELISA area-under-the-curve (AUC) in mice vaccinated with intramuscular (IM) BPL-1357, intranasal (IN) BPL-1357, or mock vaccine.

* denotes p<0.05. ** denotes p<0.01. *** denotes p<0.001.

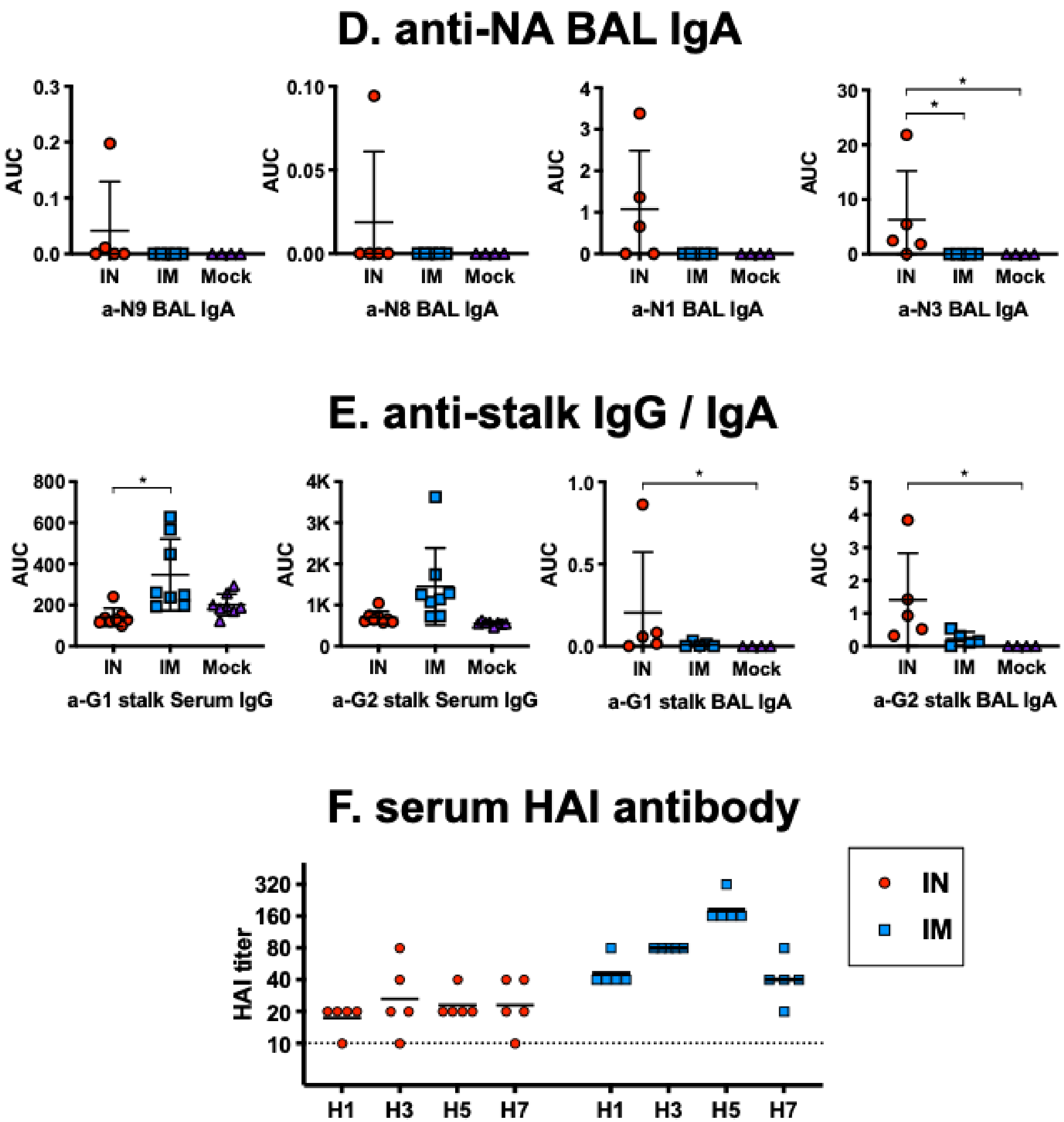

Figure 3d,e,f. Serum IgG and BAL IgA titers against HA (H1, H3, H5, H7) and NA (N1, N3, N8, N9) as measured by ELISA area-under-the-curve (AUC) in mice vaccinated with intramuscular (IM) BPL-1357, intranasal (IN) BPL-1357, or mock vaccine. Serum hemagglutinin inhibiting antibody titers also provided.

* denotes $p<0.05$. ** denotes $p<0.01$. *** denotes $p<0.001$.

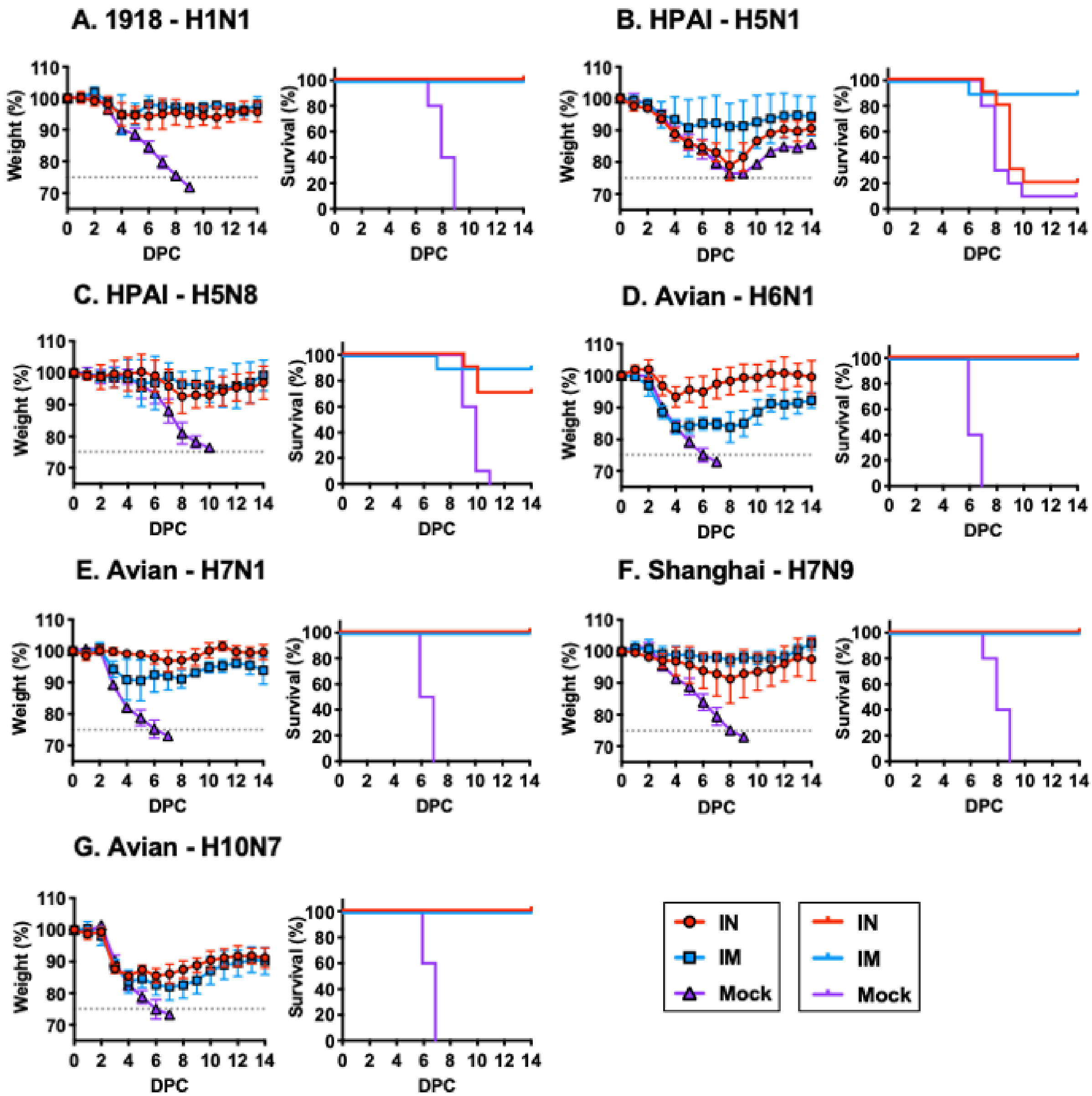

Figure 4. Weight loss and survival following lethal challenge with H1N1 (A), H5N1 (B), H5N8 (C), H6N1 (D), H7N1 (E), H7N9 (F), and H10N7 (G) in mice vaccinated with intramuscular (IM) BPL-1357, intranasal (IN) BPL-1357, or mock vaccine.

DPC=days post challenge.

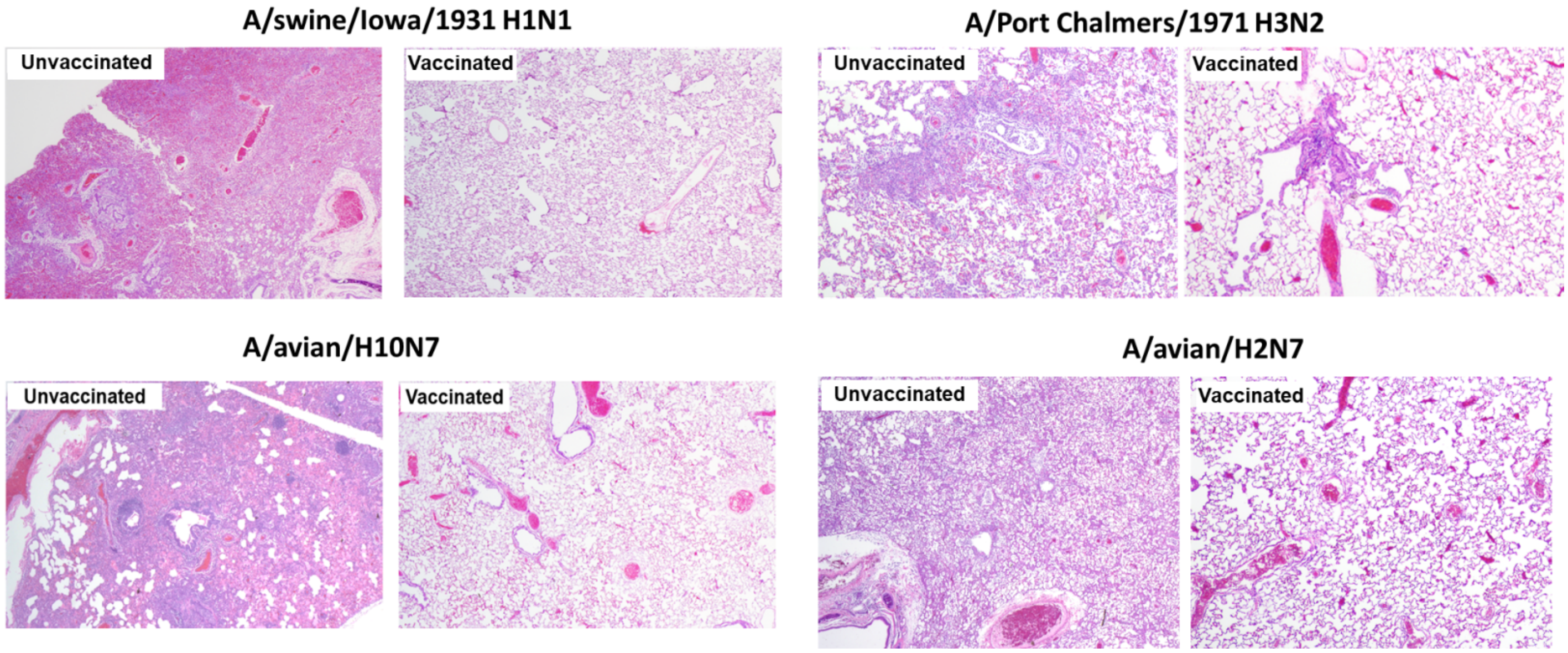

Figure 5. Ferret histopathology following viral challenge in ferrets vaccinated with BPL-1357 intramuscularly (IM), intranasally (IN), or mock vaccine.

$TCID_{50}$ refers to median tissue culture infectious disease and is defined as the concentration at which 50% of inoculated cells are infected. Representative images from both IM and IN vaccinated animals.

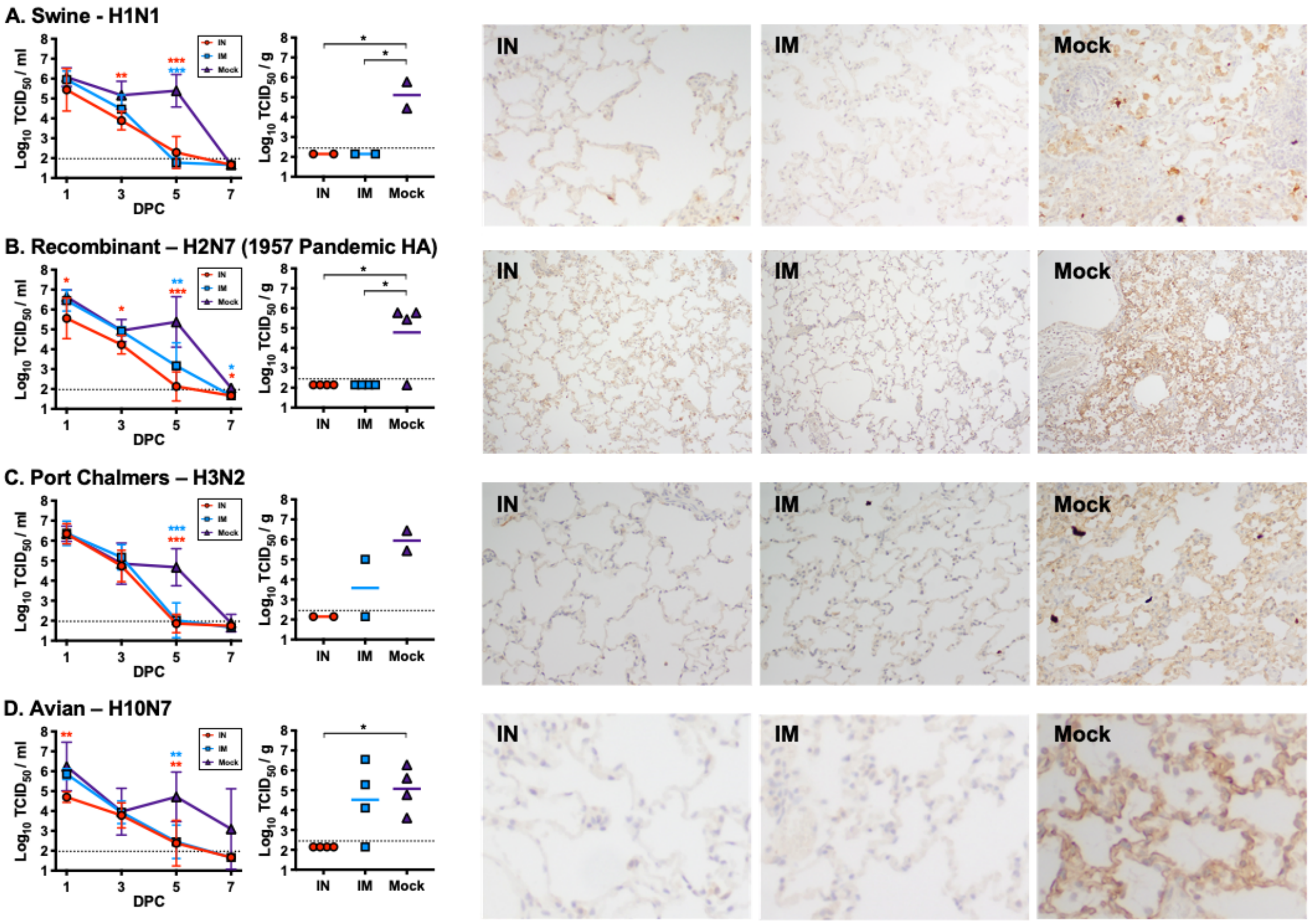

Figure 6. Viral lung titers and immunohistochemistry following viral challenge in ferrets vaccinated with BPL-1357 intramuscularly (IM), intranasally (IN), or mock vaccine.

$TCID_{50}$ refers to median tissue culture infectious disease and is defined as the concentration at which 50% of inoculated cells are infected. Dotted line reflects lower limit of detection.

In light of the encouraging mouse and ferret data, a phase 1 clinical trial was designed to test the safety of the vaccine in humans, given either IM or IN.

2.3 Risk/Benefit Assessment

2.3.1 Known Potential Risks

2.3.1.1 Risks of BPL-1357 Influenza Vaccination

The risks for administration of BPL-1357 IM or IN are not well known. As an inactivated influenza vaccine, the risks of BPL-1357 IM or IN are presumably similar to other inactivated protein vaccines, such as seasonal influenza vaccines (some of which are inactivated using BPL)[14] given IM or IN, and are listed below.

Possible risks of IM BPL-1357:

- Redness, pain, swelling, and/or bruising at the injection site
- Hoarseness
- Painful, red, or itchy eyes
- Runny or stuffy nose

- Cough
- Fatigue/tiredness
- Fever
- Headache
- Muscle pain or aches
- Joint pain
- Nausea or vomiting
- Diarrhea
- Poor appetite
- Sore throat
- Chills
- Itching
- Sneezing
- Dizziness or fainting after vaccination

Rare but serious potential side effects could include:

- Guillain-Barré syndrome (GBS). The link between flu vaccine and GBS is not well established. The annual risk of GBS is 1-2 per 100,000 people in the general population. If the flu vaccine does increase risk, it likely adds a risk of 1-2 cases per million vaccinated people. It is possible BPL-1357 could have similar risk.
- Severe arm pain due to brachial neuritis. This is a rare risk with less than 1 in a million cases occurring with possible relationship to flu vaccination. The annual risk of brachial neuritis is 2-3 per 100,000 people in the population. It is possible BPL-1357 could have similar risk.
- Severe allergic reaction. The risk of severe allergic reaction is rare and anaphylaxis risk is about 1.3 in a million vaccinated people. The general lifetime risk of a severe allergic reaction is 2-3 in 100 people. It is possible BPL-1357 could have similar risk. Subjects will be monitored at least 2 hours after vaccination and provided treatment if needed.
- Serious injury or death. This is extremely rare. It is possible BPL-1357 could have similar risk.

Possible Risks of IN BPL-1357:

- Runny or stuffy nose
- Nose irritation
- Cough
- Wheezing
- Difficulty breathing
- Fatigue/tiredness
- Fever
- Chills
- Headache
- Muscle pain or aches
- Joint pain
- Nausea or vomiting

- Diarrhea
- Poor appetite
- Sore throat
- Abnormal taste
- Hoarseness
- Painful, red, or itchy eyes
- Itching
- Sneezing
- Nosebleed
- Dizziness or fainting after vaccination

Rare but serious potential side effects could include:

- GBS. The link between flu vaccine and GBS is not well established. The annual risk of GBS is 1-2 people per 100,000 in the general population. If the flu vaccine does increase risk, it likely adds a risk of 1-2 cases per million vaccinated people. It is possible BPL-1357 could have similar risk.
- Bell's palsy. The risk of Bell's palsy in patients who received the inactivated IN flu vaccine Nasalflu (Berna Biotech) is believed to be due to the vaccine adjuvant, which is not present in BPL-1357. Bell's palsy risk increased by 13 cases per 10,000 vaccinated persons. Bell's palsy has been reported in post-marketing studies of FluMist at an unknown rate. It is possible BPL-1357 could have similar risk.
- Severe allergic reaction. The risk of severe allergic reaction is is rare and anaphylaxis risk is about 1.3 in one million vaccinated people. The general lifetime risk of a severe allergic reaction is 2-3 in 100 people. It is possible BPL-1357 could have similar risk. Subjects will be monitored at least 2 hours after vaccination by trained personnel and provided treatment if needed.
- Serious injury or death. This is extremely rare with influenza vaccination. It is possible BPL-1357 could have similar risk.

Individuals with previous allergic reactions to influenza vaccines are excluded to minimize the risk of severe allergic reactions (See Exclusion Criteria).

2.3.1.2 Risks of Placebo Administration

IM Placebo Administration: IM injection of saline can cause muscle soreness and injection-site pain, redness, swelling, and bruising.

IN Placebo Administration: IN administration of normal saline can cause sneezing, coughing, nose irritation, abnormal taste, and nose bleeding.

2.3.1.3 Risks of Blood Draw

Risks of blood draw include pain, bruising, bleeding, and rarely fainting, blood clot, or infection.

2.3.1.4 Risks of Nasal Sampling

Risks of nasal sampling with synthetic absorptive matrices (SAM) or cytology brushing include local discomfort, coughing, gagging, or minor nose bleeding. Prior to nasal brushing, the nostril will be numbed using lidocaine with phenylephrine spray to minimize discomfort. Possible side effects of lidocaine with phenylephrine include lightheadedness, anxiety, dizziness, vomiting, local edema, and local erythema. There is also a very rare risk of allergic reaction to lidocaine with phenylephrine, which could involve symptoms such as itching, sneezing, hives, difficulty breathing, hypotension, or death.

2.3.1.5 Risks of Urine Drug Testing

The risks of obtaining urine samples for drug testing are minimal. However, the results will be stored in the medical record.

2.3.2 Known Potential Benefits

There is no known direct benefit to the participant. The results of this study may improve the investigators' understanding of influenza vaccines, the differences between IN and IM administration of influenza vaccines, and how those methods may protect against infection.

2.3.3 Assessment of Potential Risks and Benefits

The risks of influenza to both individual and society are significant. Administration of yearly influenza vaccine is recommended as standard-of-care to nearly all individuals to mitigate the morbidity and mortality associated with influenza, despite effectiveness ranging from only 10%-60%[2]. The potential benefit of a universal influenza vaccine justifies the potential risks described above to determine if this strategy is safe and effective. Additionally, potential risks are minimized by careful design of participant eligibility criteria and post-vaccination monitoring. The improved vaccine efficacy that could come from this and follow-up studies would be highly beneficial to worldwide public health.

3 OBJECTIVES AND ENDPOINTS

Table 2: Objectives and Endpoints

OBJECTIVES	ENDPOINTS	JUSTIFICATION FOR ENDPOINTS
Primary		
To assess the safety of BPL-1357 given IM or IN, compared to placebo.	a. Type and severity (by grading) of AEs through day V_2D28.<br>b. Type of SAEs through day V_2D28.	The standard way to determine safety is to evaluate AE incidence and severity.
Secondary		
1. To further assess the safety of BPL-1357 given IM or IN, compared to placebo.<br>2. To assess the immunogenicity of BPL-	1. Safety<br>a. Type and severity (by grading) of AEs through day V_2D182.	Analysis of antibody titers will enhance our understanding of the immune response to BPL-1357.

OBJECTIVES	ENDPOINTS	JUSTIFICATION FOR ENDPOINTS
1357 given IM or IN, compared to placebo.	b. Type of SAEs through day V_2D182.<br>2. Immunogenicity<br>c. Antibodies against H1, H3, H5, and H7 head and stalk as measured by HAI or ELISA from blood and mucosal samples at V_2D28.<br>d. Antibodies against N1, N3, N8, and N9 as measured by NAI or ELISA from blood and mucosal samples at V_2D28.	
Tertiary/Exploratory		
1. To characterize the systemic and mucosal immune responses induced by BPL-1357 given IM or IN, compared to placebo.<br>2. To further characterize the immune response induced by BPL-1357 given IM or IN through variable, diversity, and joining (VDJ) gene repertoire analysis, cytokine analysis, cytometry, transcriptomics, and assessment of T-cell responses.<br>3. To assess the rates of influenza disease among groups given IM or IN BPL-1357 compared to placebo.	1.<br>a) Antibodies against H1, H3, H5, and H7 head and stalk as measured by HAI or ELISA from blood and mucosal samples at V_1D7, V_1D14, V_1D28, V_2D7, V_2D14, V_2D56, and V_2D182.<br>b) Antibodies against N1, N3, N8, and N9 as measured by NAI or ELISA from blood and mucosal samples at V_1D7, V_1D14, V_1D28, V_2D7, V_2D14, V_2D56, and V_2D182.<br>2.<br>b. VDJ gene repertoire analysis.<br>c. Cytokine analysis.<br>d. Flow cytometric phenotyping of lymphocytes.<br>e. Transcriptomic gene expression.<br>f. T-cell responses.<br>3. Influenza disease	Additional analysis of antibody titers will further enhance our understanding of the immune response to BPL-1357.<br>VDJ gene repertoire analysis, cytokine analysis, flow cytometry, transcriptomic analysis, and analysis of T-cell activity may help identify other possible correlates of protection for influenza after vaccination, such as innate and other cellular responses.<br>Rates of influenza disease among groups may provide additional data regarding vaccine-associated immunity

4 STUDY DESIGN

4.1 OVERALL DESIGN

This is a randomized, placebo-controlled, single-center, phase 1 clinical trial of BPL-1357 administered via IM or IN routes. The primary safety hypothesis is that BPL-1357 given IN or IM will be well-tolerated with minor side effects.
Participants will be randomized 1:1:1 to one of three groups:

- Group A will receive IM BPL-1357 plus IN placebo.
- Group B will receive IM placebo plus IN BPL-1357.
- Group C will receive IM and IN placebo.

This study will test a single dose of vaccine given at two timepoints 28 days apart. Group C will receive IM and IN placebo.

For each participant, the duration of study participation will be about 7 months (Figure 2). Participants will be followed regularly for 210 days to assess for side effects and to collect nasal mucosal and blood samples to evaluate immunity and characterize immune response. Participants will receive the intervention on 'vaccine 1 day 0' (V_1D0) and will return for follow-up on vaccine 1 day 7±1 14±2, and 28±2. They will receive the second dose of vaccine/placebo on 'day 28', referred to as 'vaccine 2 day 0' (V_2D0). Subsequent visits will be scheduled with respect to the timing of V_2D0 (i.e. V_2D7 will be scheduled for 7 days after V_2D0 and not 35 days after V_1D0, if there is disagreement between the two). Participants will return for follow-up on vaccine 2 day 7±2, 14±2, 28±7, 56±7, and 182±7 (Table 1). Participants will also complete a survey questionnaire at home for 7 days after each vaccine dose to further evaluate safety (APPENDIX A). Participants will be sent home with a thermometer to facilitate at home temperature monitoring and instructions will be provided (APPENDIX B). Participants who develop symptoms concerning for influenza-like illness (ILI) (including but not limited to rhinorrhea, sore throat, change in smell or taste, cough, wheezing, dyspnea, fatigue, weakness, malaise, dizziness, fever, chills, headache, myalgias, arthralgias, diarrhea) will contact the study team regarding evaluation. Those with confirmed influenza-like illness by the study team who are unable to be evaluated expeditiously at NIH will be instructed to perform an at-home nasal swab and/or nasal SAM and send the specimens in for testing. Collection and packing materials will be provided to participants either in person or will be mailed to participants. Detailed instructions for sample collection and packaging will be given to participants (APPENDIX C).

4.2 SCIENTIFIC RATIONALE FOR STUDY DESIGN

Randomization will be used to minimize bias in the assignment of participants to treatment groups, to increase the likelihood that known and unknown participant attributes (e.g., demographic and baseline characteristics) are evenly balanced across treatment groups, and to enhance the validity of statistical comparisons across treatment groups. Likewise, use of double blinding and placebo-administered controls helps to minimize bias and accurately determine the true effects of the intervention.

4.3 JUSTIFICATION FOR DOSE

The vaccine dose targets a dose comparable (but lower) by body weight to the dosing given in rabbits. In preclinical testing, rabbits were dosed at a dose to body mass ratio of 0.008993mg/kg per each virus component without adverse event. Adjusted for a 30 kg individual, this would correspond to a dose of each component of 269.79 ug which is higher than the dose proposed for the clinical study. Using 0.25 ml of each component for a total of 1 ml of vaccine, we achieve doses for each individual component ranging from 90.9025 ug to 133.61 ug, as shown in Table 4.

5 STUDY POPULATION

5.1 INCLUSION CRITERIA

Individuals must meet all of the following criteria to be eligible for study participation:

1. ≥ 18 and ≤ 55 years of age.
2. Non-smoker (tobacco and cannabis) and does not use vape or e-cigarette products.
3. Has not received influenza vaccination of any type in the 8 weeks prior to enrollment and willing to not receive influenza vaccination of any type until after the V_2D56 visit. Participants who enroll in our study will be informed of the Centers for Disease Control and Prevention (CDC) recommendation to receive seasonal influenza vaccination annually.
4. Has not received Coronavirus Disease 19 (COVID-19) vaccination of any type in the 4 weeks prior to enrollment and willing to not receive COVID-19 vaccination of any type until after the V_2D28 visit. Participants who enroll in our study who are interested in getting a COVID-19 vaccination will be counselled to receive it prior to enrolling into our study.
5. A female participant is eligible for this study if she is not pregnant or breastfeeding and meets one of the following criteria, beginning at least 4 weeks prior to enrollment through the end of the study period (V_2D182):
    a. Is infertile, including postmenopausal status (as defined by no menses for ≥ 1 year) or history of hysterectomy or bilateral oophorectomy.
    b. Agrees to practice abstinence.
    c. Agrees that, with heterosexual intercourse with a fertile male partner, she will use an acceptable form of contraception and her male partner will use a condom with spermicide. Acceptable effective methods of female contraception include the following: bilateral tubal ligation, implant of levonorgestrel, injectable progestogen, intrauterine device, oral contraceptive pills, and diaphragm with spermicide.
6. Able to provide informed consent.
7. Able to speak English.
8. Human immunodeficiency virus (HIV) uninfected with a negative test within 60 days of enrollment.

9. Does not use IN medications (including but not limited to nasal sprays, sinus rinses), over-the-counter medications (including but not limited to aspirin, decongestants, antihistamines, and other nonsteroidal anti-inflammatory drugs), and herbal medications (including but not limited to herbal tea or St. John's Wort) within 14 days prior to study enrollment, and agrees not to use these mediations through the final study visit, unless approved by the investigator.
10. Agrees not to donate blood or blood products from 3 months prior to enrollment through the final study visit.

5.2 Exclusion Criteria

Individuals meeting any of the following criteria will be excluded from study participation:

1. Presence of self-reported or medically documented significant medical condition including but not limited to:
    a. Chronic pulmonary disease (e.g., asthma, emphysema).
    b. Chronic cardiovascular disease (e.g., cardiomyopathy, congestive heart failure, cardiac surgery, ischemic heart disease, known anatomic defects).
    c. Chronic medical conditions requiring close medical follow-up or hospitalization during the past 5 years (e.g., insulin-dependent diabetes mellitus, renal dysfunction, hemoglobinopathies).
    d. Immunosuppression, immune deficiency, or ongoing malignancy.
    e. Neurological and neurodevelopmental conditions (e.g., Bell's palsy, cerebral palsy, epilepsy, stroke, seizures).
    f. Postinfectious or postvaccine neurological sequelae including GBS.
    g. Body mass index (BMI) $\leq$ 18 and $\geq$ 35.
2. Acute illness within 7 days prior to enrollment.
3. Individuals who have grade 2 or above clinically significant laboratory values outside the limits thus specified by normal laboratory parameters.
4. Known allergy to influenza vaccination or excipients contained in the influenza vaccine used.
5. Known allergy to lidocaine or phenylephrine.
6. Receipt of blood or blood products (including immunoglobulins) within 3 months prior to enrollment.
7. Receipt of any unlicensed drug within 3 months or 5.5 half-lives (whichever is greater) prior to enrollment.
8. Receipt of any unlicensed vaccine within 6 months prior to enrollment, not including COVID-19 vaccines under Emergency Use Authorization.
9. Self-reported or known history of alcoholism or drug abuse within 6 months prior to enrollment, or positive urine test for drugs of abuse (i.e., amphetamines, cocaine

metabolites, benzodiazepines, opiates, or tetrahydrocannabinol) prior to vaccination on V_1D_0.

10. Self-reported or known history of psychiatric or psychological issues that require treatment and are deemed by the principal investigator (PI) to be a contraindication to protocol participation.
11. History of angioedema or anaphylaxis.
12. History of SARS-COV-2 infection with residual or ongoing symptoms.
13. Any condition or event that, in the judgment of the PI, is a contraindication to protocol participation or impairs the participant's ability to give informed consent.

Co-enrollment Guidelines: Co-enrollment in other trials is restricted but may take place with the approval of the PI and after study staff notification. Study staff should be notified of co-enrollment on any other protocol as it may require the approval of the PI or sponsor medical monitor (SMM).

5.3 INCLUSION OF VULNERABLE PARTICIPANTS

Pregnant women: In this study, a novel vaccine candidate will be administered to the participants, and the effects of BPL-1357 on pregnant women or the developing human fetus are unknown. Therefore, pregnant women will be excluded as the risk to these individuals may be increased.

Children: Individuals younger than 18 years of age will be excluded from the study because there are insufficient data regarding dosing or AEs available in adults to judge the potential risk in children.

Adults over 55 years: Older individuals have diminished responses to standard quadrivalent vaccination and may need higher doses. They may also have a higher rate of diagnosed or undiagnosed medical problems which may confound a phase 1 clinical trial assessing safety. Therefore, only adults aged 55 years and younger are included in this study.

Decisionally Impaired Adults: Adults who are unable to consent are not eligible for this study. Loss of ability to consent will be handled according to section 10.1.5.

NIH Employees: NIH employees and members of their immediate families may participate in this protocol as this population meets the study entry criteria. Neither participation nor refusal to participate as a subject in the research will have an effect, either beneficial or adverse, on the participant's employment or position at NIH.
Every effort will be made to protect participant information, but such information may be available in medical records and may be available to authorized users outside of the study team in both an identifiable an unidentifiable manner.

The *NIH Frequently Asked Questions (FAQs) for Staff Who are Considering Participation in NIH Research* will be made available.

- The employee participant's privacy and confidentiality will be preserved in accordance with NIH CC and NIAID policies, which define the scope and limitations of the protections.
- The importance of maintaining confidentiality when obtaining potentially sensitive and private information from co-workers or subordinates will be reviewed with the study staff at least annually and more often if warranted.

Non-English Speakers: Non-english speaking participants will be excluded from the study due to unavailability of the Redcap questionnaire in languages other than English

5.4 Lifestyle Considerations

Not applicable.

5.5 Screen Failures

Screen failures are defined as participants who consent to be screened for participation in this clinical trial but do not meet eligibility criteria to be entered in the study. Screening will take place on a separate screening protocol 11-I-0183 ("Screening for LID Clinical Studies Unit Healthy Volunteer Protocols"). Therefore, no screen failure will be consented for this protocol.

5.6 Strategies for Recruitment and Retention

Healthy volunteers will be carefully selected using the inclusion and exclusion criteria described in sections 5.1 and 5.2 to select the optimum participants for completing the study objectives and minimize the risk of AEs. Participant selection will not be limited based on sex, race, or ethnicity. The target sample size is 45 participants with an accrual ceiling of 100 participants. Potential study participants will be identified and recruited from the community through multiple methodologies including local advertisement, direct contact of prior study participants, and word-of-mouth advertisement. Participants will be recruited through the NIH screening study 11-I-0183, "Screening for LID Clinical Studies Unit Healthy Volunteer Protocols." Individuals will be carefully screened and evaluated under that protocol. Study team members will record histories, perform physicals, review labs, and examine electrocardiograms (EKGs), and individuals who meet the study eligibility criteria will be contacted by phone or email and given the opportunity to be enrolled into this study. Retention is encouraged through increasing participant remuneration at later visits.

If a participant has completed the screening study 11-I-0183 more than 60 days prior to enrolling in this study, they will be asked to come to the NIH CC for another screening visit under 11-I-0183 to repeat HIV testing and complete any laboratory or other testing as deemed necessary by the investigator to ensure that it remains safe for the participant to take part in this study. All eligible participants will be consented and enrolled for this study only after completion of all necessary screening studies under protocol 11-I-0183.

5.6.1 Costs

There are no costs to participation in this study.

5.6.2 Compensation

Participants will be remunerated according to Table 3. Participant Remuneration.

Table 3. Participant Remuneration

Study Visit	Amount
**V_1D0**	$225
**V_1D7 (± 1 days)**	$150
**V_1D14 (± 2 days)**	$150
**V_2D0 (± 2 days)**	$225
**V_2D7 (± 2 days)**	$150
**V_2D14 (± 2 days)**	$150
**V_2D28 (± 7 days)**	$200
**V_2D56 (± 7 days)**	$200
**V_2D182 (± 7 days)**	$250
Expected total for completion of ALL study visits	*$1700*
Study-requested interim visits	$75

Remuneration will be given according to the number of visits participants complete. Participants will only be remunerated for interim visits requested by the investigators if medically necessary. Remuneration will not be provided for interim visits requested by the participant. Remuneration will be provided to the participants in 3 intervals: after **V_2D0**, **V_2D28**, and **V_2D182** via direct deposit or check.

Reimbursement will not be provided for travel and meal vouchers will not be issued. Renumeration will not be provided for participants who are determined to be ineligible by the urine drug screen on **V_1D0.**

6 STUDY INTERVENTION

6.1 STUDY INTERVENTIONS ADMINISTRATION

6.1.1 Study Intervention Description

BPL-1357 contains 4 whole inactivated avian influenza viruses: A/Environment/Maryland/261/2006 H7N3, A/Mallard/Maryland/802/2007 H5N1, A/Pintail/Ohio/339/1987 H3N8, and A/Mallard/Ohio/265/1987 H1N9. It will be administered either IM or IN depending on the assigned treatment group. The placebo will be 1 mL normal saline in a syringe administered IM or 0.5 mL normal saline in two syringes administered IN, depending on the assigned treatment group.

6.1.2 Dosing and Administration

A single study agent dose of 1 mL will be used either IM or given in two doses of 0.5 mL IN in each nare. Participants will be randomized to receive IM and IN doses of study agents twice 28 days apart as follows:

- Group A: IM BPL-1357 and IN placebo.
- Group B: IM placebo and IN BPL-1357.
- Group C: IM and IN placebo.

6.1.3 Drug Administration

Participants will be administered study agent via IM and IN routes by study staff or personnel trained to administer the vaccine.

Participants will receive IM BPL-1357 or placebo in the arm per standard methods. A 1- to 1.5-inch 22G-25G needle as appropriate will be attached to the syringe and 1 dose will be administered in the deltoid muscle of the upper arm. Doses will be given within 4 hours of preparation. Used syringes will be disposed of in biohazard trash, which is handled according to site biohazard trash guidelines.

Participants will receive IN BPL-1357 or placebo using a syringe with nasal atomizer. Participants will be asked to lie down with their heads in a neutral to slightly tilted back position. Two MAD Nasal (Teleflex Incorporated) intranasal mucosal atomization devices will be attached to 2 syringes and 1 half-dose will be administered in each nostril.

6.2 PREPARATION/HANDLING/STORAGE/ACCOUNTABILITY

6.2.1 Acquisition and Accountability

BPL-1357 and placebo will be maintained by the NIH CC Pharmacy. Unused BPL-1357 and placebo will be disposed of and accounted for according to standard policies of the NIH CC Pharmacy. Records will be kept by the pharmacy for performing drug accountability monitoring.

6.2.2 Formulation, Appearance, Packaging, and Labeling

BPL-1357 is a non-infectious, chemically inactivated, whole virus vaccine consisting of four low pathogenicity avian influenza A viruses (H1N9, H3N8, H5N1, H7N3). The products will be packaged and labeled individiually with investigational labels prior to mixing by the pharmacy, as shown below.

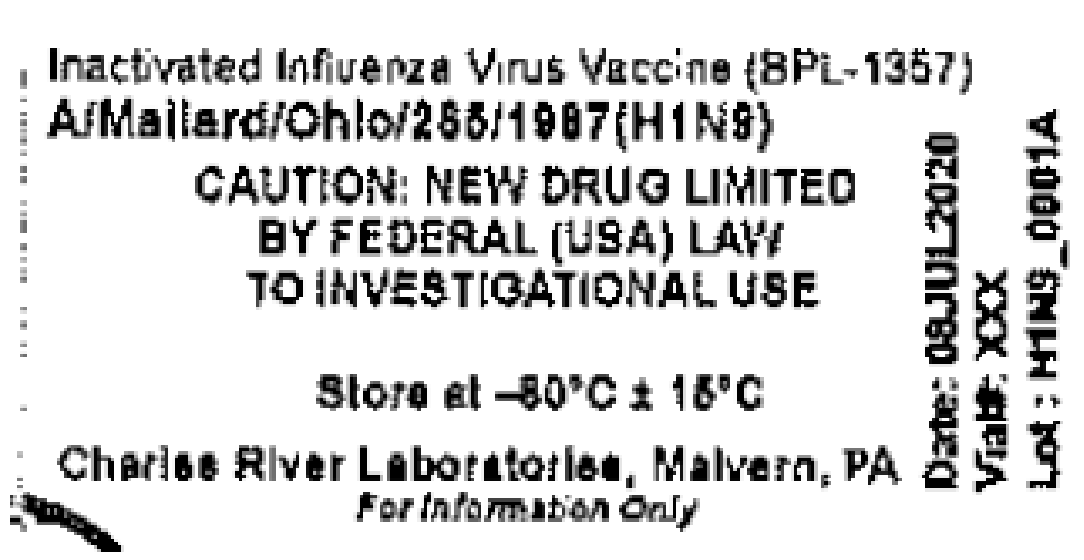

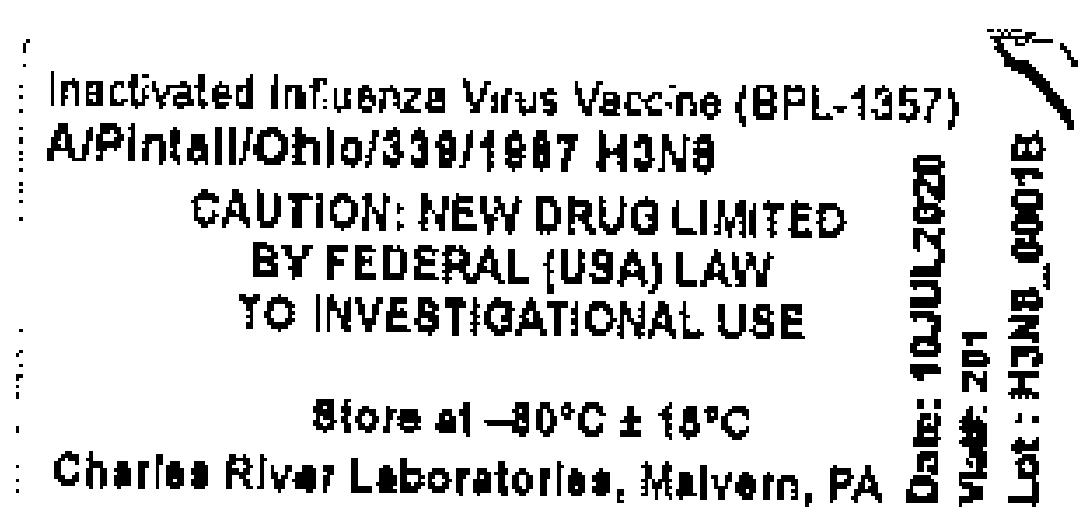

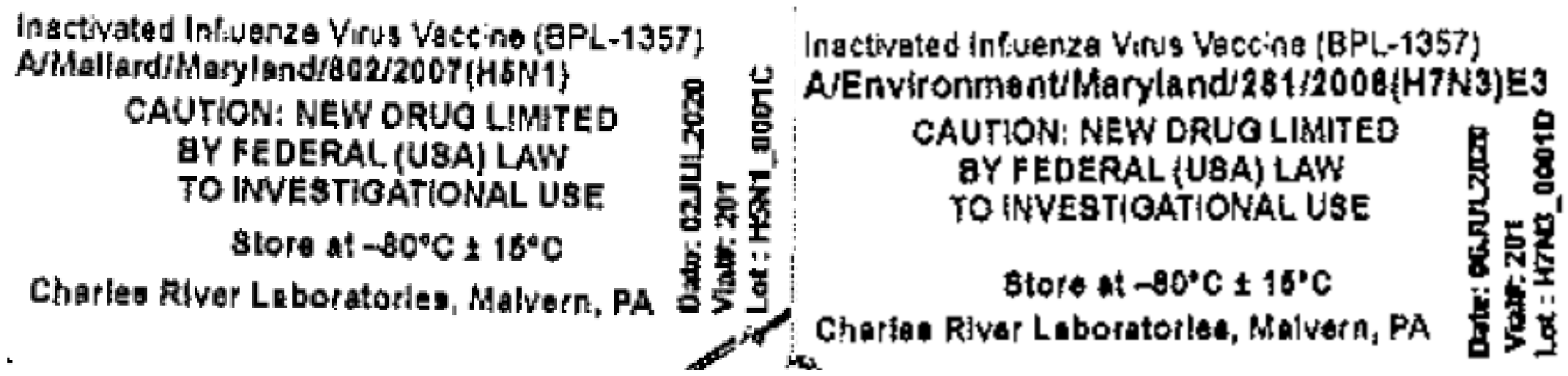

Figure 7: Drug Product Labels

Both BPL-1357 and placebo are colorless liquids and will have identical packaging to maintain blinding.

6.2.3 Product Storage and Stability

BPL-1357 will be handled in accordance with good industrial hygiene and safety practice by the NIH CC Pharmacy. It will be stored at -60°C to -90°C. Partially used vials of BPL-1357 will be stored and used within 8 hours. Stability will be tested every 6 months by measuring the HA activity.

Placebo will be stored according to the saline manufacturer's recommendations.

6.2.4 Preparation

BPL-1357 preparation: BPL-1357 vaccine will be prepared using 4 different inactivated influenza A virus strains. Viruses are vialed individually with a fill volume of 0.3mL and varying concentrations:

1. A/Mallard/Ohio/265/1987 H1N9: 373.62 µg/mL (Lot# H1N9_0002A)
2. A/Pintail/Ohio/339/1987 H3N8: 444.81 µg/mL (Lot# H3N8_0002B)
3. A/Mallard/Maryland/802/2007 H5N1: 385.49 µg/mL (Lot# H5N1_0002C)
4. A/Environment/Maryland/261/2006 H7N3: 364.73 µg/mL (Lot# H7N3_0002D)

Equal 0.25 mL volumes of each virus will be mixed together by the NIH CC Pharmacy prior to administration for a final volume of 1 ml.
For IN vaccination, the vaccine will be drawn up into two syringes at volumes of 0.5 mL for administration in each nostril.
For IM vaccination, the vaccine will be drawn up into a single syringe at a volume of 1 mL.

Table 4: Vaccine Component Total Protein Concentrations, Volumes, and Amounts

Vaccine Component	Total Protein Concentration (ug/mL)	Volume of Each Component (mL)	Total Protein Amount (ug)
**H1N9**	373.62	0.25	93.4

**H3N8**	444.81	0.25	111.2
**H5N1**	385.49	0.25	96.4
**H7N3**	364.73	0.25	91.2

Placebo preparation: 1 mL of normal saline will be drawn up for IM administration or 0.5 mL of normal saline drawn up in each of two syringes for IN administration.

6.3 Measures to Minimize Bias: Randomization and Blinding

6.3.1 Randomization

Participants will be randomized at enrollment in a 1:1:1 ratio to treatment groups A, B, and C. Randomization will occur using computer-generated randomization codes. This code will be sent to the pharmacy where the unblinded pharmacist will have the key and prepare the appropriate agent for administration. The randomization code list will be maintained by the Investigational Drug Management Research Section (IDMRS) in the electronic Investigational Drug Management System (IDMS).

6.3.2 Blinding

The participants, the clinical staff, and the study team will be blinded to study treatment allocation. The IDMRS staff is responsible for maintaining security of the study treatment assignments.

6.3.3 Scheduled Unblinding

After all participants have completed V_2D182, the study will be unblinded. Participants will be informed about their study treatment assignment.

6.3.4 Unscheduled Unblinding

Intentional: Participants who withdraw from the study prematurely will not be unblinded. In cases of emergency, participants will be unblinded in order to provide medical care. To break a treatment blind, the PI will request a participant's treatment assignment from the IDMRS. If an emergency request for treatment assignment is made by an individual other than the PI and the PI is not immediately available, the request will be made to the PI's designee, who will then contact the IDMRS or appropriate NIH Pharmacy after-hours contact to obtain the treatment assignment. If a participant's study agent assignment is unblinded, the information will be provided to only the individuals needing it for treatment decisions; all attempts will be made to maintain the blind of the study team.

Unintentional: If unintentional unblinding of study agent assignment occurs, the PI will create a plan for ongoing management of the participant(s) involved and for preventing the recurrence of a similar incident, as appropriate. If the study team determines that the unintentional unblinding may have a significant impact on the study plan (e.g., if the treatment codes for multiple participants or an entire cohort were accidentally broken), the need for a protocol amendment will be addressed as soon as possible.

Intentional and unintentional unscheduled unblinding will be documented in the appropriate source and/or research record and will include the reason for the unscheduled unblinding, the date it occurred, who approved the unblinding, who was unblinded, who was notified of the unblinding, and the plan for the subject. The PI will report all cases of intentional and unintentional unscheduled unblinding to the data and safety monitoring board (DSMB) in writing within 1 business day after site awareness via email to the DSMB mailbox (niaiddsmbia@niaid.nih.gov) outlining the reason for the unblinding and the date it occurred. The report will also be submitted to the SMM and to the institutional review board (IRB), as appropriate.

If an SAE has resulted in unblinding, this information will be included in the SAE Report (see section 8.4.3.3.2).

6.4 Study Intervention Compliance

Study intervention administration will be documented by study staff in the Clinical Research Information Management System of the NIAID (CRIMSON).

6.5 Concomitant Therapy

All concomitant prescription medications, over-the-counter medications, or herbal remedies taken during study participation must be approved by the PI and will be recorded in the participant's source documents during follow-up visits or on the survey. For this protocol, a prescription medication is defined as a medication that can be prescribed only by a properly authorized/licensed clinician.

Treatment with the following drugs and procedures will not be permitted unless discussed with and approved by the PI:

- Influenza vaccination during the study period, including 8 weeks prior to receiving study agent through the final study visit.
- IN medications (including but not limited to nasal sprays, sinus rinses), over-the-counter medications (including but not limited to aspirin, decongestants, antihistamines, and other nonsteroidal anti-inflammatory drugs), and herbal medications (including but not limited to herbal tea or St. John's Wort) within 14 days of receiving the study agent, during the study period through the final study visit.
- Donation or receipt of blood or blood products within 3 months prior to receiving study agent through the final study visit.
- Administration of unlicensed drug within 3 months or 5.5 half-lives (whichever is greater) prior to receiving the study agent through the final study visit.
- Administration of unlicensed vaccine (not including COVID-19 vaccines under emergency use authorization) within 6 months prior to receiving the study agent through the final study visit.

7 STUDY INTERVENTION DISCONTINUATION AND PARTICIPANT DISCONTINUATION/WITHDRAWAL

7.1 Discontinuation of Study Intervention

Study intervention may be discontinued for a single route (i.e., pausing), or it may be discontinued for all participants and enrollment suspended (i.e., halting). Pausing and halting rules and procedures are described in sections **Error! Reference source not found.** and 8.4.6.

7.2 Participant Discontinuation/Withdrawal from the Study

Participants are free to withdraw from participation in the study at any time upon request.

Criteria and procedures for withdrawal and replacement of a participant by the investigator are provided in section 8.4.4.2.

7.3 Lost to Follow-up

A participant will be considered lost to follow-up if he or she fails to return for one scheduled visit and is unable to be contacted by the study staff.

The following actions must be taken if a participant fails to return to the clinic for a required study visit:

- The site will attempt to contact the participant and reschedule the missed visit within the allotted timeframe for that particular visit, and counsel the participant on the importance of maintaining the assigned visit schedule and ascertain if the participant wishes to and/or should continue in the study.
- Before a participant is deemed lost to follow-up, the investigator or designee will make every effort to regain contact with the participant (where possible, 3 telephone calls, emails, and/or text messages and, if necessary, a certified letter to the participant's last known mailing address or equivalent methods). These contact attempts will be documented in the participant's medical record or study file.
- Should the participant continue to be unreachable, he or she will be considered to have withdrawn from the study with a primary reason of lost to follow-up.

8 STUDY ASSESSMENTS AND PROCEDURES

8.1 Screening Procedures

Participants will be screened and evaluated for eligibility under NIH protocol 11-I-0183. The screening visit will take place within 60 days prior to enrollment (section 5.6).

Screening procedures/assessments performed under protocol 11-I-0183 include the following:

- Review of medical/medication history.
- Vital signs, including blood pressure, mean arterial pressure, heart rate, respiratory rate, temperature, height, weight, assessment of BMI, and measurement of oxygen saturation.

- Clinician assessment/physical exam.
- Blood chemistry testing including acute care, mineral, and hepatic panels, plus total protein, lactate dehydrogenase (LDH), uric acid, and creatine kinase.
- Complete blood count (CBC) with differential.
- Lipid panel.
- HIV and hepatitis B and C testing.
- Serum pregnancy test for females of childbearing potential.
- Urine drug abuse screen (amphetamines, cocaine, benzodiazepines, opiates, and tetrahydrocannabinol).
- EKG.
- Blood collection for storage of serum and RNA isolation for baseline gene expression analysis.

8.2 Efficacy Assessments

8.2.1 Clinical Evaluations

Clinical evaluations will be performed to assess safety (see section 8.3) but not efficacy.

8.2.2 Biospecimen Evaluations

Standard laboratory testing will be performed for safety purposes (see section 8.3) but not for efficacy.

Table 5. Volumes for Specimen Collection

**Laboratory Evaluation**	**Blood Volume**	**Study Visit**								
		**V_1D0**	**V_1D7**	**V_1D14**	**V_2D0**	**V_2D7**	**V_2D14**	**V_2D28**	**V_2D56**	**V_2D182**
CBC + diff	3.0 mL	3.0	3.0	3.0	3.0	3.0	3.0	3.0	3.0	3.0
Chemistry[a]	4.0 mL	4.0	4.0	4.0	4.0	4.0	4.0	4.0	4.0	4.0
HLA testing	10.0 mL	10.0								
Serum for research	5.0 mL × 5	25.0	25.0	25.0	25.0	25.0	25.0	25.0	25.0	25.0
Whole blood collection (PBMC)	10.0 mL × 3	30.0	30.0	30.0	30.0	30.0	30.0	30.0	30.0	30.0
Whole blood collection (PAXgene)	2.5 mL	2.5	2.5	-	2.5	2.5	-	-	-	-
**Daily volume (mL)**		74.5	64.5	62	64.5	64.5	62	62	62	62
**Cumulative volume (mL)**		74.5	139	201	265.5	330	392	454	516	578

CBC + diff = complete blood count with differential; D = day; HLA = human leukocyte antigen; PBMC = peripheral blood mononuclear cells.

[a]Chemistry: Acute care, mineral, and hepatic panels, lactate dehydrogenase, uric acid, creatine kinase, and total protein.

Of note, volume estimations reflect maximums and depending on availability of specimen collection tubes, actual blood volumes collected may be less.

Any clinically relevant test results will be shared with the participant throughout the study. Results of research procedures or evaluations (including incidental findings) will be shared with participants if they are medically actionable. Such results will be discussed with the participant along with guidance for appropriate follow-up with their healthcare provider. Any findings discovered beyond the completion of the primary research will not be returned.

If unexpected or incidental medical conditions are diagnosed during the medical evaluation in this protocol, the participant will be referred to an appropriate physician and/or hospital and encouraged to follow up for treatment of their condition. Standard of care treatment may be offered by the study team if necessary while the participant is being referred to appropriate outside medical care.

8.2.3 Correlative Studies for Research/Pharmacokinetic Studies

Serum (25 mL), whole blood (up to 32.5 mL), and nasal mucosal samples collected at study visits (see section 1.3 for schedule) will be used for research laboratory evaluations described here and in section 8.2.4.

Blood collection will occur using standard methods.

To collect nasal secretions with SAM, the procedure is as follows:

- Wash hands and put on gloves.
- Inspect the nasal cavity using a head lamp and use the nondominant thumb to retract the patient's nose to visualize the nasal cavity.
- Visualize the nasal cavity and inferior turbinate prior to sampling.
- Pass the SAM gently up the lumen of the nostril, orienting it to be flat against the inferior turbinate.
- Ask the participant to use an index finger to press the SAM onto the nasal mucosa.
- After absorption for a minimum of 1 minute, remove the SAM from the nostril and put back into the original tube.
- Repeat in second nostril with another SAM.
- Store tubes in the refrigerator.
- Tubes will be picked up by the study team and undergo further processing and storage by research lab personnel.

To collect nasal epithelial cells with cytology brush, the procedure is as follows:

- The participant will sit comfortably upright with his or her head tilted slightly back.
- Up to 1.5 mL of local anesthetic (4% lidocaine with 1% phenylephrine) will be administered in 1 nostril by nasal atomizer.
- After at least 2 minutes the study staff member will insert a cytology brush through the nasal vestibule along the floor of the nasal cavity, until the brush is alongside the inferior turbinate.
- The brush will be rolled laterally, under the inferior turbinate, then rotated 180 degrees clockwise and counter-clockwise, then rolled away from the inferior turbinate and removed from the nose.
- The brush will then be placed in an appropriate tube and gently agitated for 5 seconds. Then, the brush handle will be cut at the tube opening.
- Store tube in refrigerator.
- The tube will be picked up by the study team and undergo further processing and storage by research lab personnel.

Stored blood samples and mucosal samples will be used for research laboratory evaluations. Endpoints will be evaluated using, but not limited to, the following:

- Antibody titers against H1, H3, H5 and/or H7 head and/or stalk as measured by HAI and/or ELISA from blood and mucosal samples.
- Antibodies against N1, N3, N8, and/or N9 as measured by NAI and/or ELISA from blood and mucosal samples.
- VDJ gene repertoire analysis.
- Cytokine analysis.
- Flow cytometric phenotyping of lymphocytes.
- Transcriptomic gene expression.
- T-cell responses.

Patients with ILI will perform at home nasal sampling with a nasal swab and/or SAM and ship specimens to NIH for testing or will be tested in clinic for influenza (and possibly, other respiratory pathogens).

Laboratory studies are generally performed in the NIAID LID with the exception of transcriptomic analysis which is sent to Kathie Walters at the Institute for Systems Biology.

8.2.4 Samples for Genetic/Genomic Analysis

8.2.4.1 Description of the scope of genetic/genomic analysis

Transcriptomic analysis of gene expression will be performed, including broad RNA sequencing, with a focus on genes which have a change in transcriptomic activity as a response to vaccination. Transcriptomics will be performed on blood and mucosal samples.

8.2.4.2 Description of how privacy and confidentiality of medical information/biological specimens will be maximized

Privacy and confidentiality will be protected as described in section 10.3.

8.2.4.3 Management of results

We do not anticipate that the transcriptomic analyses will result in information that is clinically relevant to participants, so no transcriptomics results will be returned.

8.2.4.4 Genetic counseling

Not applicable.

8.3 Safety and Other Assessments

Evaluations to assess safety will include a review of systems, AE surveys, and physical exam targeting examination of the vaccination site and any concerns raised by the review of systems or AE surveys.

Physical examination: Physical examination will be performed at every visit to identify any potential side effects or AEs related to the intervention.

- Review of medical history.
- Review of systems.
- Vital signs, including weight, temperature, heart rate, blood pressure, mean arterial pressure, respiratory rate, and measurement of oxygen saturation.
- Physical examination including but not limited to general, eye, nasal, oral, lymph, pulmonary, cardiovascular, abdominal, and peripheral extremity examination.

Photographs may be taken if physical examination findings identify a possible side effect.

AE survey: On a daily basis for a 7-day period after vaccination (V_1D0 and V_2D0), participants will evaluate and record temperature and any local or general symptoms they experience via paper or electronic (Research Electronic Data Capture system [REDCap]) survey (APPENDIX A). A thermometer will be provided to facilitate temperature checks and participants will be instructed on how to use it. The survey will be completed by the participant in the evening before going to sleep. Participants will be instructed on how to complete the survey and, for participants using the paper survey, to bring it to the V_1D3, V_1D7, V_2D3, and V_2D7 study visits. The survey will be reviewed by the study team and evaluated for AEs. If surveys are not completed, the

study team will review the AEs that occurred during that timeframe to the best of the participant's recollection.

Biological specimen collection and laboratory evaluations: Approximately 7 mL of blood will be obtained at each visit to test for AEs. The following tests will be obtained:

- Blood chemistry testing including acute care, mineral, and hepatic panels, plus total protein, LDH, uric acid, and creatine kinase.
- CBC with differential.

Urine will be collected for a drug abuse screen (amphetamines, cocaine, benzodiazepines, opiates, and tetrahydrocannabinol) and pregnancy testing according to the schedule in section 1.3.

Unscheduled interim visit: If medically necessary (e.g., if a safety condition arises), the participant may be asked to return for an unscheduled visit for AE assessment. This visit may include any of the safety assessments listed above or other testing as deemed necessary by the PI or designee.

8.4 Safety Definitions, Management, and Sponsor Reporting

8.4.1 Definitions

The NIAID Clinical Safety Office (CSO) is responsible for sponsor safety oversight of this study, and the definitions below comply with CSO requirements.

Adverse Event: An AE is any untoward or unfavorable medical occurrence in a human subject, including any abnormal sign (e.g., abnormal physical exam or laboratory finding), symptom, or disease, temporally associated with the subject's participation in the research, whether or not considered related to the research.

Adverse Reaction (AR): An AR means any AE caused (see "Causality" below) by a study agent. ARs are a subset of all suspected adverse reactions (SARs; defined below) where there is reason to conclude that the study agent caused the event.

Suspected Adverse Reaction (SAR): SAR means any AE for which there is a reasonable possibility that the study agent caused the AE.

> Per US FDA guidance:
> For the purposes of IND safety reporting, "reasonable possibility" means there is evidence to suggest a causal (see "Causality" below) relationship between the study agent and the AE. A SAR implies a lesser degree of certainty about causality than an AR, which means any AE caused by a study agent.
>
> SARs are the subset of all AEs for which there is a reasonable possibility that the study agent caused (see "Causality" below) the event. Inherent in this definition, and in the requirement

to report SARs, is the need for the sponsor to evaluate the available evidence and make a judgment about the likelihood that the study agent actually caused the AE.

The sponsor is responsible for making the causality judgment.

Serious Adverse Event: An SAE:

- is an AE that results in death.
- is an AE that is life-threatening event (places the subject at immediate risk of death from the event as it occurred).
- is an AE that requires inpatient hospitalization or prolongs an existing hospitalization.

  NOTE:

  - Hospitalization is considered required if outpatient treatment would generally be considered inappropriate.
  - Same-day surgical procedures that are required to address an AE are considered hospitalizations, even if they do not involve an overnight admission.
  - Hospitalization due to a condition that has not worsened and that pre-dates study participation (e.g., elective correction of an unchanged baseline skin lesion), or due to social circumstance (e.g., prolonged stay to arrange aftercare), or that is planned/required "per protocol" AND that proceeds without prolongation or complication, is NOT considered an SAE by this criterion.
- is, or results in, a persistent or significant incapacity or substantial disruption of the ability to conduct normal life functions.
- is a congenital anomaly/birth defect/miscarriage/stillbirth.

  NOTE: This definition is more inclusive than some commonly published definitions. It includes an affected conceptus/neonate whose:

  - biological mother was exposed to a study agent at any point from conception through the end of the pregnancy, AND/OR, if breastfeeding, the 30-day neonatal period; or
  - biological father was exposed to a study agent at any point during the 90 days prior to conception.

  This is separate from, and in addition to, general reporting of pregnancy in a study participant or female partner of a male participant (see section 8.4.3.3.4 below).
- is a medically important event.

  NOTE: Medical and scientific judgment should be exercised. Events that significantly jeopardize the subject and/or require intervention to prevent one of the SAE outcomes listed above are generally considered medically important, and are thus SAEs.

Unexpected Adverse Event: An AE is unexpected if it is not listed in the investigator's brochure or package insert (for marketed products) at the frequency, AND specificity, AND severity that has been observed.

NOTE:

- Such events should also be evaluated for possible reporting as unanticipated problems (UPs) (see section 8.4.3.3.3 below).
- Unexpected, as used in this definition, also refers to AEs or SARs that are mentioned in the investigator's brochure as occurring with a class of drugs/biologics, or as anticipated from the pharmacological properties of the study agent but are not specifically mentioned as occurring with the particular study agent under investigation.

Serious and Unexpected Suspected Adverse Reaction (SUSAR): A SUSAR is an SAR (defined above) that is both serious and unexpected.

Unanticipated Problem: A UP is any incident, experience, or outcome that meets all the following criteria:

1. Unexpected (in terms of nature, severity, or frequency) given
   a. the research procedures that are described in the protocol-related documents, such as the IRB-approved research protocol, investigator's brochure, and informed consent document; and
   b. the characteristics of the subject population being studied; and
2. Related or possibly related to participation in the research (possibly related means there is a reasonable possibility that the incident, experience, or outcome may have been caused by the procedures involved in the research), and
3. Suggests the research places subjects or others (which may include research staff, family members or other individuals not directly participating in the research) at a greater risk of harm (including physical, psychological, economic, or social harm) related to the research than was previously known or expected.

   NOTE:

   - Per the sponsor, an SAE always meets this "greater risk" criterion.
   - An incident, experience, or outcome that meets the definition of a UP generally will warrant consideration of changes to the protocol or informed consent form, or to study procedures (e.g., the manual of procedures for the study), in order to protect the safety, welfare, or rights of participants or others. Some UPs may warrant a corrective and preventive action plan (CAPA) at the discretion of the sponsor or other oversight entities.

Unanticipated Problem that is not an Adverse Event (UPnonAE): A UPnonAE belongs to a subset of UPs that:

- meets the definition of a UP, AND
- does NOT fit the definition of an AE or an SAE.

  NOTE: Examples of UPnonAEs include, but are not limited to:

  - a breach of confidentiality
  - prolonged shedding of a vaccine virus beyond the anticipated timeline
  - unexpectedly large number of pregnancies on a study
  - subject departure from an isolation unit prior to meeting all discharge criteria
  - accidental destruction of study records
  - unaccounted-for study agent
  - overdosage, underdosage, or other significant error in administration or use of study agent or intervention, even if there is no AE/SAE
  - development of an actual or possible concern for study agent purity, sterility, potency, dosage, etc

    NOTE: A decision to temporarily quarantine, or to permanently not use all or part of study agent supply due to an unexpected finding or event (e.g., particulate, cloudiness, temperature excursion), even if there is no known or proven issue (i.e., out of an "abundance of caution"), is considered a UPnonAE.

Protocol Deviation: Any change, divergence, or departure from the IRB-approved research protocol.

1. **Major Deviations:** Deviations from the IRB-approved protocol that have, or may have the potential to, negatively impact the rights, welfare, or safety of the subject, or to substantially negatively impact the scientific integrity or validity of the study.
2. **Minor Deviations:** Deviations that do not have the potential to negatively impact the rights, safety, or welfare of subjects or others, or the scientific integrity or validity of the study.

Non-compliance: Failure of investigator(s) to follow the applicable laws, regulations, or institutional policies governing the protection of human subjects in research, or the requirements or determinations of the IRB, whether intentional or not.

1. **Serious non-compliance:** Non-compliance, whether intentional or not, that results in harm or otherwise materially compromises the rights, welfare and/or safety of the subject. Non-compliance that materially affects the scientific integrity or validity of the research

may be considered serious non-compliance, even if it does not result in direct harm to research subjects.

2. **Continuing non-compliance:** A pattern of recurring non-compliance that either has resulted, or, if continued, may result in harm to subjects or otherwise materially compromise the rights, welfare and/or safety of subjects, affect the scientific integrity of the study or validity of the results. The pattern may comprise repetition of the same non-compliant action(s), or different non-compliant events. Such non-compliance may be unintentional (e.g. due to lack of understanding, knowledge, or commitment), or intentional (e.g. due to deliberate choice to ignore or compromise the requirements of any applicable regulation, organizational policy, or determination of the IRB).

8.4.2 Protocol-Specified Exempt Events (PSEEs)

PSEEs are events, identified in detail within the protocol and specified in advance, that will be handled in a protocol/study-specific manner that **differs from statutory and general rules for reporting**. These events may include, but are not limited to:

- laboratory values
- clinical findings
- statutory AEs or SAEs per the standard definition
- hospitalizations and procedures

PSEEs are protocol/study-specific rules for events that are, to some degree, expected/anticipated (due to population or condition under study, concomitant treatments, etc.). Reporting is generally **downgraded/diminished from the general standard**.

8.4.3 Documenting, Assessing, Recording, and Reporting Events

ALL AEs, including those that may appear to have a non-study cause (see "Causality" below), will be documented (e.g., on the clinical chart/progress notes/clinical laboratory record), recorded (e.g., in the study-specified research database, CRIMSON), and reported (e.g., cumulatively from the research database, or according to protocol-specified expedited reporting mechanism) to the sponsor from the time informed consent is obtained through the timeframe specified below. At each contact with the subject, information regarding AEs will be elicited by open-ended questioning and examinations.

AEs and SAEs will generally be recorded, assessed, and reported according to the timeframes outlined in Table 6. Standard Event Recording, Assessment, and Reporting Timeframes. Descriptions of PSEEs and timeframes for assessing and reporting these events are outlined in Table 7. PSEE List and Reporting.

Table 6. Standard Event Recording, Assessment, and Reporting Timeframes

Event type	Record, assess, and report through

Related SAEs	End of subject participation in study, or if study personnel become aware thereafter
Unrelated SAEs	End of subject participation in study
Related non-serious AEs of grade 1 to 3	End of subject participation in study
All other related non-serious AEs	End of subject participation in study
Unrelated non-serious AEs	End of subject participation in study

Table 7. PSEE List and Reporting

Event description	How event will be reported
• Grade 1 lab and vital sign abnormalities found prior to study agent administration and deemed not clinically significant by the PI.	• If baseline abnormalities increase in severity or resolve and then recur after study agent administration, report as AEs according to standard reporting procedures.

8.4.3.1 Investigator Assessment of Adverse Events

The investigator will assess all AEs with respect to **seriousness** (according to SAE definition above), **severity** (intensity or grade, see below), and **causality** (relationship to study agent and relationship to participation in the research, see below).

8.4.3.1.1 Severity Grading

The investigator will grade the severity of each AE, including laboratory and testing abnormalities and results, according to the "Guidance for Industry: Toxicity Grading Scale for Healthy Adult and Adolescent Volunteers Enrolled in Preventive Vaccine Clinical Trials" which can be found at: https://www.fda.gov/regulatory-information/search-fda-guidance-documents/toxicity-grading-scale-healthy-adult-and-adolescent-volunteers-enrolled-preventive-vaccine-clinical

Events that are NOT gradable using the above specified table will be graded as follows:

- Mild = grade 1
- Moderate = grade 2
- Severe = grade 3

- Potentially life threatening = grade 4
- Death = grade 5

NOTE: A subject death should always be reported as grade 5.

8.4.3.1.1.1 Laboratory Value Assessment and Clinical Significance Criteria

Except as specified in Table 7 and below, ALL abnormal lab values of grade 1 or above are REPORTABLE.

Grade 1 and 2 abnormal laboratory values are considered CLINICALLY SIGNIFICANT, and are to be recorded in the research database, and reported, if they meet ONE or more of the following criteria:

- result in a study agent dosage adjustment, interruption, or discontinuation
- are accompanied by clinically abnormal signs or symptoms that are likely related to the laboratory abnormality (e.g., clinical jaundice)
- indicate a possible organ toxicity (e.g., elevated serum creatinine)
- result in additional/repeat testing or medical intervention (procedures/treatments) (e.g., EKG to evaluate arrhythmia potential with a high serum potassium; one or more EKGs to assess an elevated troponin level; potassium supplementation for hypokalemia)
- indicates possible over-dosage
- are considered clinically significant by the investigator or SMM

8.4.3.1.1.2 Laboratory Toxicity Table Adjustments

Not applicable

8.4.3.1.2 Causality

Causality (likelihood that the event is caused by the study agents) will be assessed by the PI considering the factors listed under the following categories:

Definitely Related

- reasonable temporal relationship
- follows a known response pattern
- clear evidence to suggest a causal relationship
- there is no alternative etiology

Probably Related

- reasonable temporal relationship
- follows a suspected response pattern (based on similar agents)
- no evidence of a more likely alternative etiology

Possibly Related

- reasonable temporal relationship
- little evidence for a more likely alternative etiology

Unlikely Related

- does not have a reasonable temporal relationship

AND/OR

- there is good evidence for a more likely alternative etiology

Not Related

- does not have a temporal relationship

AND/OR

- definitely due to an alternative etiology

Note: Other factors (e.g., dechallenge, rechallenge, if applicable) should also be considered for each causality category when appropriate. Causality assessment is based on available information at the time of the assessment of the AE. The investigator may revise the causality assessment as additional information becomes available.

Causality assessment will be reviewed by the sponsor. The sponsor may make a separate and final determination on the "reasonable possibility" that the event was "related" (comprising definitely, probably, and possibly related) or "unrelated" (comprising unlikely and not related) to the study agent, in keeping with applicable (US FDA) guidance on sponsor IND safety reporting.

8.4.3.2 Recording of Events

AEs will be promptly recorded in the research database, regardless of possible relationship to study interventions. If a diagnosis is clinically evident (or subsequently determined), the diagnosis rather than the individual signs and symptoms or laboratory abnormalities will be recorded as the AE. The investigator will review events regularly to ensure they have been captured correctly and to perform assessment of events individually and cumulatively to assess possible safety trends.

8.4.3.3 Investigator Reporting Responsibilities

The PI and/or equally qualified designee will check daily for events that may require expedited reporting.

The PI and/or equally qualified designee will also monitor all accumulating data no less than weekly, or according to superseding NIH or NIAID policy, whichever is more frequent.

Data will be reviewed by the PI/designee on a regular basis for accuracy and completeness.

Data will be submitted to the sponsor in keeping with all applicable agreements and when requested, such as for periodic safety assessments, review of IND annual reports, review of IND safety reports, and preparation of final study reports.

The PI and/or other study designee will ensure prompt reporting to safety oversight bodies (e.g., CSO, DSMB), regulatory entities, and stakeholders as specified below, and according to any additional requirements or agreements.

8.4.3.3.1 Adverse Events

Unless otherwise specified above, AE data will be entered into the research database no less than every other week and will include all data through one week prior to database entry. AEs, including laboratory abnormalities, will be followed until resolution or stabilization.

8.4.3.3.2 Serious Adverse Events (Expedited Reporting)

Unless otherwise specified above, all SAEs (regardless of relationship and whether or not they are also UPs) must be reported to the CSO as specified by the CSO (e.g., REDCap system; use the Safety Expedited Report Form [SERF]/email if REDCap is not available). If the preferred/indicated mechanism for reporting is not available, the CSO/SMM should be contacted by telephone, fax, or other reasonable mechanism to avoid delays in reporting.

CSO CONTACT INFORMATION:
Clinical Safety Office
5705 Industry Lane
Frederick, MD 21704
Phone: 301-846-5301
Fax: 301-846-6224
Email: rchspsafety@mail.nih.gov
REDCap access: https://crimsonredcap.cc.nih.gov/redcap/index.php

Unless otherwise specified above, deaths and immediately life-threatening SAEs must be reported to the CSO promptly, and no later than the **first business day** following the day of study personnel awareness.

All other SAEs must be reported to the CSO no later than the **third business day** following the day of study personnel awareness.

If an individual subject experiences multiple SAEs in a closely timed/overlapping "cause-and-effect" (cascade) sequence, the PI, after careful evaluation, will report ONLY primary/precipitating event(s) individually. SAEs that are determined to be definitely secondary to other SAEs will be detailed in the narrative portion of the report of the relevant primary/precipitating SAE. A clinical rationale and findings to support such reporting should be part of the narrative.

For each SAE report, the research database entry MUST match the corresponding entries on the SAE report (e.g., start and stop dates, event type, relationship, and grade), and **must be updated if necessary** (e.g., if the SAE report was generated after the corresponding AE was entered in the research database).

Unless otherwise specified above, SAEs that have not resolved by the end of the per-protocol follow-up period for the subject are to be followed until final outcome is known (to the degree permitted by the IRB-approved informed consent form). If it is not possible to obtain a final outcome for an SAE (e.g., the subject is lost to follow-up), and to update the CSO, the last known status and the reason a final outcome could not be obtained will be recorded by the investigator on an SAE report update and in CRIMSON.

8.4.3.3.3 Unanticipated Problems

Unless otherwise specified above, UPs (as defined in this protocol, or as defined by the IRB of record, whichever definition is more conservative) that are also AEs or SAEs, must be reported to the CSO (by REDCap, or by email and SERF if REDCap is not available) no later than when they are due to be reported to the IRB.

UPnonAEs are NOT reported to the CSO but must be reported to the Clinical Trials Management (CTM) group and the IRB according to their requirements and preferred methods. If the UPnonAE raises a significant potential subject safety concern, the SMM should be consulted by email or phone no later than when reports are made to the IRB and/or CTM and provide details of the UPnonAE.

8.4.3.3.4 Pregnancy

Unless otherwise specified above, all pregnancies will be reported (by REDCap, or by email and SERF if REDCap is not available) to the CSO no later than the first business day following the day of study personnel awareness.

Pregnancy outcome data (e.g., delivery outcome, spontaneous or elective termination of the pregnancy) will be reported to the CSO no later than the third business day following the day of study personnel awareness (by REDCap, or by email and SERF if REDCap is not available).

Pregnancy itself is not an AE. Events that meet AE or SAE criteria in relation to pregnancy, delivery, or the conceptus/neonate (see section 8.4.1) are reportable (by REDCap, or by email and SERF if REDCap is not available).

In the event of pregnancy in a study subject exposed to study agent, the following actions will be taken, with the goal of ensuring maternal and fetal well-being, in consultation with the SMM and DSMB:

- Discontinue the following study-specific procedures:
  - Collection of whole blood (peripheral blood mononuclear cells [PBMC] and PAXgene for RNA) and serum for research testing.
  - Nasal SAM.

    - Nasal brushing.
- Continue to follow for safety for the duration of the pregnancy.
- Request to unblind the subject, if applicable, AND if doing so would offer a benefit to the subject.
- Report, no later than the first business day after study personnel awareness, to the DSMB and/or the IRB.
- Advise subject to notify the obstetrician of study participation and study agent exposure, providing contact information for the obstetrician to contact the study PI, should this be required, and with the subject's consent.

8.4.3.4 Sponsor's Reporting Responsibilities

Events reported to the sponsor will be promptly evaluated and will be reported as required according to FDA IND safety reporting guidance and regulations. IND safety reports will be sent to other investigators conducting research under the same IND and will be shared with other stakeholders according to applicable agreements.

The sponsor will also submit an IND annual report of the progress of the investigation to the FDA as defined in 21 CFR 312.33.

All UPs will be evaluated by the sponsor, and a summary of the event, and any necessary (corrective/preventative) actions, will be distributed to investigators conducting research under the same IND as may be relevant and appropriate.

8.4.4 Withdrawal Criteria for an Individual Subject

An individual subject will be withdrawn from the study for any of the following:

- An individual subject's decision. (The investigator should attempt to determine the reason for the subject's decision.)
- Non-compliance with study procedures to the extent that it is potentially harmful to the subject or to the integrity of the study data.
- A change in the subject's condition as follows:
    - At the PI's discretion, development of any condition so that the participant no longer meets one or more of the eligibility criteria.
- The investigator determines that continued participation in the study would not be in the best interest of the subject.

Participants may withdraw prior to vaccine administration and no further testing or follow-up will be performed. The PI or designee will discuss with the participant why she/he wants to prematurely withdraw from the study to determine the best course of action for the participant. If the participant would like to withdraw after vaccination, clinical laboratory tests and procedures for safety purposes will continue as obtainable and at a frequency determined by the PI. If the participant does not return for scheduled follow-up visits, the study staff will make every

reasonable effort to contact the participant by phone, mail, or email, or a combination of the three and reiterate that follow-up visits are strongly encouraged for safety reasons.

8.4.4.1 Re-enrollment and Unplanned Procedure Repetition

Unless otherwise specified within this protocol, each person who is a subject in this study may be enrolled, and may pass through each step and process outlined in the protocol, only **ONCE** (i.e., subjects may not "go back" and repeat a protocol step already completed). On a case-by-case basis, a request for re-enrollment, or for repetition of a protocol step or procedure already completed, may be submitted to, reviewed by, and approved by the SMM in writing. The SMM may also recommend or require consultation of the IRB and/or DSMB.

8.4.4.2 Replacement of Withdrawn Subjects or Subjects Who Discontinue Study Agent

A participant who withdraws before study agent administration will be removed, and no data will be used in the safety or efficacy analysis or publication of the study. If this occurs, another participant from the over accrual may be added and randomized to a trial arm.

Additional participants from the over accrual may also be added to compensate for participants who withdraw or are lost to follow-up after study vaccine administration on V_1D0 but before the V_2D7 visit.

All subjects exposed to study agents will be included in the safety dataset.

8.4.5 Additional Safety Oversight

8.4.5.1 Safety Review and Communications Plan

A safety review and communication plan (SRCP) is required for this protocol. The SRCP is an internal communications document between the PI and the CSO, as sponsor representative, which delineates key safety oversight responsibilities of the PI, the CSO, and other stakeholders. The SRCP includes a plan for conducting periodic safety surveillance assessments by the CSO.

8.4.5.2 Sponsor Medical Monitor

A SMM, representing the sponsor, has been appointed for oversight of safety in this clinical study. The SMM will be responsible for performing safety assessments as outlined in the SRCP.

8.4.5.3 Oversight Committees

8.4.5.3.1 Data and Safety Monitoring Board

The NIAID intramural DSMB includes independent experts that do not have direct involvement in the conduct of the study and have no significant conflicts of interest as defined by NIAID policy. The DSMB will review the study protocol, consent document, and investigator brochure prior to initiation and at least once a year thereafter, or at a frequency determined by the DSMB..

The DSMB may convene additional reviews as necessary. The DSMB will review the study data as needed to evaluate the safety, efficacy, study progress, and conduct of the study.

All deaths, SAEs, UPs, pregnancies, and IND safety reports will be reported to the DSMB at the same time they are submitted to the IRB and CSO unless otherwise specified herein. A summary of PSEEs will also be provided for review by the DSMB along with study documents submitted for periodic scheduled DSMB reviews.

All cases of intentional or unintentional unblinding will be reported to the DSMB not later than one business day from the time of study personnel awareness.

The PI will notify the DSMB at the time pausing or halting criteria are met and obtain a recommendation concerning continuation, modification, or termination of the study. The PI will submit the written DSMB summary reports with recommendations to the IRB.

8.4.6 Pausing Rules for the Protocol

"Pausing" is discontinuation of study intervention/treatment/dosing (agent/placebo/procedure, etc) in a protocol-defined dose group or "arm" at the discretion of the sponsor until a decision is made to either resume or permanently discontinue such activity. The IM or IN route of study agent administration may be paused at the discretion of the DSMB who will not be blinded. The investigators will continue to be blinded as to which study arm is paused. Subjects will continue to be followed for safety during a pause.

The pausing criteria for a group of subjects in this study include any one or more of the following:

- A subject experiences an SAE that is unexpected (per the investigator's brochure or product label) and possibly, probably, or definitely related to a study agent or route of administration;
- A subject experiences two grade 3 or greater AEs that are unexpected (per the investigator's brochure or product label) and possibly, probably, or definitely related to a study agent or route of administration.

The PI or the CSO may also pause dosing/study interventions for one or more subjects for any safety issue. The DSMB may recommend a pause to the CSO.

8.4.6.1 Reporting a Pause

If a pausing criterion is met, a description of the AE(s) or safety issue must be reported by the PI within 1 business day to the CSO and the IRB according to their requirements. The PI will also notify the DSMB and FDA. In the event of a SUSAR, the FDA will also be notified by sponsor.

8.4.6.2 Resumption Following a Pause

The CSO, in collaboration with the PI and DSMB, will determine if study activities, including agent administration and/or other study interventions, may be resumed, and any additional modifications or requirements that may apply, for the impacted subject(s), or whether the events that triggered the pause require expansion to a study halt (see section 8.4.6).

The CSO or sponsor designee will notify the PI of the decision. The PI will notify the IRB of the decision according to the IRB's process.

8.4.6.3 Discontinuation of Study Agent

Subjects who received study agent/study intervention will continue to be followed for protocol-specified safety assessments or as clinically indicated, whichever is more conservative, during a study pause.

8.4.7 Halting Rules for the Protocol

"Halting" is discontinuation of study intervention/treatment/dosing (agent/placebo/procedure, etc.) for all subjects in a study and suspension of enrollment until a decision is made to either resume or permanently discontinue such activity. Subjects continue to be followed for safety during a halt.

The halting rules are:

- Two or more SAEs that are unexpected (in consultation with the sponsor) that are possibly, probably, or definitely related to the study agent(s);

  OR

- Two or more subjects experience the same or similar grade 3 or greater AEs that are unexpected and possibly, probably, or definitely related to a study agent;

  OR

- Any safety issue that the PI or the CSO determines should halt the study. The DSMB may recommend a halt to the CSO.

In addition, the FDA or any regulatory body having oversight authority may halt the study at any time.

8.4.7.1 Reporting a Study Halt

If a halting criterion is met, a description of the AE(s) or safety issue must be reported by the PI within 1 business day to the CSO and the IRB according to their requirements. The PI will also notify the DSMB.

8.4.7.2 Resumption of a Halted Study

The CSO, in collaboration with the PI, DSMB, and FDA (if applicable), will determine if study activities, including enrollment, study agent administration, and/or other study interventions, may be resumed and any additional modifications or requirements that may apply.

The CSO or sponsor designee will notify the PI of the decision. The PI will notify the IRB of the decision according to the IRB's process.

8.4.7.3 Discontinuation of Study Agent

Subjects who received study agent/intervention/treatment will continue to be followed for protocol-specified safety assessments or as clinically indicated, whichever is more conservative, during a halt.

8.5 Unanticipated Problems

8.5.1 Definition of Unanticipated Problems

The definition of a UP is provided in section 8.4.1.

8.5.2 Unanticipated Problem Reporting

The investigator will report UPs to the NIH IRB according to NIH Human Research Protections Program (HRPP) Policy 801.

8.5.3 NIH Intramural IRB Reporting of IND Safety Reports

Only IND Safety Reports that meet the definition of a UP will need to be reported to the NIH IRB.

8.6 Additional Reporting Requirements

8.6.1 Reporting to the NIH IRB

Non-compliance and other reportable events will be reported to the NIH IRB according to HRPP Policy 801.

8.6.2 Reporting to the NIAID Clinical Director

The PI will report UPs, major protocol deviations, and deaths to the NIAID clinical director according to institutional timelines.

9 STATISTICAL CONSIDERATIONS

9.1 Statistical Hypothesis

The primary hypothesis of this study is that IN or IM BPL-1357 will be well tolerated with minor side effects.

9.2 Sample Size Determination

There will be 15 participants in each treatment group. The study will have adequate power to observe safety signals as defined by the 2 pausing criteria.

- A subject experiences an SAE that is unexpected (per the investigator's brochure or product label) and possibly, probably, or definitely related to a study agent or route of administration;

- A subject experiences two grade 3 or greater AEs that are unexpected (per the investigator's brochure or product label) and possibly, probably, or definitely related to a study agent or route of administration.

Table 7 gives the probability of meeting either criteria in just one arm under various true rates. The table shows that if the true rate in a single vaccine arm is 15% there would be a high probability of detecting the safety signal (power of 0.913). If we meet pausing criteria in both arms, then we also meet halting criteria for study.

Table 8. Probability of meeting pausing criteria

True rate of pausing criteria 1 or 2 (%)	Probability of pausing (≥ 1/15)
5%	0.537
10%	0.794
15%	0.913

We will also compare AE rates for the combined vaccine arms to the placebo arm at V_2D28. We will use a 1-sided .05 level test for the comparison of proportions. With this test we will have 80% power if the true rate in the placebo arm is 5% and the true rate in the vaccine arms is 36%. We will also have 80% power if the true rate in the placebo arm is 10% and the true rate in the vaccine arms is 44%. (This calculation was performed using the SWOG statistical tool website with no continuity correction).

For the safety secondary objective we will compares AE rates in a similar manner but at the end of study, V_2D182. For the immunogenicity secondary objective, we will compare antibodies against H1, H3, H5, H7, N1, N3, N8, and N9 from both blood and mucosal samples between all 3 arms at V_2D28. With 15 participants in each arm there will be power of 0.82 to detect an effective size of 1.25 (the effective size is the mean/std) using a two-sided 0.05/3=.0167 level t-test. The level of the test has been adjusted to control for the 3 comparisons between all groups. A similar analysis will be performed at other timepoints for one of the exploratory objectives.

9.3 Populations for Analyses

Safety will be evaluated with intention-to-treat analysis comparing IN vs IM routes. All subjects receiving the vaccine will be included in the analysis.

9.3.1 Evaluable for Toxicity

All participants will be evaluable for toxicity from the time of their first treatment with BPL-1357 or placebo.

9.3.2 Evaluable for Objective Response

Not applicable.

9.3.3 Evaluable Non-Target Disease Response

Not applicable.

9.4 Statistical Analyses

9.4.1 General Approach

AE's and SAE's will be tabulated by severity and by group. Tests of proportions with no continuity correction will be used to test for differences between groups. Confidence intervals around the difference in proportions will be calculated. Antibodies will be compared using t-tests. Antibody data may be transformed by using log transformation to stabilize the variance if the data are skewed.

9.4.2 Analysis of the Primary Endpoints

If 1 or more related SAE's are observed in an arm, the primary safety endpoint for that arm will have been met. For the AE safety endpoints, summary statistics will be provided for unexpected safety events. Unexpected safety events will be summarized by treatment group, as well as by individual AE and grouped by type of event. Tests of proportions will be used to compare rates between groups. 90% confidence intervals will be calculated for safety endpoints. This will be done at V_2D28 for the primary endpoint, but a similar analysis will be done for the secondary safety endpoint at V_2D182.

Two-sided t-tests will be used to compare antibody endpoints at V_2D28 for the secondary objective. All three arms will be compared to each other. A 0.05/3= 0.0167 significance level will be used to adjust for the 3 group comparisons. Confidence intervals around group differences will be calculated using the significance level adjusting for the 3 comparisons (each confidence interval will be at the 1-0.0167 = 0.983 level). Antibody data maybe transformed by using the log10 transformation to stabilize the variance if the data are skewed.

9.4.3 Baseline Descriptive Statistics

Baseline population characteristics including gender, race, ethnicity, age, and BMI will be presented. Statistical analysis will not be performed on baseline characteristics.

9.4.4 Sub-Group Analyses

Subgroup differences between various age groups or races are not expected and the sample size may be too small to determine statistically significant differences, but exploratory analyses may be performed (particularly on gender differences).

9.4.5 Tabulation of Individual Participant Data

Not applicable.

9.4.6 Exploratory Analyses

Exploratory endpoints include characterization of systemic/mucosal humoral responses at all timepoints, as well as further characterization of the immune response through flow cytometric

phenotyping of lymphocytes, cytokine analysis, transcriptomics, and assessment of T-cell responses. Given the exploratory nature of these endpoints, what analyses will be possible depends heavily on the results. Standard methods of transcriptomic, T-cell activity, cytokine analysis, and immunophenotyping analysis will be performed, followed by more focused analyses based on those initial results.

Categorical endpoints will be analyzed using Fishers exact test. Logistic regression may be used to adjust for covariates. Continuous endpoints will be analyzed using t-tests. Regression models may be used to adjust for covariates. Generalized estimating equation (GEE) models may be used to examine longitudinal endpoints. All exploratory analyses will be performed at the .05 level with all publications noting the endpoints as exploratory and a discussion on the number of endpoints that have been analyzed will be included.

Rates of influenza disease among groups will be compared using analysis of variance testing and post-hoc Tukey.

10 REGULATORY AND OPERATIONAL CONSIDERATIONS

10.1 INFORMED CONSENT PROCESS

10.1.1 Consent/Assent Procedures and Documentation

Informed consent is a process where information is presented to enable persons to voluntarily decide whether or not to participate as a research participant. It is an ongoing conversation between the human research participant and the researchers, which begins before consent is given and continues until the end of the participant's involvement in the research. Discussions about the research will provide essential information about the study and include purpose, duration, experimental procedures, alternatives, risks, and benefits. Participants will be given the opportunity to ask questions and have them answered.

The participants will sign the informed consent document prior to undergoing any research procedures. The participants may withdraw consent at any time throughout the course of the trial. A copy of the informed consent document will be given to the participants for their records. The researcher will document the signing of the consent form in the participant's medical record. The rights and welfare of the participant will be protected by emphasizing to them that the quality of their medical care will not be adversely affected if they decline to participate in this study.

The study will be introduced and explained to the potential participant during the screening visit on protocol 11-I-0183, which will be within 60 days of the V_1D0 visit. Informed consent will be obtained at the V_1D0 clinic visit prior to enrolling into the study. Consent will be obtained in person by the PI and/or equally qualified designee.

10.1.2 Consent for Minors When They Reach the Age of Majority

Not applicable.

10.1.3 Telephone Consent

Not applicable.

10.1.4 Telephone Assent

Not applicable.

10.1.5 Participation of Subjects Who Are/Become Decisionally Impaired

Adults unable to give consent are excluded from enrolling in the protocol. However, re-consent may be necessary and there is a possibility, though unlikely, that subjects could become decisionally impaired. Because there is no prospect of direct benefit from participation in this study, if a subject becomes decisionally impaired while on study, they may continue to undergo safety evaluations if deemed appropriate by the PI in consultation with the SMM. All remaining research interventions will be discontinued.

10.1.1 Participation of NIH Employees

For NIH employee participants, consent will be obtained by an individual independent of the employee's team whenever possible. Otherwise, the consent procedure will be independently monitored by the CC Department of Bioethics Consultation Service in order to minimize the risk of undue pressure on the staff member. Those in a supervisory position to any employee and co-workers of the employee will not obtain consent.

10.2 Study Discontinuation and Closure

The study may be temporarily suspended or permanently terminated as described in the halting rules (section 8.4.6). In addition to the reporting described in that section, the PI will promptly contact the study participants, provide the reason(s) for the termination or suspension, and, if applicable, inform them of changes to study visit schedule.

The PI will consult with the IRB, in addition to the CSO, DSMB, and FDA prior to resuming the study following a halt.

10.3 Confidentiality and Privacy

All records will be kept confidential to the extent provided by federal, state, and local law. The study monitors and other authorized representatives of the sponsor may inspect all documents and records required to be maintained by the investigator, including but not limited to, medical records. Records will be kept locked and data will be coded. Any personally identifiable information maintained for this study will be kept on restricted-access computers and networks. Personally identifiable information will only be shared with individuals authorized to receive it under this protocol. Individuals not authorized to receive personally identifiable information will be provided with coded information only, as needed. Clinical information will not be released without written permission of the participant, except as necessary for monitoring by IRB, the FDA, the NIAID, the Office for Human Research Protections (OHRP), or the sponsor's designee.

Access to research samples will be limited using a locked freezer in a locked laboratory. Samples and data will be stored using codes assigned by the investigators. Data will be kept in password-protected computers. Only investigators and their designees will have access to the samples and data. Samples will initially be stored in laboratories of the PI and associate investigators. Samples will be tracked using a database located on password-protected computers, which will be maintained by the investigators and their designees. Only investigators and their designees will have access to this database.

Any loss or unanticipated destruction of samples (for example, due to freezer malfunction) or data (for example, misplacing a printout of data with identifiers) that meets the definition of a reportable event will be reported to the IRB according to Policy 801.

Participants may decide at any point during study participation or after not to have their samples stored. In this case, the PI will destroy all known remaining samples and report what was done to both the participant and to the IRB. This decision will not affect the subject's participation in this protocol or any other protocols at NIH.

To further protect the privacy of study participants, a Certificate of Confidentiality has been issued by the NIH. This certificate protects identifiable research information from forced disclosure. It allows the investigator and others who have access to research records to refuse to disclose identifying information on research participation in any civil, criminal, administrative, legislative, or other proceeding, whether at the federal, state, or local level. By protecting researchers and institutions from being compelled to disclose information that would identify research participants, Certificates of Confidentiality help achieve the research objectives and promote participation in studies by helping assure confidentiality and privacy to participants.

10.4 Future Use of Stored Specimens and Data

Participants are consented at enrollment for permission to indefinite storage and future use of specimens and data. Samples, specimens, and data collected under this protocol may be used to study aspects of immunity to influenza, influenza infection, and disease related to influenza infection. Genetic testing will not be performed in the future on stored samples.

Storage and Tracking: Access to and tracking of stored samples and data will be secured and limited as described above (section 10.3).

Disposition:

- In the future, other investigators (both at NIH and outside) may wish to use these samples and/or data for research purposes. If the planned research falls within the category of "human subjects research" on the part of the NIH researchers, IRB review and approval will be obtained. This includes the NIH researchers sending out coded and linked samples or data and getting results that they can link back to their subjects.

10.5 Safety Oversight

Safety oversight is described in section 8.4.5.

10.6 Clinical Monitoring

As per ICH E6(R2) GCP guidelines section 5.18 and FDA 21 CFR 312.50, clinical protocols are required to be adequately monitored by the study sponsor. This study monitoring will be conducted according to the "NIAID Intramural Clinical Monitoring Guidelines." Monitors under contract to the NIAID/Office of Clinical Research Policy and Regulatory Operations (OCRPRO) will visit the clinical research site to monitor aspects of the study in accordance with the appropriate regulations and the approved protocol. The objectives of a monitoring visit will be: 1) to verify the existence of signed informed consent documents and documentation of the consent process for each monitored participant; 2) to verify the prompt and accurate recording of all monitored data points in CRIMSON and prompt reporting of all SAEs; 3) to compare abstracted information entered into CRIMSON with individual participants' records and source documents (participants' charts, laboratory analyses and test results, physicians' progress notes, nurses' notes, and any other relevant original participant information); and 4) to help ensure investigators are in compliance with the protocol. The monitors also will inspect the clinical site regulatory files to ensure that regulatory requirements (FDA and OHRP) and applicable guidelines (ICH GCP) are being followed. During the monitoring visits, the investigator (and/or designee) and other study personnel will be available to discuss the study progress and monitoring visit.

The investigator (and/or designee) will make study documents (e.g., consent forms, CRIMSON data abstracts) and pertinent hospital or clinical records, including CRIMSON, readily available for inspection by the FDA, IRB, site monitors, and NIAID staff for confirmation of the study data.

A specific protocol monitoring plan will be discussed with the PI and study staff prior to enrollment of the first participant. The plan will outline the frequency of monitoring visits based on such factors as study enrollment, data collection status, and regulatory obligations.

10.7 Quality Assurance and Quality Control

During the study, the PI and study team will be responsible for implementing a quality management plan. Additionally, the study team will be responsible for completing and submitting a summary report on the quality plan to the NIAID Clinical Director or designee at least annually as detailed in the quality management plan. A courtesy copy will also be sent to CTM.

10.8 Data Handling and Record Keeping

10.8.1 Data Collection and Management Responsibilities

Data collection is the responsibility of the clinical trial staff at the site under the supervision of the PI. The investigator is responsible for ensuring the accuracy, completeness, legibility, and timeliness of the data reported.

Clinical data (including AEs, concomitant medications, and data on expected ARs) and clinical laboratory data will be collected and maintained in CRIMSON and REDCap. Information will be collected directly from participants during study visits and telephone calls. Source documents include all recordings of observations or notations of clinical activities and all reports and records necessary to confirm the data abstracted for this study. Data entry into CRIMSON and REDCap will be performed by authorized individuals. Please refer to

for the REDCap survey.

Data that may potentially unblind the treatment assignment (e.g., treatment allocation) will be handled with special care to ensure that the integrity of the blind is maintained and the potential for bias is minimized. This can include making special provisions, such as segregating the data in question from view by the investigators, sponsor clinical team, or others as appropriate until the time of database lock and unblinding.

10.8.2 Study Records Retention

The investigator is responsible for retaining all essential documents listed in the ICH GCP guidelines. Study records will be maintained by the PI according to the timelines specified in 21 CFR 312.62 or a minimum of 7 years, and in compliance with institutional, IRB, state, and federal medical records retention requirements, whichever is longest. All stored records will be kept confidential to the extent required by federal, state, and local law.

Should the investigator wish to assign the study records to another party and/or move them to another location, the investigator will provide written notification of such intent to OCRPRO/NIAID with the name of the person who will accept responsibility for the transferred records and/or their new location. NIAID will be notified in writing and written OCRPRO/NIAID permission shall be obtained by the site prior to destruction or relocation of research records.

10.9 Protocol Deviations

It is the responsibility of the investigator to use continuous vigilance to identify and report deviations to the NIH IRB according to HRPP Policy 801. All deviations must be addressed in study source documents and reported to CTM. The investigator is responsible for knowing and adhering to the reviewing IRB requirements.

10.9.1 NIH Definition of Protocol Deviation

The definition of a protocol deviation is provided in section 8.4.1.

10.10 Publication and Data Sharing Policy

10.10.1 Human Data Sharing Plan

This study will be conducted in accordance with the following publication and data sharing policies and regulations:

NIH Public Access Policy, which ensures that the public has access to the published results of NIH funded research. It requires scientists to submit final peer-reviewed journal manuscripts that arise from NIH funds to the digital archive PubMed Central upon acceptance for publication.

This study will comply with the NIH Data Sharing Policy and Policy on the Dissemination of NIH-Funded Clinical Trial Information and the Clinical Trials Registration and Results Information Submission rule. As such, this trial will be registered at ClinicalTrials.gov, and

results information from this trial will be submitted to ClinicalTrials.gov. In addition, every attempt will be made to publish results in peer-reviewed journals.

Human data generated in this study will be shared for future research as follows:

- De-identified data in an NIH-funded or approved public repository, including the Gene Expression Omnibus (GEO) database repository.
- Identified data in the Biomedical Translational Research Information System (BTRIS, automatic for activities in the CC).

Data will be shared at the time of publication or shortly thereafter.

Provisions for protection of data privacy and confidentiality are described in section 10.3.

10.10.2 Genomic Data Sharing Plan

This study will comply with the NIH Genomic Data Sharing Policy, which applies to all NIH-funded research that generates large-scale human or non-human genomic data, as well as the use of these data for subsequent research. Large-scale data include genome-wide association studies, single nucleotide polymorphisms arrays, and genome sequence, transcriptomic, epigenomic, and gene expression data.

10.11 Collaborative Agreements

10.11.1 Agreement Type

Not applicable.

10.12 Conflict of Interest Policy

The independence of this study from any actual or perceived influence, such as by the pharmaceutical industry, is critical. Therefore, any actual conflict of interest of persons who have a role in the design, conduct, analysis, publication, or any aspect of this trial will be disclosed and managed. Furthermore, persons who have a perceived conflict of interest will be required to have such conflicts managed in a way that is appropriate to their participation in the design and conduct of this trial. The study leadership will follow NIAID policies and procedures for all study group members to disclose and manage all conflicts of interest and will establish a mechanism for the management of all reported dualities of interest.

11 ABBREVIATIONS

AE	adverse event
AR	adverse reaction
BMGF	Bill & Melinda Gates Foundation
BMI	body mass index
BPL	β-propiolactone
BTRIS	Biomedical Translational Research Information System
CBC	complete blood count

CC	Clinical Center
CFR	Code of Federal Regulations
CRIMSON	Clinical Research Information Management System of the National Institute of Allergy and Infectious Diseases
CSO	Clinical Safety Office
CSU	Clinical Studies Unit
CTM	Clinical Trials Management
DSMB	data and safety monitoring board
EKG	electrocardiogram
ELISA	enzyme-linked immunosorbent assay
FDA	Food and Drug Administration
GBS	Guillain-Barré syndrome
GCP	Good Clinical Practice
GEE	generalized estimating equation
GMP	Good Manufacturing Practice
HA	hemagglutinin
HAI	hemagglutination inhibition
HIV	human immunodeficiency virus
HRPP	Human Research Protection Program
IAV	influenza A virus
ICH	International Council on Harmonisation
IDMRS	Investigational Drug Management Research Section
ILI	influenza-like illness
IM	intramuscular(ly)
IN	intranasal(ly)
IND	investigational new drug
IRB	institutional review board
LDH	lactate dehydrogenase
LID	Laboratory of Infectious Diseases
M2	matrix 2 (surface protein)
MAD	mucosal atomization device
NA	neuraminidase
NAI	neuraminidase inhibition
NIAID	National Institute of Allergy and Infectious Diseases
NIH	National Institutes of Health
OCRPRO	Office of Clinical Research Policy and Regulatory Operations
OHRP	Office for Human Research Protections
PBMC	peripheral blood mononuclear cells
PI	principal investigator
PSEE	protocol-specified exempt event
REDCap	Research Electronic Data Capture (system)
RNA	ribonucleic acid
SAE	serious adverse event
SAM	synthetic absorptive matrices
SAR	suspected adverse reaction
SERF	safety expedited report form

SMM	sponsor medical monitor
SRCP	safety review and communications plan
SUSAR	serious and unexpected suspected adverse reaction
TP	total protein
UP	unanticipated problem
UPnonAE	unanticipated problem that is not an adverse event
US	United States
VDJ	variable, diversity, and joining (genes)
VPES	Viral Pathogenesis and Evolution Section

12 REFERENCES

1. Influenza (seasonal). 2014. (Accessed January 9th, 2020, at http://www.who.int/mediacentre/factsheets/fs211/en/.)

2. Past Seasons Vaccine Effectiveness Estimates. 2019. (Accessed December 13 2019, at https://www.cdc.gov/flu/vaccines-work/past-seasons-estimates.html.)

3. Webster RG, Govorkova EA. Continuing challenges in influenza. Ann N Y Acad Sci 2014;1323:115-39.

4. Taubenberger JK, Kash JC. Influenza virus evolution, host adaptation, and pandemic formation. Cell Host Microbe 2010;7:440-51.

5. Treanor JJ, Talbot HK, Ohmit SE, et al. Effectiveness of seasonal influenza vaccines in the United States during a season with circulation of all three vaccine strains. Clin Infect Dis 2012;55:951-9.

6. Krammer F, Palese P, Steel J. Advances in universal influenza virus vaccine design and antibody mediated therapies based on conserved regions of the hemagglutinin. Curr Top Microbiol Immunol 2015;386:301-21.

7. Morens DM, Taubenberger JK. Making Universal Influenza Vaccines: Lessons From the 1918 Pandemic. J Infect Dis 2019;219:S5-S13.

8. Erbelding EJ, Post DJ, Stemmy EJ, et al. A Universal Influenza Vaccine: The Strategic Plan for the National Institute of Allergy and Infectious Diseases. J Infect Dis 2018;218:347-54.

9. Malik Peiris JS. Avian influenza viruses in humans. Rev Sci Tech 2009;28:161-73.

10. Morens DM, Taubenberger JK, Fauci AS. H7N9 avian influenza A virus and the perpetual challenge of potential human pandemicity. mBio 2013;4.

11. Salk JE, Menke Jr WJ, Francis Jr T. A clinical, epidemiological and immunological evaluation of vaccination against epidemic influenza. American Journal of Epidemiology 1945;42:57-93.

12. Chen K, Magri G, Grasset EK, Cerutti A. Rethinking mucosal antibody responses: IgM, IgG and IgD join IgA. Nat Rev Immunol 2020;20:427-41.

13. McMahon M, Kirkpatrick E, Stadlbauer D, Strohmeier S, Bouvier NM, Krammer F. Mucosal Immunity against Neuraminidase Prevents Influenza B Virus Transmission in Guinea Pigs. MBio 2019;10.

14. Flucelvax Quadrivalent [package insert]. Holly Springs, NC: Seqirus; 2020.

15. DiazGranados CA, Dunning AJ, Kimmel M, et al. Efficacy of high-dose versus standard-dose influenza vaccine in older adults. N Engl J Med 2014;371:635-45.
