# Supplementary material for: Safety and immunogenicity of a BPL-inactivated, whole-virus, universal influenza vaccine: a randomized, double-blinded, double-dummy, placebo-controlled, phase 1 clinical trial
Source: Nat Commun. 2026 Oct 12;17:11539. doi: 10.1038/s41467-026-78583-w (PMC13748664; doi:10.1038/s41467-026-78583-w)
Supplement: Supplementary file 2 [file 41467_2026_78583_MOESM2_ESM.pdf]

nature portfolio

Corresponding author(s): Luca Giurgea

Last updated by author(s): Sep 12, 2026

Reporting Summary

Nature Portfolio wishes to improve the reproducibility of the work that we publish. This form provides structure for consistency and transparency in reporting. For further information on Nature Portfolio policies, see our Editorial Policies and the Editorial Policy Checklist.

Statistics

For all statistical analyses, confirm that the following items are present in the figure legend, table legend, main text, or Methods section.

n/a	Confirmed	
☐	☒	The exact sample size (*n*) for each experimental group/condition, given as a discrete number and unit of measurement
☐	☒	A statement on whether measurements were taken from distinct samples or whether the same sample was measured repeatedly
☐	☒	The statistical test(s) used AND whether they are one- or two-sided<br>*Only common tests should be described solely by name; describe more complex techniques in the Methods section.*
☐	☒	A description of all covariates tested
☐	☒	A description of any assumptions or corrections, such as tests of normality and adjustment for multiple comparisons
☐	☒	A full description of the statistical parameters including central tendency (e.g. means) or other basic estimates (e.g. regression coefficient) AND variation (e.g. standard deviation) or associated estimates of uncertainty (e.g. confidence intervals)
☐	☒	For null hypothesis testing, the test statistic (e.g. *F*, *t*, *r*) with confidence intervals, effect sizes, degrees of freedom and *P* value noted<br>*Give P values as exact values whenever suitable.*
☒	☐	For Bayesian analysis, information on the choice of priors and Markov chain Monte Carlo settings
☒	☐	For hierarchical and complex designs, identification of the appropriate level for tests and full reporting of outcomes
☐	☒	Estimates of effect sizes (e.g. Cohen's *d*, Pearson's *r*), indicating how they were calculated

Our web collection on statistics for biologists contains articles on many of the points above.

Software and code

Policy information about availability of computer code

Data collection	Clinical data were collected in NIAID's electronic medical record system (CRIMSON)
Data analysis	Data analyses were performed using R statistical programing language (version 4.5.1). Figures were generated using R statistical programming language (version 4.5.1) or GraphPad Prism (version 11.0).

For manuscripts utilizing custom algorithms or software that are central to the research but not yet described in published literature, software must be made available to editors and reviewers. We strongly encourage code deposition in a community repository (e.g. GitHub). See the Nature Portfolio guidelines for submitting code & software for further information.

Data

Policy information about availability of data

All manuscripts must include a data availability statement. This statement should provide the following information, where applicable:

- Accession codes, unique identifiers, or web links for publicly available datasets
- A description of any restrictions on data availability
- For clinical datasets or third party data, please ensure that the statement adheres to our policy

The data generated in this study are provided in the Supplementary Information/Source Data file.

Research involving human participants, their data, or biological material

Policy information about studies with human participants or human data. See also policy information about sex, gender (identity/presentation), and sexual orientation and race, ethnicity and racism.

Reporting on sex and gender	The terms sex and gender have been used accurately. Sex data were self-reported and collected from the electronic medical record. Summary data on demographic make-up of trial arms, including sex, are provided (table 1) and raw data include sex data. Sex-based analyses were not performed due to the scope of the study and the small sample size of the study.
Reporting on race, ethnicity, or other socially relevant groupings	Demographic data on race and ethnicity were collected on enrollment per standard hospital procedures. Summary data on demographic make-up of trial arms, including race/ethnicity, are provided and raw data include race/ethnicity data.
Population characteristics	Characteristics of the trial arms are provided in table 1, including age, sex, race/ethnicity, BMI.
Recruitment	Healthy volunteers were recruited from the community through research match, direct contact of prior study participants, and word-of-mouth advertisement.
Ethics oversight	The study was approved by the NIH institutional review board and was overseen by an independent data and safety monitoring board.

Note that full information on the approval of the study protocol must also be provided in the manuscript.

Field-specific reporting

Please select the one below that is the best fit for your research. If you are not sure, read the appropriate sections before making your selection.

- [x] Life sciences
- [ ] Behavioural & social sciences
- [ ] Ecological, evolutionary & environmental sciences

For a reference copy of the document with all sections, see nature.com/documents/nr-reporting-summary-flat.pdf

Life sciences study design

All studies must disclose on these points even when the disclosure is negative.

Sample size	The sample size was calculated based on the probability of detecting safety signals and the target of 15 participants per treatment group was deemed to give 80% power to detect a 12-14% increase over control rates between 5-10%.
Data exclusions	No data exclusions.
Replication	The study procedures, study design, and experimental assays used to generate data are all clearly detailed and were prespecified before unblinding. Research staff conducting assays were blinded to treatment group. The study was monitored by an independent DSMB and study monitor to ensure data reporting was accurate and complete. Technicians performed multiple technical replicates to ensure reproducibility of assay data. Raw data are provided to ensure reproducibility of statistical analyses.
Randomization	The randomization scheme was created by the study statistician using R statistical programing language and conveyed to an unblinded study pharmacist. Blocks of size 9 were used to ensure that within each block, 3 participants were assigned to each of the three groups.
Blinding	For all experiments, investigators, clinical staff, and technical staff performing data collection and analysis of samples were blinded to treatment group assignment.

Reporting for specific materials, systems and methods

We require information from authors about some types of materials, experimental systems and methods used in many studies. Here, indicate whether each material, system or method listed is relevant to your study. If you are not sure if a list item applies to your research, read the appropriate section before selecting a response.

Materials & experimental systems

n/a	Involved in the study	
x		Antibodies
	x	Eukaryotic cell lines
x		Palaeontology and archaeology
x		Animals and other organisms
	x	Clinical data
x		Dual use research of concern
x		Plants

Methods

n/a	Involved in the study	
x		ChIP-seq
x		Flow cytometry
x		MRI-based neuroimaging

Eukaryotic cell lines

Policy information about cell lines and Sex and Gender in Research

Cell line source(s)	BPL-1357 vaccine was manufactured in certified Vero cells [working cell bank (WCB) lot #535263-WCB1, manufactured from master cell bank (MCB) lot # 426068-1, from World Health Organization Vero cells (134th passage, October 1987, No. 1208) sourced from African green monkey kidney cells. Sex is female.<br>MDCK cells were used to perform Micro-neutralization assay. The cells were obtained from ATCC-Catalog: CCL-34- MDCK (NBL-2). Kidney cells were Isolated from an adult female dog (Canis familiaris).
Authentication	Characterization and testing of the MCB and WCB was performed as recommended in the FDA Points to Consider in the Characterization of Cell Lines Used to Produce Biologicals and ICH guidance documents (Q5A and Q5D). This testing included isoenzyme and cytogenetic analyses to confirm cells are African green monkey origin.<br>Testing to detect mycoplasma contamination, species determination and sterility were completed by ATCC
Mycoplasma contamination	Cell lines tested negative for mycoplasma
Commonly misidentified lines (See ICLAC register)	*Name any commonly misidentified cell lines used in the study and provide a rationale for their use.*

Clinical data

Policy information about clinical studies

All manuscripts should comply with the ICMJE guidelines for publication of clinical research and a completed CONSORT checklist must be included with all submissions.

Clinical trial registration	NCT05027932
Study protocol	Study protocol provided with this submission.
Data collection	The study was conducted at the NIH clinical center in Bethesda, Maryland and data were collected between June 2022 and June 2023. Screening occurred between May 2022 and October 2022.
Outcomes	Safety outcomes (AEs and SAEs) used standard definitions and were predefined in the study protocol before study initiation. Safety outcomes were collected by blinded research staff and were compared using tests of proportions (statistical analysis pre-specified in statistical analysis section of protocol). Secondary immunogenicity outcomes were pre-specified (ELISA assessments of antibodies from nasal and serum samples collected 28 days after booster vaccination) in the study protocol, conducted by blinded technicians, and were compared using t-tests (pre-sepcified in analysis section of protocol). HAI and NAI assays were also pre-specified (on serum samples collected 28 days after booster dose). Post-hoc response rates to each antigen were also calculated.

Plants

Seed stocks	*Report on the source of all seed stocks or other plant material used. If applicable, state the seed stock centre and catalogue number. If plant specimens were collected from the field, describe the collection location, date and sampling procedures.*
Novel plant genotypes	*Describe the methods by which all novel plant genotypes were produced. This includes those generated by transgenic approaches, gene editing, chemical/radiation-based mutagenesis and hybridization. For transgenic lines, describe the transformation method, the number of independent lines analyzed and the generation upon which experiments were performed. For gene-edited lines, describe the editor used, the endogenous sequence targeted for editing, the targeting guide RNA sequence (if applicable) and how the editor was applied.*
Authentication	*Describe any authentication procedures for each seed stock used or novel genotype generated. Describe any experiments used to assess the effect of a mutation and, where applicable, how potential secondary effects (e.g. second site T-DNA insertions, mosiacism, off-target gene editing) were examined.*
