# Supplementary material for: Safety and immunogenicity of a BPL-inactivated, whole-virus, universal influenza vaccine: a randomized, double-blinded, double-dummy, placebo-controlled, phase 1 clinical trial
Source: Nat Commun. 2026 Oct 12;17:11539. doi: 10.1038/s41467-026-78583-w (PMC13748664; doi:10.1038/s41467-026-78583-w)
Supplement: Supplementary file 3 [file 41467_2026_78583_MOESM3_ESM.pdf]

nature portfolio

Peer Review File

Safety and immunogenicity of a BPL-inactivated, whole-virus, universal influenza vaccine: a randomized, double-blinded, double-dummy, placebo-controlled, phase 1 clinical trial

Corresponding Author: Dr Luca Giurgea

This manuscript has been previously reviewed at another journal. This document only contains information relating to versions considered at Nature Communications.

This file contains all reviewer reports in order by version, followed by all author rebuttals in order by version.

Open Access This Peer Review File is licensed under a Creative Commons Attribution 4.0 International License, which permits use, sharing, adaptation, distribution and reproduction in any medium or format, as long as you give appropriate credit to the original author(s) and the source, provide a link to the Creative Commons license, and indicate if changes were made.
In cases where reviewers are anonymous, credit should be given to 'Anonymous Referee' and the source.
The images or other third party material in this Peer Review File are included in the article's Creative Commons license, unless indicated otherwise in a credit line to the material. If material is not included in the article's Creative Commons license and your intended use is not permitted by statutory regulation or exceeds the permitted use, you will need to obtain permission directly from the copyright holder.
To view a copy of this license, visit https://creativecommons.org/licenses/by/4.0/
